# One-Pot Three-Component Coupling Reaction of *α*-Amino Aryl Ketones, Indoles, and Perbromomethane Under Mild Conditions

**DOI:** 10.3389/fchem.2022.825772

**Published:** 2022-02-04

**Authors:** De Chen, Hao Lu, Yuxuan Liu, Wei Deng, Renhua Qiu, Jiannan Xiang

**Affiliations:** College of Chemistry and Chemical Engineering, Hunan University, Changsha, China

**Keywords:** one-pot three-component, *a*-amino aryl ketones, indoles and perbromomethane, step-economy, C-H bond functionalization

## Abstract

A simple and efficient one-pot three-component cascade reaction of *α-*amino aryl ketones, indoles, and CBr_4_ in moderate to good yields has been developed. This new strategy exhibits excellent mild reaction conditions and step-economy, easily accessible reactants, and simultaneous construction of three different new bonds (C=N, C–C, and N-Br) in a single step. It is worth noting that the protocol developed provides a simple and practical tool for the construction of diverse indole-containing heterocyclic frameworks, indicating its potential applications in medicinal and material chemistry.

**GRAPHICAL ABSTRACT:**
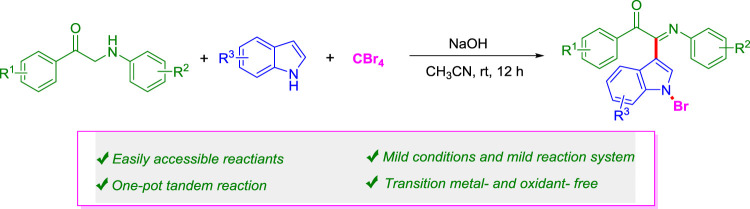


## 1 Introduction

As one of the most important heterocycles, indole is widely present in natural products and medicines due to its remarkable biological activity, such as antibacterial (Van Order and Lindwall., 1942; Bell et al., 1994), anti-obesity (Sashidhara et al., 2012), antimicrobial (Sivaprasad et al., 2006), vaginal spermicide (Paira et al., 2009), and apoptosis in acute myelogenous leukemia (AML) (Contractor et al., 2005). The functionalization of the indole core mainly occurs at the *N*1, *C*2, and *C*3 positions (Bandini and Eichholzer, 2009; Joucla and Djakovitch., 2009; Bartoli et al., 2010; Dalpozzo., 2015; Sandtorv., 2015; Deka et al., 2020). Among them, the *C*3 position modification of indoles is mainly achieved by transition metal–catalyzed C-H bond functionalization (Phipps et al., 2008; Leitch et al., 2017; Ye et al., 2020). In recent years, transition metal–catalyzed C-H functionalization at the *C*3 position of indoles has become a field of extensive research, and tremendous progress has been made in this regard (Kumar et al., 2021).

**SCHEME 1:**
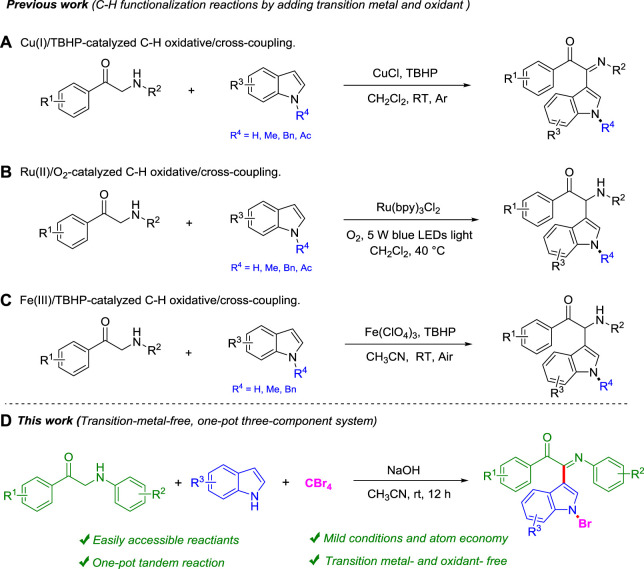
C-H bond functionalization of α-amino aryl ketones and indoles.

From the perspective of simplicity, the oxidative cross-dehydrogenation coupling reaction has become a very good tool for constructing complex molecules through simple reaction materials (Li., 2009; Scheuermann., 2010; Yeung and Dong., 2011; Girard et al., 2014; Song et al., 2017; Wang et al., 2021). Easy-to-prepare and cheap *α*-amino carbonyl units are widely present in many natural products and drug molecules (Ohfune., 1992). However, there are a few reports as the starting material of oxidative cross-dehydrogenation coupling reactions. In 2012, Li group developed a C-H oxidative/cross-coupling strategy of *α*-amino carbonyls with indoles to selectively obtain 2-(1*H*-indol-3-yl)-2-imino-carbonyls under the Cu(I)/TBHP catalytic system (Wu et al., 2012; Scheme 1A). In the same year, the Li group continued to use the visible light photoredox strategy to realize the C-H functionalization of *α-*amino aryl ketones with indoles under Ru (bpy)_3_Cl_2_ catalysis and obtained 2-(1*H*-indol-3-yl)-2-amino-carbonyl compounds (Wang et al., 2012; Scheme 1B). After that, Feng group chose a cheaper Fe(III) catalyst and also realized C-H functionalization of *α-*amino aryl ketones with indoles in 2016 (Zhang et al., 2016; Scheme 1C). However, these synthetic methods all use transition metals and additional oxidants, so it is necessary to explore more green ways. This field aims to develop more efficient, green, and practical C-H functionalization methods and expand its application range. Very recently, our group has also repeatedly reported the application of indoles in organic synthesis, such as direct synthesis of 3,3-diaryl benzofuranones (Tang et al., 2019), *N*-aryl-1-amino indoles (Ou et al., 2021), and 3,3′-diindolylmethanes (DIMs) (Yang et al., 2020) by using indoles as the starting material.

Herein, we report a more effective and green method for the transition metal–free C-H bond functionalization reaction of *α-*amino aryl ketones, indoles, and CBr_4_ under mild conditions (Scheme 1D). This new methodology of green chemistry has several advantages, such as transition metal–free, cheap, and environmental benign reagents, mild reaction conditions, and step-economy.

## 2 Results and Discussion

Initially, the reaction of 1-phenyl-2-(phenylamino)ethan-1-one (1a), CBr_4_ with 1*H*-indole (2a) was selected as a model reaction to optimize the reaction conditions. The different bases, temperatures, times, and solvents were attempted to synthesize (*E*)-2-(1-bromo-1*H*-indol-3-yl)-1-phenyl-2-(phenylimino)ethan-1-one (3a). The results are listed in Table 1. It was found that the reaction readily proceeded in CH_2_Cl_2_ using NaOH as a base leading to produce (*E*)-2-(1-bromo-1*H*-indol-3-yl)-1-phenyl-2-(phenylimino)ethan-1-one (3a) in 37% yield (Table 1, entry 1). This result prompted us to further search for the optimal reaction conditions. Then, we investigated the organic solvents, such as PhMe, DMF, MeCN, DCE, DMSO, THF, 1,4-dioxane, CYH (cyclohexane), Et_2_O, MeOH and EtOH (Table 1, entries 2–12). The most effective solvent was MeCN, which could give 3a in 61% yield (entry 4). Furthermore, temperature also affected the reaction. It was found that room temperature was an appropriate temperature for the reaction, and 3a was obtained in 68% yield (Table 1, entry 13). Higher temperatures did not significantly improve the yield (Table 1, entry 14). Lithium diisopropylamide (LDA) replaced NaOH as the dehydrogenation medium, and it was found that it did not participate in the reaction (Table 1, entry 15). In order to improve 3a yield, we have also checked the reaction using two additional bases, LiOH and KOH. However, the low yield of 3a (37%, 41%) was obtained, respectively (Table 1, entries 16–17). Reaction time played an important role in the reaction. However, in this reaction, no matter whether prolonged or shortened the reaction time, the yield of 3a could not be improved (Table 1, entries 18–19).

**TABLE 1:** Optimization of the reaction conditions.^a^

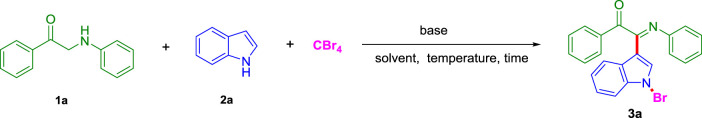				
Entry	Solvent	Temperature (°C)	Base	Yield (%)^b^
1	DCM	40	NaOH	37
2	PhMe	40	NaOH	21
3	DMF	40	NaOH	Trace
4	MeCN	40	NaOH	61
5	DCE	40	NaOH	39
6	DMSO	40	NaOH	Trace
7	THF	40	NaOH	Trace
8	1,4-dioxane	40	NaOH	23
9	CYH	40	NaOH	11
10	Et_2_O	40	NaOH	Trace
11	MeOH	40	NaOH	N.R
12	EtOH	40	NaOH	N.R
13	MeCN	RT	NaOH	68
14	MeCN	50	NaOH	27
15	MeCN	RT	LDA	N.R
16	MeCN	RT	LiOH	41
17	MeCN	RT	KOH	37
18^c^	MeCN	RT	NaOH	28
19^d^	MeCN	RT	NaOH	68

a Reaction condition: All reactions were carried out with 1-phenyl-2-(phenylamino)ethan-1-one (1a) (0.3 mmol), 1*H*-indole (2a) (0.3 mmol), CBr_4_ (0.6 mmol), and base (1.2 mmol) in solvent (2 ml) at certain temperature for 12 h.

b Isolated yield.

c Reaction for 6 h.

d Reaction for 18 h.

On the basis of the optimized reaction conditions, the synthesis of various 2-(1-bromo-1*H*-indol-3-yl)-2-imino-carbonyls was examined by the reactions of *a*-amino aryl ketones and CBr_4_ with 1*H*-indole (2a) in MeCN at room temperature by using NaOH as a base. The results are listed in Table 2. It was found that *α-*amino aryl ketones containing electron-donating groups, such as Me and OMe, on the ortho- or para-positions of aromatic rings afforded the corresponding products in moderate to good yields (3b−3d). The *α-*amino aryl ketones containing electron-withdrawing groups, such as Cl, Br, I, and CF_3_, on the ortho-, meta-, or para-positions of the phenyl group could also give satisfactory yields (3e−3L). Naphthyl *α*-amino aryl ketones can also produce corresponding products in moderate yields (Table 2, 3m). The aromatic amines containing electron-donating groups or electron-withdrawing groups, such as Me and Cl, on the meta- or para-positions could also give satisfactory yield (3n−3p). The 5-Me and 6-Cl of substituted indoles were selected as indole substitution groups to be tested. The corresponding products yields of 3q-3r were found to be good. After comparison, we found that different electron-donating groups and electron-withdrawing groups have little effect on the reaction, and the yields of the reaction are all in the middle to good range. These results indicate that the electronic effect has no obvious influence on the yield. In addition, aryl-alkyl *α*-amino aryl ketones, such as 1-(phenylamino)propan-2-one and 2-(isopropylamino)-1-phenylethan-1-one, were selected as the starting material groups to be tested. Unfortunately, the reactions did not proceed smoothly, and these compounds were unable to obtain the corresponding target products.

**TABLE 2:** Synthesis of 2-(1-bromo-1*H*-indol-3-yl)-2-imino-carbonyls from *α*-amino aryl ketones.^a^ ^,^ ^b^

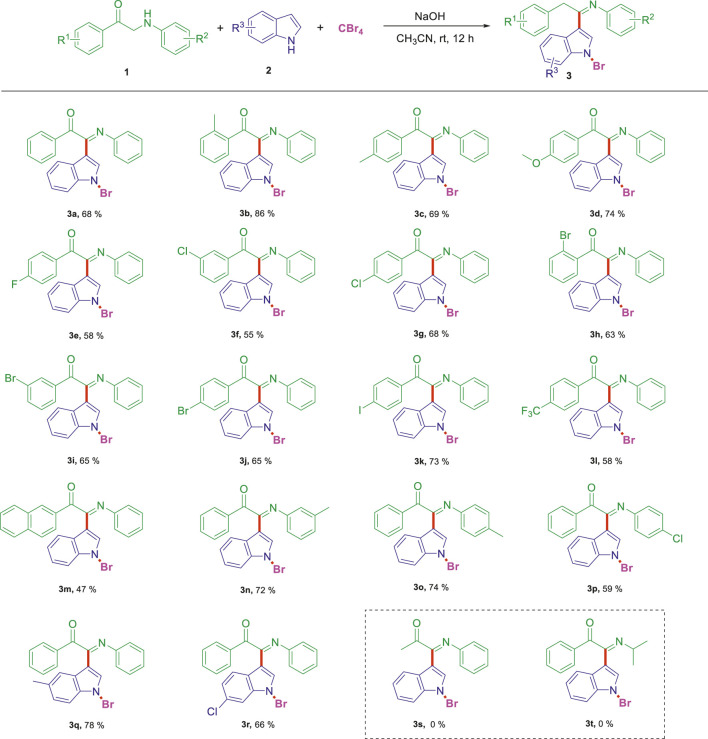

a Reaction condition: All reactions were carried out with *α-*amino aryl ketones (1) (0.3 mmol), 1*H*-indole (2a) (0.3 mmol), CBr_4_ (0.6 mmol), and NaOH (1.2 mmol) in MeCN (2 ml) at room temperature for 12 h.

b Isolated yield.

**SCHEME 2:**
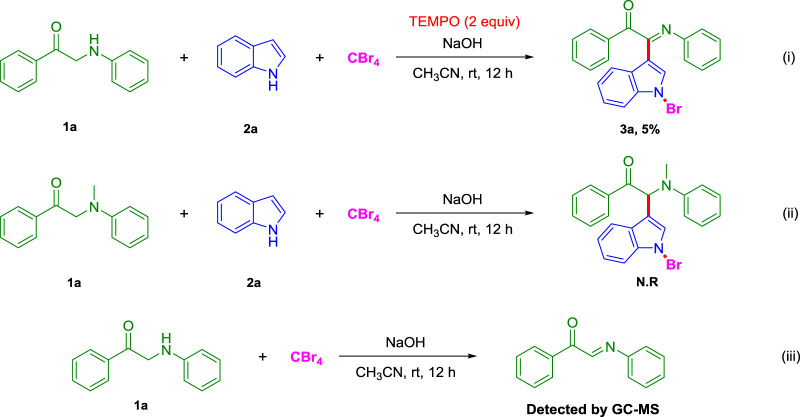
Control experiments.

In order to investigate the reaction mechanism, several control experiments were carried out (Scheme 2). 1) After adding 2,2,6,6-tetramethyl piperidine nitroxide (TEMPO) to the model reaction, the yield of the target product 3a dropped from 68 to 5%, and the reaction was basically completely inhibited. It means that this reaction may be a free radical reaction; 2) when 2-(methyl (phenyl)amino)-1-phenylethan-1-one is used instead of 1-phenyl-2-(phenylamino)ethan-1-one (1a) under the standard conditions, the possible product (*S*)-2-(1-bromo-1*H*-indol-3-yl)-2-(methyl (phenyl)amino)-1-phenylethan-1-one was not observed; 3) in addition, the 1-phenyl-2-(phenylamino)ethan-1-one (1a) reacted with CBr_4_ for 12 h under standard conditions, and the solution was monitored by GC-MS to detect (*E*)-1-phenyl-2-(phenylimino)ethane-1-one. This result implied that (*E*)-1-phenyl-2-(phenylimino)ethane-1-ketone possibly is also an intermediate of three-component reaction.

**SCHEME 3:**
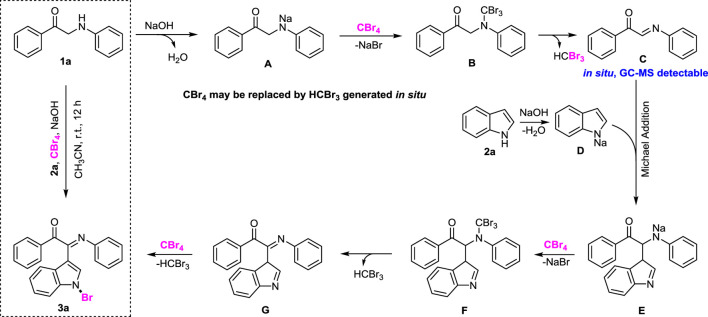
Proposed mechanism for one-pot synthesis of 3a.

Based on the aforementioned control experiments and literature reports (Liu et al., 2018; Zhou et al., 2017), a plausible mechanism is proposed for the one-pot synthesis of 2-(1-bromo-1*H*-indol-3-yl)-2-imino-carbonyls (Scheme 3). Initially, 1-phenyl-2-(phenylamino)ethan-1-one (1a) undergoes nucleophilic substitution reaction under the action of NaOH as a base to obtain intermediates A. Then intermediate A forms intermediate B in the presence of CBr_4_, and intermediate B undergoes reduction and elimination to form imine intermediate C (Liu et al., 2016). At the same time, intermediate C reacts with intermediate D from indole (2a) in the presence of sodium hydroxide to give the Michael addition product E. Then intermediate E forms intermediate F in the presence of CBr_4_, and intermediate F undergoes reduction and elimination to form imine intermediate G. Finally, G can easily afford 3a as a final product involving oxidation of CBr_4_. It should be noted that HCBr_3_ easily reacts with two equivalents of NaOH to form HC(O)Br, which can be captured by aniline as PhNHC(O)H (*N*-formanilide) and is clearly detected by GC-MS (see SI). In addition, since only two equivalents of CBr_4_ were added in the reaction system, we proposed that HCBr_3_ could be working as CBr_4_ to react with A or E in some cases.

## 3 Conclusion

In summary, a mild C-H functionalization for the one-pot three-component synthesis of 2-(1-bromo-1*H*-indol-3-yl)-2-imino-carbonyls is described. The reaction provides an efficient and practical method for the synthesis of biologically significant 2-(1-bromo-1*H*-indol-3-yl)-2-imino-carbonyls in an atom-economic manner under mild and simple reaction conditions. We are currently focusing on applying this C-H bond functionalization to other inert bond cleavage reactions and further exploring research in the construction of more variable compounds.

### 3.1 Experimental Section

#### 3.1.1 General Information

All commercially available reagents were used without further purification. Nuclear magnetic resonance (NMR) spectra were acquired at 298 K on ^1^H NMR (400 MHz) and ^13^C NMR (101 MHz) Bruker NMR spectrometer with the sample dissolved in DMSO-*d*_6_. All values of chemical shift were reported in parts per million (ppm) relative to the solvent signal with the coupling constant (*J*) reported in Hertz. All compounds were characterized by ^1^H NMR, ^13^C NMR, and EI or HRMS (double focusing mass analyzer). Column chromatography was performed on silica gel (300–400 mesh) using petroleum ether (PE)/ethyl acetate (EA) as a developing solvent.

#### 3.1.2 Synthesis of 2-(1-bromo-1*H*-indol-3-yl)-2-imino-carbonyls 3

The mixture of *α*-amino aryl ketones (0.3 mmol), 1*H*-indole (2a) (0.3 mmol), CBr_4_ (0.6 mmol), and NaOH (1.2 mmol) in MeCN (2 ml) was stirred at room temperature for 12 h. The reaction was monitored by TLC. After the completion, the resulting mixture was separated with EA. Water was added for washing, and then 15 ml of EA was used three times for extraction and liquid separation. The collected organic phase was dried with anhydrous Na_2_SO_4_, filtered, and the organic phase was distilled off under reduced pressure. The obtained products 3 were separated by a silica gel column layer, and mobile phase using petroleum ether with the fraction at 60–90°C, and three purified products were obtained. The characterization data of all the products are given as follows.

##### 3.1.2.1 (*E*)-2-(1-bromo-1*H*-indol-3-yl)-1-phenyl-2-(phenylimino)ethan-1-one (3a)


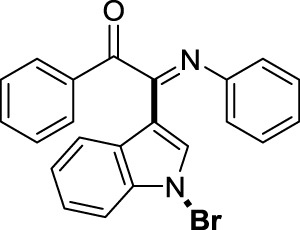


Yellow oil. Yield 62 mg (68%) at 0.3 mmol scale. ^1^H NMR (400 MHz, DMSO-*d*_6_) δ 8.41 (d, *J* = 7.8 Hz, 1H), 7.81 (d, *J* = 7.5 Hz, 2H), 7.71 (s, 1H), 7.64 (t, *J* = 7.2 Hz, 1H), 7.55 (d, *J* = 7.1 Hz, 1H), 7.43 (dq, *J* = 15.1, 7.6 Hz, 4H), 7.14 (t, *J* = 7.2 Hz, 2H), 6.93 (t, *J* = 7.1 Hz, 1H), 6.85 (d, *J* = 7.5 Hz, 2H). ^13^C NMR (101 MHz, DMSO-*d*_6_) δ 190.35, 150.42, 146.08, 136.22, 134.54, 133.64, 130.05, 129.93, 129.35, 129.31, 126.29, 125.58, 124.93, 124.67, 121.35, 119.73, 116.49, 98.36. HRMS-ESI (m/z): calcd for C_22_H_15_BrN_2_O [M + H]^+^: 403.0441; found, 403.0444.

##### 3.1.2.2 (*E*)-2-(1-bromo-1*H*-indol-3-yl)-2-(phenylimino)-1-(*o*-tolyl)ethan-1-one (3b)


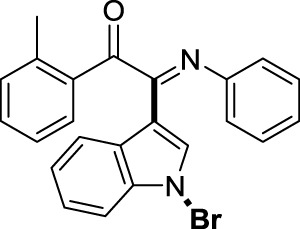


Yellow oil. Yield 87 mg (86%) at 0.3 mmol scale. ^1^H NMR (400 MHz, DMSO-*d*_6_) δ 8.42 (d, *J* = 7.8 Hz, 1H), 7.78 (s, 1H), 7.64 (d, *J* = 7.2 Hz, 1H), 7.54 (d, *J* = 6.2 Hz, 2H), 7.41 (q, *J* = 6.9, 6.3 Hz, 3H), 7.27 – 7.16 (m, 4H), 7.11 (t, *J* = 7.7 Hz, 2H), 6.90 (t, *J* = 7.6 Hz, 1H), 6.76 (d, *J* = 7.7 Hz, 2H), 2.37 (s, 3H). ^13^C NMR (101 MHz, DMSO-*d*_6_) δ 191.94, 151.49, 146.16, 140.96, 134.83, 134.68, 132.84, 132.71, 129.38, 129.14, 127.06, 126.17, 125.85, 124.73, 124.56, 121.15, 119.68, 116.53, 98.25, 21.40. HRMS-ESI (m/z): calcd for C_23_H_17_BrN_2_O [M + H]^+^: 417.0597; found, 417.0587.

##### 3.1.2.3 (*E*)-2-(1-bromo-1*H*-indol-3-yl)-2-(phenylimino)-1-(*p*-tolyl)ethan-1-one (3c)


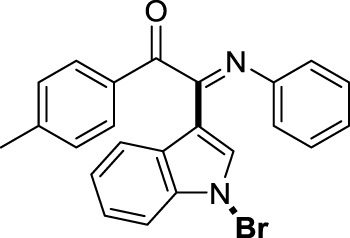


Yellow oil. Yield 70 mg (69%) at 0.3 mmol scale. ^1^H NMR (400 MHz, DMSO-*d*_6_) δ 8.42 (d, *J* = 7.9 Hz, 1H), 7.73 (d, *J* = 7.8 Hz, 2H), 7.67 (s, 1H), 7.55 (d, *J* = 7.3 Hz, 1H), 7.42 (p, *J* = 7.1 Hz, 2H), 7.28 (d, *J* = 7.8 Hz, 2H), 7.17 (t, *J* = 7.5 Hz, 2H), 6.95 (t, *J* = 7.3 Hz, 1H), 6.88 (d, *J* = 7.7 Hz, 2H), 2.30 (s, 3H). ^13^C NMR (101 MHz, DMSO-*d*_6_) δ 189.73, 150.56, 147.39, 146.16, 134.52, 131.27, 130.56, 130.21, 129.33, 129.31, 126.25, 125.51, 124.89, 124.61, 121.35, 119.72, 116.47, 98.30, 21.87. HRMS-ESI (m/z): calcd for C_23_H_17_BrN_2_O [M + H]^+^: 417.0597; found, 417.0603.

##### 3.1.2.4 (*E*)-2-(1-bromo-1*H*-indol-3-yl)-1-(4-methoxyphenyl)-2-(phenylimino)ethan-1-one (3d)


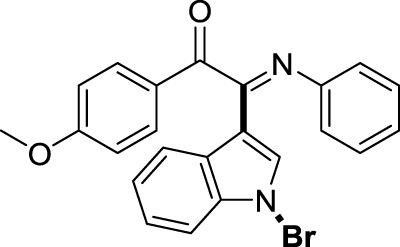


Yellow oil. Yield 78 mg (74%) at 0.3 mmol scale. ^1^H NMR (400 MHz, DMSO-*d*_6_) δ 8.41 (d, *J* = 8.1 Hz, 1H), 7.79 (d, *J* = 8.8 Hz, 2H), 7.65 (s, 1H), 7.53 (d, *J* = 8.2 Hz, 1H), 7.45 – 7.35 (m, 2H), 7.16 (t, *J* = 7.8 Hz, 2H), 6.95 (dd, *J* = 13.3, 8.1 Hz, 3H), 6.87 (d, *J* = 7.5 Hz, 2H), 3.76 (s, 3H). ^13^C NMR (101 MHz, DMSO-*d*_6_) δ 187.75, 165.04, 150.26, 145.79, 134.02, 132.27, 128.83, 128.79, 126.15, 125.71, 125.02, 124.34, 124.04, 120.83, 119.19, 115.97, 114.85, 97.69, 55.79. HRMS-ESI (m/z): calcd for C_23_H_17_BrN_2_O_2_ [M + H]^+^: 433.0546; found, 433.0562.

##### 3.1.2.5 (*E*)-2-(1-bromo-1*H*-indol-3-yl)-1-(4-fluorophenyl)-2-(phenylimino)ethan-1-one (3e)


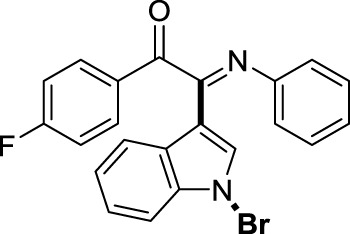


Yellow oil. Yield 73 mg (58%) at 0.3 mmol scale. ^1^H NMR (400 MHz, DMSO-*d*_6_) δ 8.46 (d, *J* = 7.6 Hz, 1H), 7.96 (d, *J* = 6.7 Hz, 2H), 7.78 (s, 1H), 7.59 (d, *J* = 7.3 Hz, 1H), 7.47 (q, *J* = 8.3 Hz, 2H), 7.33 (t, *J* = 8.6 Hz, 2H), 7.20 (t, *J* = 7.7 Hz, 2H), 6.98 (t, *J* = 7.3 Hz, 1H), 6.88 (d, *J* = 7.3 Hz, 2H). ^13^C NMR (101 MHz, DMSO-*d*_6_) δ 188.77, 167.96, 150.26, 146.08, 134.57, 133.47 (d, *J* = 10.3 Hz), 130.57 (d, *J* = 2.5 Hz), 129.35, 126.30, 125.72, 124.97, 124.71, 121.30, 119.71, 117.33, 117.11, 116.58, 98.39. HRMS-ESI (m/z): calcd for C_22_H_14_BrFN_2_O [M + H]^+^: 421.0346; found, 421.0337.

##### 3.1.2.6 (*E*)-2-(1-bromo-1*H*-indol-3-yl)-1-(3-chlorophenyl)-2-(phenylimino)ethan-1-one (3f)


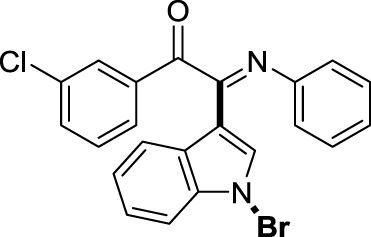


Yellow oil. Yield 72 mg (55%) at 0.3 mmol scale. ^1^H NMR (400 MHz, DMSO-*d*_6_) δ 8.48 (d, *J* = 7.9 Hz, 1H), 7.85 – 7.78 (m, 3H), 7.74 (d, *J* = 8.1 Hz, 1H), 7.59 (d, *J* = 7.7 Hz, 1H), 7.49 (tt, *J* = 15.1, 7.3 Hz, 3H), 7.20 (t, *J* = 7.3 Hz, 2H), 6.99 (t, *J* = 7.5 Hz, 1H), 6.88 (d, *J* = 7.8 Hz, 2H). ^13^C NMR (101 MHz, DMSO-*d*_6_) δ 189.35, 149.82, 145.98, 135.82, 135.36, 134.64, 134.61, 131.92, 129.68, 129.43, 129.31, 128.90, 126.32, 125.89, 125.10, 124.76, 121.34, 119.70, 116.68, 98.44. HRMS-ESI (m/z): calcd for C_22_H_14_BrClN_2_O [M + H]^+^: 437.0051; found, 437.0052.

##### 3.1.2.7 (*E*)-2-(1-bromo-1*H*-indol-3-yl)-1-(4-chlorophenyl)-2-(phenylimino)ethan-1-one (3g)


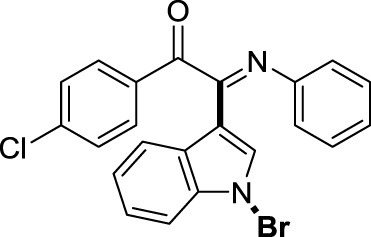


Yellow oil. Yield 73 mg (68%) at 0.3 mmol scale. ^1^H NMR (400 MHz, DMSO-*d*_6_) δ 8.44 (d, *J* = 8.0 Hz, 1H), 7.82 (d, *J* = 8.4 Hz, 2H), 7.75 (s, 1H), 7.52 (dd, *J* = 12.0, 7.9 Hz, 3H), 7.42 (p, *J* = 7.1 Hz, 2H), 7.15 (t, *J* = 7.7 Hz, 2H), 6.94 (t, *J* = 7.4 Hz, 1H), 6.84 (d, *J* = 7.8 Hz, 2H). ^13^C NMR (101 MHz, DMSO-*d*_6_) δ 189.29, 150.10, 146.00, 141.18, 134.59, 132.40, 131.90, 130.09, 129.39, 129.37, 126.29, 125.68, 125.02, 124.71, 121.33, 119.70, 116.59, 98.47. HRMS-ESI (m/z): calcd for C_22_H_14_BrClN_2_O_2_ [M + H]^+^: 437.0051; found, 437.0059.

##### 3.1.2.8 (*E*)-2-(1-bromo-1*H*-indol-3-yl)-1-(2-bromophenyl)-2-(phenylimino)ethan-1-one (3 h)


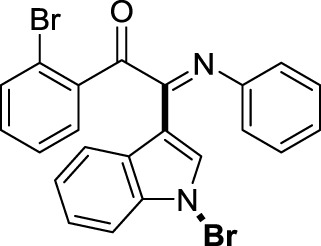


Yellow oil. Yield 91 mg (63%) at 0.3 mmol scale. ^1^H NMR (400 MHz, DMSO-*d*_6_) δ 8.47 (d, *J* = 7.9 Hz, 1H), 7.86 (d, *J* = 7.7 Hz, 2H), 7.75 (d, *J* = 2.0 Hz, 1H), 7.68 (t, *J* = 7.5 Hz, 1H), 7.59 (d, *J* = 7.5 Hz, 1H), 7.47 (dq, *J* = 15.0, 7.4 Hz, 4H), 7.19 (t, *J* = 7.7 Hz, 2H), 6.97 (t, *J* = 7.5 Hz, 1H), 6.90 (d, *J* = 7.6 Hz, 2H). ^13^C NMR (101 MHz, DMSO-*d*_6_) δ 190.37, 150.45, 146.11, 136.22, 134.57, 133.66, 130.08, 129.93, 129.38, 129.32, 126.29, 125.61, 124.93, 124.67, 121.37, 119.74, 116.54, 98.39. HRMS-ESI (m/z): calcd for C_22_H_14_Br_2_N_2_O [M + H]^+^: 480.9546; found, 480.9568.

##### 3.1.2.9 (*E*)-2-(1-bromo-1*H*-indol-3-yl)-1-(3-bromophenyl)-2-(phenylimino)ethan-1-one (3i)


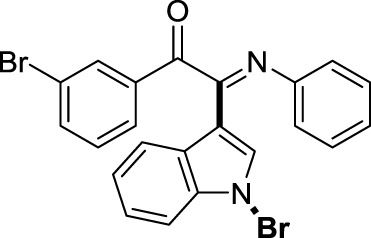


Yellow oil. Yield 94 mg (65%) at 0.3 mmol scale. ^1^H NMR (400 MHz, DMSO-*d*_6_) δ 7.69 (d, *J* = 8.0 Hz, 1H), 7.16 (s, 1H), 7.09 – 7.00 (m, 3H), 6.79 (d, *J* = 7.5 Hz, 1H), 6.66 (q, *J* = 7.8 Hz, 3H), 6.42 (t, *J* = 7.7 Hz, 2H), 6.20 (t, *J* = 7.5 Hz, 1H), 6.09 (d, *J* = 7.4 Hz, 3H). ^13^C NMR (101 MHz, DMSO-*d*_6_) δ 189.27, 149.77, 145.99, 138.67, 135.48, 134.65, 132.08, 131.80, 129.64, 129.43, 126.30, 125.89, 125.11, 124.75, 122.97, 121.34, 119.70, 116.69, 98.45. HRMS-ESI (m/z): calcd for C_22_H_14_Br_2_N_2_O [M + H]^+^: 480.9546; found, 480.9549.

##### 3.1.2.10 (*E*)-2-(1-bromo-1*H*-indol-3-yl)-1-(4-bromophenyl)-2-(phenylimino)ethan-1-one (3j)


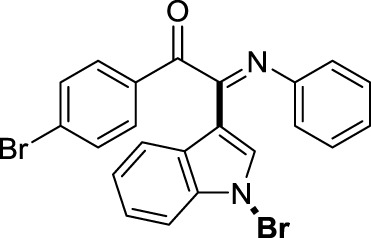


Yellow oil. Yield 78 mg (65%) at 0.3 mmol scale. ^1^H NMR (400 MHz, DMSO-*d*_6_) δ 8.44 (d, *J* = 8.0 Hz, 1H), 7.79 – 7.70 (m, 3H), 7.67 (d, *J* = 8.5 Hz, 2H), 7.55 (d, *J* = 7.3 Hz, 1H), 7.43 (p, *J* = 7.1 Hz, 2H), 7.16 (t, *J* = 7.7 Hz, 2H), 6.95 (t, *J* = 7.4 Hz, 1H), 6.84 (d, *J* = 7.9 Hz, 2H). ^13^C NMR (101 MHz, DMSO-*d*_6_) δ 189.54, 150.08, 145.99, 134.58, 133.05, 132.70, 131.88, 130.69, 129.38, 126.30, 125.70, 125.03, 124.72, 121.33, 119.71, 116.59, 98.47. HRMS-ESI (m/z): calcd for C_22_H_14_Br_2_N_2_O [M + H]^+^: 480.9546; found, 480.9565.

##### 3.1.2.11 (*E*)-2-(1-bromo-1*H*-indol-3-yl)-1-(4-iodophenyl)-2-(phenylimino)ethan-1-one (3k)


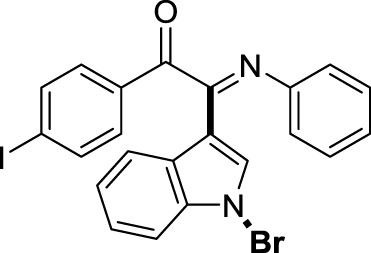


Yellow solid. Yield 98 mg (73%) at 0.3 mmol scale. ^1^H NMR (400 MHz, DMSO-*d*_6_) δ 8.44 (d, *J* = 7.8 Hz, 1H), 7.87 (d, *J* = 7.7 Hz, 2H), 7.74 (s, 1H), 7.57 (d, *J* = 7.7 Hz, 3H), 7.44 (p, *J* = 7.0 Hz, 2H), 7.18 (t, *J* = 7.3 Hz, 2H), 6.97 (t, *J* = 7.2 Hz, 1H), 6.86 (d, *J* = 7.6 Hz, 2H). ^13^C NMR (101 MHz, DMSO-*d*_6_) δ 189.93, 150.11, 145.99, 138.92, 134.56, 132.92, 131.37, 129.38, 126.30, 125.65, 125.02, 124.72, 121.34, 119.72, 116.57, 106.15, 98.47. HRMS-ESI (m/z): calcd for C_22_H_14_BrIN_2_O [M + H]^+^: 528.9407; found, 528.9415.

##### 3.1.2.12 (*E*)-2-(1-bromo-1*H*-indol-3-yl)-2-(phenylimino)-1-(4-(trifluoromethyl)phenyl)ethan-1-one (3l)


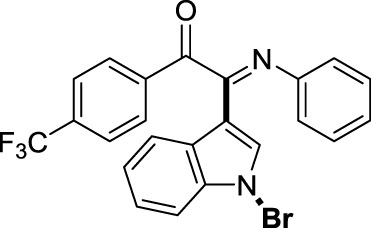


Yellow solid. Yield 82 mg (58%) at 0.3 mmol scale. ^1^H NMR (400 MHz, DMSO-*d*_6_) δ 8.52 (d, *J* = 7.9 Hz, 1H), 8.06 (d, *J* = 8.1 Hz, 2H), 7.87 (d, *J* = 7.1 Hz, 3H), 7.61 (d, *J* = 7.4 Hz, 1H), 7.50 (p, *J* = 7.2 Hz, 2H), 7.20 (t, *J* = 7.8 Hz, 2H), 6.99 (t, *J* = 7.5 Hz, 1H), 6.89 (d, *J* = 7.6 Hz, 2H). ^13^C NMR (101 MHz, DMSO-*d*_6_) δ 189.84, 149.91, 145.85, 136.68, 130.93, 129.41, 126.86, 126.73, 126.37, 125.85, 125.14 (d, *J* = 4.1 Hz), 124.83, 121.37, 119.73, 116.68, 98.57. HRMS-ESI (m/z): calcd for C_23_H_14_BrF_3_N_2_O [M + H]^+^: 471.0314; found, 471.0322.

##### 3.1.2.13 (*E*)-2-(1-bromo-1*H*-indol-3-yl)-1-(naphthalen-2-yl)-2-(phenylimino)ethan-1-one (3m)


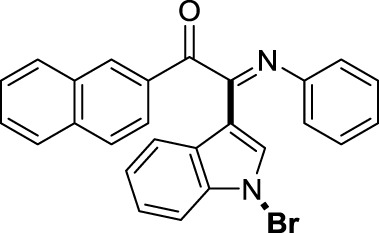


Yellow oil. Yield 52 mg (47%) at 0.3 mmol scale. ^1^H NMR (400 MHz, DMSO-*d*_6_) δ 8.56 (s, 1H), 8.46 (d, *J* = 7.6 Hz, 1H), 8.14 (d, *J* = 8.1 Hz, 1H), 7.94 (t, *J* = 8.0 Hz, 2H), 7.83 (d, *J* = 8.6 Hz, 1H), 7.78 (s, 1H), 7.67 (t, *J* = 7.2 Hz, 1H), 7.57 (t, *J* = 7.8 Hz, 2H), 7.44 (q, *J* = 7.8, 7.4 Hz, 2H), 7.09 (t, *J* = 7.3 Hz, 2H), 6.91 (d, *J* = 7.5 Hz, 2H), 6.86 (t, *J* = 7.1 Hz, 1H). ^13^C NMR (101 MHz, DMSO-*d*_6_) δ 190.38, 150.51, 146.35, 136.45, 134.71, 134.16, 132.42, 131.11, 130.65, 130.61, 129.71, 129.38, 129.29, 128.26, 127.89, 126.23, 125.79, 124.82, 124.62, 123.54, 121.27, 119.71, 116.59, 98.29. HRMS-ESI (m/z): calcd for C_26_H_17_BrN_2_O [M + H]^+^: 453.0597; found, 453.0604.

##### 3.1.2.14 (*E*)-2-(1-bromo-1*H*-indol-3-yl)-1-phenyl-2-(*m*-tolylimino)ethan-1-one (3n)


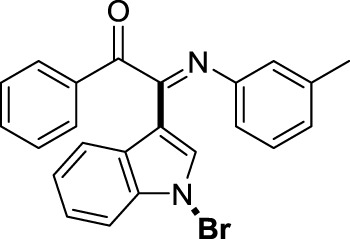


Yellow oil. Yield 90 mg (72%) at 0.3 mmol scale. ^1^H NMR (400 MHz, DMSO-*d*_6_) δ 8.48 (d, *J* = 8.0 Hz, 1H), 7.86 (d, *J* = 7.7 Hz, 2H), 7.75 (s, 1H), 7.69 (t, *J* = 7.5 Hz, 1H), 7.59 (d, *J* = 7.4 Hz, 1H), 7.48 (dt, *J* = 27.2, 7.5 Hz, 4H), 7.06 (t, *J* = 7.8 Hz, 1H), 6.79 (d, *J* = 7.6 Hz, 1H), 6.74 (s, 1H), 6.68 (d, *J* = 7.7 Hz, 1H), 2.16 (s, 3H). ^13^C NMR (101 MHz, DMSO-*d*_6_) δ 190.41, 150.27, 146.03, 138.64, 136.18, 134.56, 133.74, 130.05, 129.91, 129.35, 129.13, 126.26, 125.64, 125.59, 124.64, 122.09, 119.72, 118.35, 116.52, 98.29, 21.28. HRMS-ESI (m/z): calcd for C_23_H_17_BrN_2_O [M + H]^+^: 417.0597; found, 417.0588.

##### 3.1.2.15 (*E*)-2-(1-bromo-1*H*-indol-3-yl)-1-phenyl-2-(*p*-tolylimino)ethan-1-one (3o)


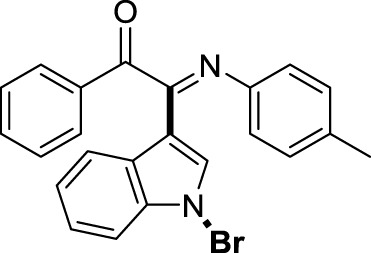


Yellow oil. Yield 92 mg (74%) at 0.3 mmol scale. ^1^H NMR (400 MHz, DMSO-*d*_6_) δ 8.47 (d, *J* = 8.0 Hz, 1H), 7.87 (d, *J* = 7.3 Hz, 2H), 7.73 (s, 1H), 7.69 (t, *J* = 7.5 Hz, 1H), 7.59 (d, *J* = 7.3 Hz, 1H), 7.51 (t, *J* = 7.3 Hz, 2H), 7.46 (t, *J* = 8.3 Hz, 2H), 7.00 (d, *J* = 7.5 Hz, 2H), 6.82 (d, *J* = 7.7 Hz, 2H), 2.16 (s, 3H). ^13^C NMR (101 MHz, DMSO-*d*_6_) δ 190.68, 150.23, 143.50, 136.24, 134.54, 134.02, 133.60, 130.06, 129.98, 129.84, 129.33, 126.24, 125.54, 124.59, 121.32, 119.71, 116.49, 98.22, 79.66, 20.80. HRMS-ESI (m/z): calcd for C_23_H_17_BrN_2_O [M + H]^+^: 417.0597; found, 417.0588.

##### 3.1.2.16 (*E*)-2-(1-bromo-1*H*-indol-3-yl)-2-((4-chlorophenyl)imino)-1-phenylethan-1-one (3p)


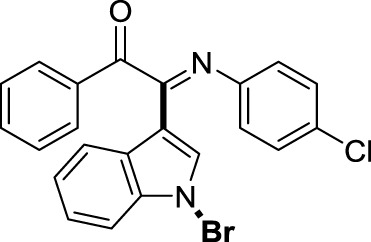


Yellow oil. Yield 77 mg (59%) at 0.3 mmol scale. ^1^H NMR (400 MHz, DMSO-*d*_6_) δ 8.48 (d, *J* = 8.2 Hz, 1H), 7.87 (d, *J* = 7.7 Hz, 2H), 7.72 (d, *J* = 7.6 Hz, 2H), 7.54 (t, *J* = 7.6 Hz, 2H), 7.43 – 7.32 (m, 3H), 7.26 (d, *J* = 8.9 Hz, 2H), 6.92 (d, *J* = 8.0 Hz, 2H), 6.81 (d, *J* = 3.4 Hz, 1H). ^13^C NMR (101 MHz, DMSO-*d*_6_) δ 190.50, 151.77, 145.51, 136.37, 135.07, 133.67, 130.85, 130.12, 129.96, 129.25, 128.81, 126.64, 125.00, 124.07, 123.27, 121.76, 116.48, 109.32. HRMS-ESI (m/z): calcd for C_22_H_14_BrClN_2_O [M + H]^+^: 437.0051; found, 437.0040.

##### 3.1.2.17 (*E*)-2-(1-bromo-5-methyl-1*H*-indol-3-yl)-1-phenyl-2-(phenylimino)ethan-1-one (3q)


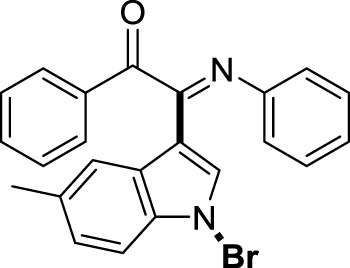


Yellow solid. M.P. 158 – 159°C. Yield 97 mg (78%) at 0.3 mmol scale. ^1^H NMR (400 MHz, DMSO-*d*_6_) δ 8.33 (d, *J* = 8.6 Hz, 1H), 7.84 (d, *J* = 7.7 Hz, 2H), 7.68 (t, *J* = 6.0 Hz, 2H), 7.50 (t, *J* = 7.7 Hz, 2H), 7.37 (s, 1H), 7.29 (d, *J* = 8.5 Hz, 1H), 7.18 (t, *J* = 7.7 Hz, 2H), 6.96 (t, *J* = 7.5 Hz, 1H), 6.88 (d, *J* = 7.7 Hz, 2H), 2.47 (s, 3H). ^13^C NMR (101 MHz, DMSO-*d*_6_) δ 190.43, 150.37, 146.19, 136.20, 134.08, 133.68, 132.83, 130.04, 129.94, 129.55, 129.31, 127.57, 125.55, 124.86, 121.40, 119.40, 116.26, 98.15, 21.41. HRMS-ESI (m/z): calcd for C_23_H_17_BrN_2_O [M + H]^+^: 417.0597; found, 417.0593.

##### 3.1.2.18 (*E*)-2-(1-bromo-6-chloro-1*H*-indol-3-yl)-1-phenyl-2-(phenylimino)ethan-1-one (3r)


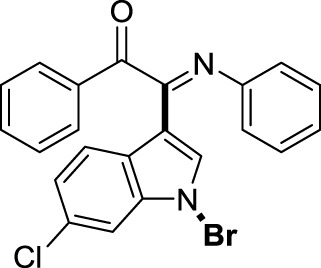


Yellow oil. Yield 86 mg (66%) at 0.3 mmol scale. ^1^H NMR (400 MHz, DMSO-*d*_6_) δ 8.65 (t, *J* = 2.0 Hz, 1H), 7.88 (d, *J* = 6.8 Hz, 2H), 7.78 (d, *J* = 1.9 Hz, 1H), 7.70 (t, *J* = 7.5 Hz, 1H), 7.62 (dd, *J* = 8.5, 1.9 Hz, 1H), 7.55 – 7.49 (m, 3H), 7.21 (t, *J* = 7.8 Hz, 2H), 7.00 (t, *J* = 7.5 Hz, 1H), 6.92 (d, *J* = 7.6 Hz, 2H). ^13^C NMR (101 MHz, DMSO-*d*_6_) δ 189.91, 150.52, 145.86, 136.31, 134.75, 133.53, 130.86, 130.20, 129.92, 129.38, 128.29, 126.76, 125.14, 125.06, 121.34, 121.15, 116.61, 97.94. HRMS-ESI (m/z): calcd for C_22_H_14_BrClN_2_O [M + H]^+^: 437.0051; found, 437.0036.

#### 3.2.1 Synthesis of 2-Bromoacetophenones

The mixture of acetophenone (1.2 g, 10 mmol), *N*-bromosuccinimide (NBS) (1.958 g, 11 mmol), and *p*-toluenesulfonic acid (TsOH) (0.172g, 1 mmol) in MeCN (120 ml) was heated at 60 °C for 24 h. The reaction was monitored by TLC. After the reaction was completed, the solvent was distilled off under reduced pressure, then 30 ml of saturated NaHCO_3_ aqueous solution was poured into the residue, and the mixture was extracted with ethyl acetate (EA) (3 × 20 ml). Next, the organic phases were combined, and anhydrous Na_2_SO_4_ was added for drying. Finally, a rotary evaporator was used to distill the organic solvent under reduced pressure, leaving its residue without further treatment and purification, and it was saved for the next step. Without additional instructions, other substituted acetophenones are similar to this synthesis method.

#### 3.3.1 Synthesis of *a*-amino Aryl Ketones (1a-1 h)

Under nitrogen atmosphere, the mixture of 2-bromoacetophenones (199 mg, 10 mmol), aniline (84 mg, 10 mmol), NaHCO_3_ (93 mg, 1 mmol), and EtOH (40 ml) was added into a dry round-bottom flask and heated at 25°C for 12 h. The reaction was monitored by TLC. After the reaction was completed, the reaction mixture was filtered with suction, and the filtered solid was left in the upper layer, which was washed with EtOH (3 × 5 ml). Finally, it was dissolved in ethyl acetate (EA) and distilled under reduced pressure to obtain an organic phase yellow solid.

## Data Availability

The original contributions presented in the study are included in the article/Supplementary Material; further inquiries can be directed to the corresponding authors.
