# Supplementary material for: One-Pot Three-Component Coupling Reaction of α-Amino Aryl Ketones, Indoles, and Perbromomethane Under Mild Conditions
Source: Front Chem. 2022 Feb 4;10:825772. doi: 10.3389/fchem.2022.825772 (PMC8855049; doi:10.3389/fchem.2022.825772)

Supporting Information

For

One-Pot Three-Component Coupling Reaction of *α*-Amino Aryl Ketones, Indole and Perbromomethane under Mild Conditions

De Chen,[‡] Hao Lu,[‡] Yuxuan Liu,[‡] Wei Deng,* Renhua Qiu* and Jiannan Xiang*

College of Chemistry and Chemical Engineering, Hunan University, Changsha, 410082, China

Corresponding Author： weideng@hnu.edu.cn, renhuaqiu1@hnu.edu.cn,

Jiannaxiang@hnu.edu.cn

Contents

1.General information 3

2.Synthesis of 2-(1-bromo-1*H*-indol-3-yl)-2-imino-carbonyls **3** 3

3. Synthesis of 2-Bromoacetophenones 3

4. The general procedure for the synthesis of α-amino aryl ketones (**1a**-**1r**) 4

5. Analysis data for products 4

6. GC-MS of intermediate **C** and ***N***-formanilide 13

7.References 14

8. ^{1}H, ^{13}C NMR and HRMS spectra for the compounds 15

1. General information.

All commercially available regents were used without further purification. Nuclear magnetic resonance (NMR) spectra was acquired at 298 K on ^{1}H NMR (400 MHz) and ^{13}C NMR (101 MHz) Bruker NMR spectrometer with the sample dissolved in DMSO-d_6. All values of chemical shift were reported in parts per million (ppm) relative to the solvent signal with the coupling constant (J) reported in hertz. All compounds were characterized by ^{1}H NMR, ^{13}C NMR and EI (or HRMS (double focusing mass analyzer). Column chromatography was performed on silica gel (300−400 mesh) using petroleum ether (PE)/ethyl acetate (EA) as developing solvent.

2. Synthesis of 2-(1-bromo-1*H*-indol-3-yl)-2-imino-carbonyls 3.

The mixture of α-amino aryl ketones (0.3 mmol), 1*H*-indole (**2a**) (0.3 mmol), CBr_4 (0.6 mmol) and NaOH (1.2 mmol) in MeCN (2 mL) was stirred at room temperature for 12 h. The reaction was monitored by TLC. After the completion, the resulting mixture was separated with ethyl acetate (EA). Water was added for washing, then 15 mL of ethyl acetate (EA) was used for three times for extraction and liquid separation. The collected organic phase was dried with anhydrous Na_2SO_4, filtered, and the organic phase was distilled off under reduced pressure. The obtained products were separated by silica gel column layer, mobile phase using petroleum ether with the fraction at 60-90°C, and the purified product **3** were obtained. The characterization data of all the products is given below.

3. Synthesis of 2-Bromoacetophenones.

The mixture of acetophenone (1.2 g, 10 mmol), *N*-bromosuccinimide (NBS) (1.958 g, 11 mmol) and *p*-toluenesulfonic acid (TsOH) (0.172g, 1 mmol) in MeCN (120 mL) was heated at 60 °C for 24 h. The reaction was monitored by TLC. After the reaction was completed, the solvent was distilled off under reduced pressure, then 30 mL of saturated $NaHCO_3$ aqueous solution was poured into the residue, and the mixture was extracted with ethyl acetate (EA) (3×20 mL). Next, the organic phases were combined, and anhydrous Na_2SO_4 was added for drying. Finally, use a rotary evaporator to distill the organic solvent under reduced pressure, leaving its residue

without further treatment and purification, and save it for the next step. Without additional instructions, other substituted acetophenones are similar to this synthesis method.

4. The general procedure for the synthesis of α-amino carbonyl compounds (1a-1r)[1-2]

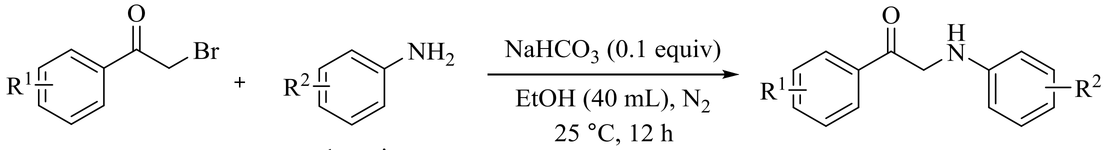

The mixture of 2-bromoacetophenones (10 mmol), anilines (10 mmol), $NaHCO_3$ (93 mg, 1mmol) and EtOH (40 mL) into a dry round bottom flask, and was heated at 25 °C under nitrogen atmosphere for 12 h. The reaction was monitored by TLC. After the reaction was completed, the reaction mixture was filtered with suction, and the filtered solid was left in the upper layer, which was washed with EtOH (3×15 mL). Finally, it was dissolved in ethyl acetate (EA) and distilled under reduced pressure to obtain an organic phase yellow solid (**1a-1r**).

5. Analysis data for products

(*E*)-2-(1-bromo-1*H*-indol-3-yl)-1-phenyl-2-(phenylimino)ethan-1-one (3a):

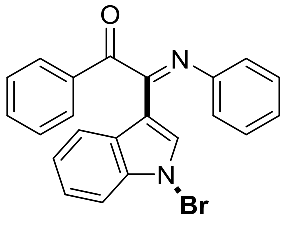

Yellow oil. Yield 62 mg (68%) at 0.3 mmol scale. ^{1}H NMR (400 MHz, DMSO-d_6) δ 8.41 (d, J = 7.8 Hz, 1H), 7.81 (d, J = 7.5 Hz, 2H), 7.71 (s, 1H), 7.64 (t, J = 7.2 Hz, 1H), 7.55 (d, J = 7.1 Hz, 1H), 7.43 (dq, J = 15.1, 7.6 Hz, 4H), 7.14 (t, J = 7.2 Hz, 2H), 6.93 (t, J = 7.1 Hz, 1H), 6.85 (d, J = 7.5 Hz, 2H). ^{13}C NMR (101 MHz, DMSO-d_6) δ 190.35, 150.42, 146.08, 136.22, 134.54, 133.64, 130.05, 129.93, 129.35, 129.31, 126.29, 125.58, 124.93, 124.67, 121.35, 119.73, 116.49, 98.36. HRMS-ESI (m/z):

calcd for $C_{22}H_{15}BrN_2O$, $[M+H]^+$: 403.0441; found, 403.0444.

(*E*)-2-(1-bromo-1*H*-indol-3-yl)-2-(phenylimino)-1-(*o*-tolyl)ethan-1-one (3b):

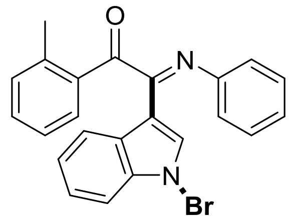

Yellow oil. Yield 87 mg (86%) at 0.3 mmol scale. ^{1}H NMR (400 MHz, DMSO-d_6) δ 8.42 (d, J = 7.8 Hz, 1H), 7.78 (s, 1H), 7.64 (d, J = 7.2 Hz, 1H), 7.54 (d, J = 6.2 Hz, 2H), 7.41 (q, J = 6.9, 6.3 Hz, 3H), 7.27 – 7.16 (m, 4H), 7.11 (t, J = 7.7 Hz, 2H), 6.90 (t, J = 7.6 Hz, 1H), 6.76 (d, J = 7.7 Hz, 2H), 2.37 (s, 3H). ^{13}C NMR (101 MHz, DMSO-d_6) δ 191.94, 151.49, 146.16, 140.96, 134.83, 134.68, 132.84, 132.71, 129.38, 129.14, 127.06, 126.17, 125.85, 124.73, 124.56, 121.15, 119.68, 116.53, 98.25, 21.40. HRMS-ESI (m/z): calcd for $C_{23}H_{17}BrN_2O$, $[M+H]^+$: 417.0597; found, 417.0587.

(*E*)-2-(1-bromo-1*H*-indol-3-yl)-2-(phenylimino)-1-(*p*-tolyl)ethan-1-one (3c):

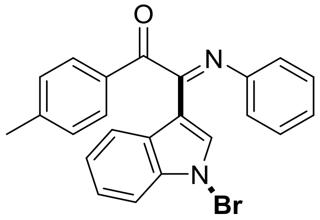

Yellow oil. Yield 70 mg (69%) at 0.3 mmol scale. ^{1}H NMR (400 MHz, DMSO-d_6) δ 8.42 (d, J = 7.9 Hz, 1H), 7.73 (d, J = 7.8 Hz, 2H), 7.67 (s, 1H), 7.55 (d, J = 7.3 Hz, 1H), 7.42 (p, J = 7.1 Hz, 2H), 7.28 (d, J = 7.8 Hz, 2H), 7.17 (t, J = 7.5 Hz, 2H), 6.95 (t, J = 7.3 Hz, 1H), 6.88 (d, J = 7.7 Hz, 2H), 2.30 (s, 3H). ^{13}C NMR (101 MHz, DMSO-d_6) δ 189.73, 150.56, 147.39, 146.16, 134.52, 131.27, 130.56, 130.21, 129.33, 129.31, 126.25, 125.51, 124.89, 124.61, 121.35, 119.72, 116.47, 98.30, 21.87. HRMS-ESI (m/z): calcd for $C_{23}H_{17}BrN_2O$, $[M+H]^+$: 417.0597; found, 417.0603.

(*E*)-2-(1-bromo-1*H*-indol-3-yl)-1-(4-methoxyphenyl)-2-(phenylimino)ethan-1-one (3d):

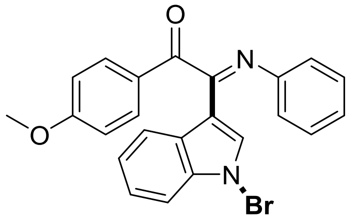

Yellow oil. Yield 78 mg (74%) at 0.3 mmol scale. ^{1}H NMR (400 MHz, DMSO-d_6) δ 8.41 (d, J = 8.1 Hz, 1H), 7.79 (d, J = 8.8 Hz, 2H), 7.65 (s, 1H), 7.53 (d, J = 8.2 Hz, 1H), 7.45 – 7.35 (m, 2H), 7.16 (t, J = 7.8 Hz, 2H), 6.95 (dd, J = 13.3, 8.1 Hz, 3H), 6.87 (d, J = 7.5 Hz, 2H), 3.76 (s, 3H). ^{13}C NMR (101 MHz, DMSO-d_6) δ 187.75, 165.04, 150.26, 145.79, 134.02, 132.27, 128.83, 128.79, 126.15, 125.71, 125.02, 124.34, 124.04, 120.83, 119.19, 115.97, 114.85, 97.69, 55.79. HRMS-ESI (m/z): calcd for $C_{23}H_{17}BrN_2O_2$, $[M+H]^+$: 433.0546; found, 433.0562.

(*E*)-2-(1-bromo-1*H*-indol-3-yl)-1-(4-fluorophenyl)-2-(phenylimino)ethan-1-one (3e):

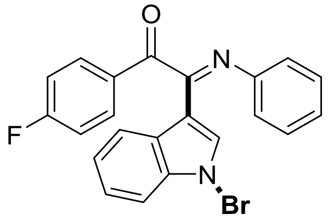

Yellow oil. Yield 73 mg (58%) at 0.3 mmol scale. ^{1}H NMR (400 MHz, DMSO-d_6) δ 8.46 (d, J = 7.6 Hz, 1H), 7.96 (d, J = 6.7 Hz, 2H), 7.78 (s, 1H), 7.59 (d, J = 7.3 Hz, 1H), 7.47 (q, J = 8.3 Hz, 2H), 7.33 (t, J = 8.6 Hz, 2H), 7.20 (t, J = 7.7 Hz, 2H), 6.98 (t, J = 7.3 Hz, 1H), 6.88 (d, J = 7.3 Hz, 2H). ^{13}C NMR (101 MHz, DMSO-d_6) δ 188.77, 167.96, 150.26, 146.08, 134.57, 133.47 (d, J = 10.3 Hz), 130.57 (d, J = 2.5 Hz), 129.35, 126.30, 125.72, 124.97, 124.71, 121.30, 119.71, 117.33, 117.11, 116.58, 98.39. HRMS-ESI (m/z): calcd for $C_{22}H_{14}BrFN_2O$, $[M+H]^+$: 421.0346; found, 421.0337.

(*E*)-2-(1-bromo-1*H*-indol-3-yl)-1-(3-chlorophenyl)-2-(phenylimino)ethan-1-one (3f):

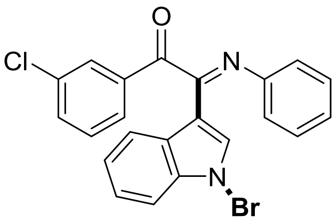

Yellow oil. Yield 72 mg (55%) at 0.3 mmol scale. ^{1}H NMR (400 MHz, DMSO-d_6) δ 8.48 (d, *J* = 7.9 Hz, 1H), 7.85 – 7.78 (m, 3H), 7.74 (d, *J* = 8.1 Hz, 1H), 7.59 (d, *J* = 7.7 Hz, 1H), 7.49 (tt, *J* = 15.1, 7.3 Hz, 3H), 7.20 (t, *J* = 7.3 Hz, 2H), 6.99 (t, *J* = 7.5 Hz, 1H), 6.88 (d, *J* = 7.8 Hz, 2H). ^{13}C NMR (101 MHz, DMSO-d_6) δ 189.35, 149.82, 145.98, 135.82, 135.36, 134.64, 134.61, 131.92, 129.68, 129.43, 129.31, 128.90, 126.32, 125.89, 125.10, 124.76, 121.34, 119.70, 116.68, 98.44. HRMS-ESI (m/z): calcd for $C_{22}H_{14}BrClN_2O$, $[M+H]^+$: 437.0051; found, 437.0052.

(*E*)-2-(1-bromo-1*H*-indol-3-yl)-1-(4-chlorophenyl)-2-(phenylimino)ethan-1-one (3g):

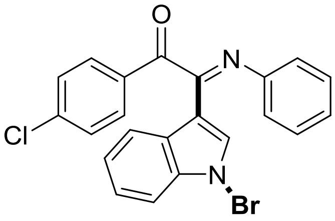

Yellow oil. Yield 73 mg (68%) at 0.3 mmol scale. ^{1}H NMR (400 MHz, DMSO-d_6) δ 8.44 (d, *J* = 8.0 Hz, 1H), 7.82 (d, *J* = 8.4 Hz, 2H), 7.75 (s, 1H), 7.52 (dd, *J* = 12.0, 7.9 Hz, 3H), 7.42 (p, *J* = 7.1 Hz, 2H), 7.15 (t, *J* = 7.7 Hz, 2H), 6.94 (t, *J* = 7.4 Hz, 1H), 6.84 (d, *J* = 7.8 Hz, 2H). ^{13}C NMR (101 MHz, DMSO-d_6) δ 189.29, 150.10, 146.00, 141.18, 134.59, 132.40, 131.90, 130.09, 129.39, 129.37, 126.29, 125.68, 125.02, 124.71, 121.33, 119.70, 116.59, 98.47. HRMS-ESI (m/z): calcd for $C_{22}H_{14}BrClN_2O_2$, $[M+H]^+$: 437.0051; found, 437.0059.

(*E*)-2-(1-bromo-1*H*-indol-3-yl)-1-(2-bromophenyl)-2-(phenylimino)ethan-1-one (3h):

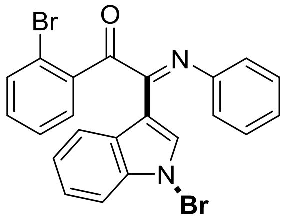

Yellow oil. Yield 91 mg (63%) at 0.3 mmol scale. ^{1}H NMR (400 MHz, DMSO-d_6) δ 8.47 (d, *J* = 7.9 Hz, 1H), 7.86 (d, *J* = 7.7 Hz, 2H), 7.75 (d, *J* = 2.0 Hz, 1H), 7.68 (t, *J* = 7.5 Hz, 1H), 7.59 (d, *J* = 7.5 Hz, 1H), 7.47 (dq, *J* = 15.0, 7.4 Hz, 4H), 7.19 (t, *J* = 7.7 Hz, 2H), 6.97 (t, *J* = 7.5 Hz, 1H), 6.90 (d, *J* = 7.6 Hz, 2H). ^{13}C NMR (101 MHz, DMSO-d_6) δ 190.37, 150.45, 146.11, 136.22, 134.57, 133.66, 130.08, 129.93, 129.38, 129.32, 126.29, 125.61, 124.93, 124.67, 121.37, 119.74, 116.54, 98.39. HRMS-ESI (m/z): calcd for $C_{22}H_{14}Br_2N_2O$, $[M+H]^+$: 480.9546; found, 480.9568.

(*E*)-2-(1-bromo-1*H*-indol-3-yl)-1-(3-bromophenyl)-2-(phenylimino)ethan-1-one (3i)：

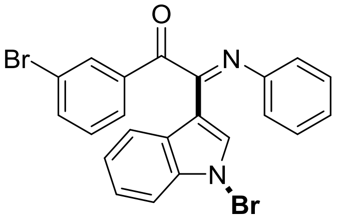

Yellow oil. Yield 94 mg (65%) at 0.3 mmol scale. ^{1}H NMR (400 MHz, DMSO-d_6) δ 7.69 (d, *J* = 8.0 Hz, 1H), 7.16 (s, 1H), 7.09 – 7.00 (m, 3H), 6.79 (d, *J* = 7.5 Hz, 1H), 6.66 (q, *J* = 7.8 Hz, 3H), 6.42 (t, *J* = 7.7 Hz, 2H), 6.20 (t, *J* = 7.5 Hz, 1H), 6.09 (d, *J* = 7.4 Hz, 3H). ^{13}C NMR (101 MHz, DMSO-d_6) δ 189.27, 149.77, 145.99, 138.67, 135.48, 134.65, 132.08, 131.80, 129.64, 129.43, 126.30, 125.89, 125.11, 124.75, 122.97, 121.34, 119.70, 116.69, 98.45. HRMS-ESI (m/z): calcd for $C_{22}H_{14}Br_2N_2O$, $[M+H]^+$: 480.9546; found, 480.9549.

(*E*)-2-(1-bromo-1*H*-indol-3-yl)-1-(4-bromophenyl)-2-(phenylimino)ethan-1-one (3j)：

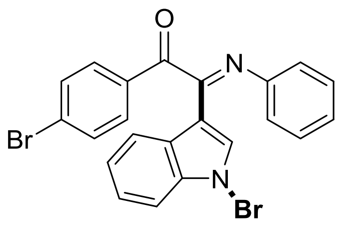

Yellow oil. Yield 78 mg (65%) at 0.3 mmol scale. ^{1}H NMR (400 MHz, DMSO-d_6) δ 8.44 (d, *J* = 8.0 Hz, 1H), 7.79 – 7.70 (m, 3H), 7.67 (d, *J* = 8.5 Hz, 2H), 7.55 (d, *J* = 7.3 Hz, 1H), 7.43 (p, *J* = 7.1 Hz, 2H), 7.16 (t, *J* = 7.7 Hz, 2H), 6.95 (t, *J* = 7.4 Hz, 1H), 6.84 (d, *J* = 7.9 Hz, 2H). ^{13}C NMR (101 MHz, DMSO-d_6) δ 189.54, 150.08, 145.99, 134.58, 133.05, 132.70, 131.88, 130.69, 129.38, 126.30, 125.70, 125.03, 124.72, 121.33, 119.71, 116.59, 98.47. HRMS-ESI (m/z): calcd for $C_{22}H_{14}Br_2N_2O$, $[M+H]^+$: 480.9546; found, 480.9565.

(*E*)-2-(1-bromo-1*H*-indol-3-yl)-1-(4-iodophenyl)-2-(phenylimino)ethan-1-one (3k):

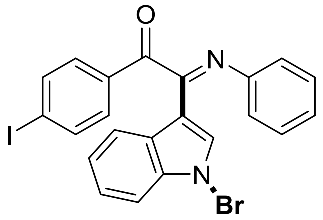

Yellow solid. Yield 98 mg (73%) at 0.3 mmol scale. ^{1}H NMR (400 MHz, DMSO-d_6) δ 8.44 (d, *J* = 7.8 Hz, 1H), 7.87 (d, *J* = 7.7 Hz, 2H), 7.74 (s, 1H), 7.57 (d, *J* = 7.7 Hz, 3H), 7.44 (p, *J* = 7.0 Hz, 2H), 7.18 (t, *J* = 7.3 Hz, 2H), 6.97 (t, *J* = 7.2 Hz, 1H), 6.86 (d, *J* = 7.6 Hz, 2H). ^{13}C NMR (101 MHz, DMSO-d_6) δ 189.93, 150.11, 145.99, 138.92, 134.56, 132.92, 131.37, 129.38, 126.30, 125.65, 125.02, 124.72, 121.34, 119.72, 116.57, 106.15, 98.47. HRMS-ESI (m/z): calcd for $C_{22}H_{14}BrIN_2O$, $[M+H]^+$: 528.9407; found, 528.9415.

(*E*)-2-(1-bromo-1*H*-indol-3-yl)-2-(phenylimino)-1-(4-(trifluoromethyl)phenyl)ethan-1-one (3l):

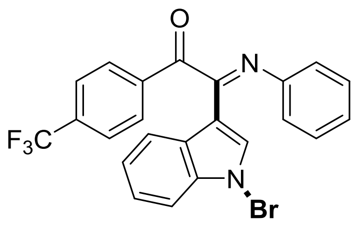

Yellow solid. Yield 82 mg (58%) at 0.3 mmol scale. ^{1}H NMR (400 MHz, DMSO-d_6) δ 8.52 (d, *J* = 7.9 Hz, 1H), 8.06 (d, *J* = 8.1 Hz, 2H), 7.87 (d, *J* = 7.1 Hz, 3H), 7.61 (d, *J* = 7.4 Hz, 1H), 7.50 (p, *J* = 7.2 Hz, 2H), 7.20 (t, *J* = 7.8 Hz, 2H), 6.99 (t, *J* = 7.5 Hz, 1H), 6.89 (d, *J* = 7.6 Hz, 2H). ^{13}C NMR (101 MHz, DMSO-d_6) δ 189.84, 149.91, 145.85, 136.68, 130.93, 129.41, 126.86, 126.73, 126.37, 125.85, 125.14 (d, *J* = 4.1 Hz), 124.83, 121.37, 119.73, 116.68, 98.57. HRMS-ESI (m/z): calcd for $C_{23}H_{14}BrF_3N_2O$, $[M+H]^+$: 471.0314; found, 471.0322.

(*E*)-2-(1-bromo-1*H*-indol-3-yl)-1-(naphthalen-2-yl)-2-(phenylimino)ethan-1-one (3m)：

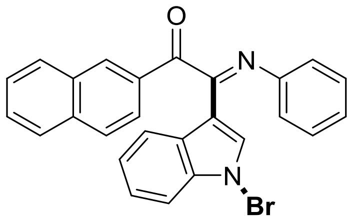

Yellow oil. Yield 52 mg (47%) at 0.3 mmol scale. ^{1}H NMR (400 MHz, DMSO-d_6) δ 8.56 (s, 1H), 8.46 (d, *J* = 7.6 Hz, 1H), 8.14 (d, *J* = 8.1 Hz, 1H), 7.94 (t, *J* = 8.0 Hz, 2H), 7.83 (d, *J* = 8.6 Hz, 1H), 7.78 (s, 1H), 7.67 (t, *J* = 7.2 Hz, 1H), 7.57 (t, *J* = 7.8 Hz, 2H), 7.44 (q, *J* = 7.8, 7.4 Hz, 2H), 7.09 (t, *J* = 7.3 Hz, 2H), 6.91 (d, *J* = 7.5 Hz, 2H), 6.86 (t, *J* = 7.1 Hz, 1H). ^{13}C NMR (101 MHz, DMSO-d_6) δ 190.38, 150.51, 146.35, 136.45, 134.71, 134.16, 132.42, 131.11, 130.65, 130.61, 129.71, 129.38, 129.29, 128.26, 127.89, 126.23, 125.79, 124.82, 124.62, 123.54, 121.27, 119.71, 116.59, 98.29. HRMS-ESI (m/z): calcd for $C_{26}H_{17}BrN_2O$, $[M+H]^+$: 453.0597; found, 453.0604.

(*E*)-2-(1-bromo-1*H*-indol-3-yl)-1-phenyl-2-(m-tolylimino)ethan-1-one (3n)：

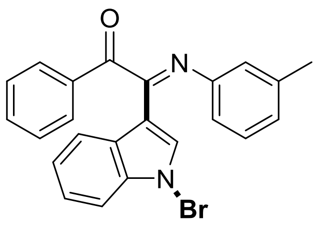

Yellow oil. Yield 90 mg (72%) at 0.3 mmol scale. ^{1}H NMR (400 MHz, DMSO-d_6) δ 8.48 (d, *J* = 8.0 Hz, 1H), 7.86 (d, *J* = 7.7 Hz, 2H), 7.75 (s, 1H), 7.69 (t, *J* = 7.5 Hz, 1H), 7.59 (d, *J* = 7.4 Hz, 1H), 7.48 (dt, *J* = 27.2, 7.5 Hz, 4H), 7.06 (t, *J* = 7.8 Hz, 1H), 6.79 (d, *J* = 7.6 Hz, 1H), 6.74 (s, 1H), 6.68 (d, *J* = 7.7 Hz, 1H), 2.16 (s, 3H). ^{13}C NMR (101 MHz, DMSO-d_6) δ 190.41, 150.27, 146.03, 138.64, 136.18, 134.56, 133.74, 130.05, 129.91, 129.35, 129.13, 126.26, 125.64, 125.59, 124.64, 122.09, 119.72, 118.35, 116.52, 98.29, 21.28. HRMS-ESI (m/z): calcd for $C_{23}H_{17}BrN_2O$, $[M+H]^+$: 417.0597; found, 417.0588.

(*E*)-2-(1-bromo-1*H*-indol-3-yl)-1-phenyl-2-(p-tolylimino)ethan-1-one (3o):

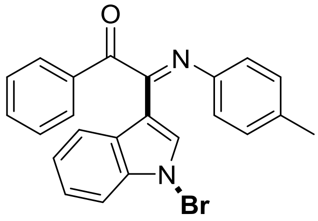

Yellow oil. Yield 92 mg (74%) at 0.3 mmol scale. ^{1}H NMR (400 MHz, DMSO-d_6) δ 8.47 (d, *J* = 8.0 Hz, 1H), 7.87 (d, *J* = 7.3 Hz, 2H), 7.73 (s, 1H), 7.69 (t, *J* = 7.5 Hz, 1H), 7.59 (d, *J* = 7.3 Hz, 1H), 7.51 (t, *J* = 7.3 Hz, 2H), 7.46 (t, *J* = 8.3 Hz, 2H), 7.00 (d, *J* = 7.5 Hz, 2H), 6.82 (d, *J* = 7.7 Hz, 2H), 2.16 (s, 3H). ^{13}C NMR (101 MHz, DMSO-d_6) δ 190.68, 150.23, 143.50, 136.24, 134.54, 134.02, 133.60, 130.06, 129.98, 129.84, 129.33, 126.24, 125.54, 124.59, 121.32, 119.71, 116.49, 98.22, 79.66, 20.80. HRMS-ESI (m/z): calcd for $C_{23}H_{17}BrN_2O$, $[M+H]^+$: 417.0597; found, 417.0588.

(*E*)-2-(1-bromo-1*H*-indol-3-yl)-2-((4-chlorophenyl)imino)-1-phenylethan-1-one (3p):

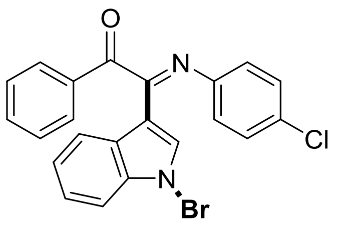

Yellow oil. Yield 77 mg (59%) at 0.3 mmol scale. ^{1}H NMR (400 MHz, DMSO-d_6) δ 8.48 (d, *J* = 8.2 Hz, 1H), 7.87 (d, *J* = 7.7 Hz, 2H), 7.72 (d, *J* = 7.6 Hz, 2H), 7.54 (t, *J* = 7.6 Hz, 2H), 7.43 – 7.32 (m, 3H), 7.26 (d, *J* = 8.9 Hz, 2H), 6.92 (d, *J* = 8.0 Hz, 2H), 6.81 (d, *J* = 3.4 Hz, 1H). ^{13}C NMR (101 MHz, DMSO-d_6) δ 190.50, 151.77, 145.51, 136.37, 135.07, 133.67, 130.85, 130.12, 129.96, 129.25, 128.81, 126.64, 125.00, 124.07, 123.27, 121.76, 116.48, 109.32. HRMS-ESI (m/z): calcd for $C_{22}H_{14}BrClN_2O$, $[M+H]^+$: 437.0051; found, 437.0040.

(*E*)-2-(1-bromo-5-methyl-1*H*-indol-3-yl)-1-phenyl-2-(phenylimino)ethan-1-one (3q)：

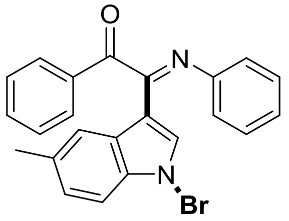

Yellow solid. M.P. 158 – 159 ℃. Yield 97 mg (78%) at 0.3 mmol scale. ^{1}H NMR (400 MHz, DMSO-d_6) δ 8.33 (d, *J* = 8.6 Hz, 1H), 7.84 (d, *J* = 7.7 Hz, 2H), 7.68 (t, *J* = 6.0 Hz, 2H), 7.50 (t, *J* = 7.7 Hz, 2H), 7.37 (s, 1H), 7.29 (d, *J* = 8.5 Hz, 1H), 7.18 (t, *J* = 7.7 Hz, 2H), 6.96 (t, *J* = 7.5 Hz, 1H), 6.88 (d, *J* = 7.7 Hz, 2H), 2.47 (s, 3H). ^{13}C NMR (101 MHz, DMSO-d_6) δ 190.43, 150.37, 146.19, 136.20, 134.08, 133.68, 132.83, 130.04, 129.94, 129.55, 129.31, 127.57, 125.55, 124.86, 121.40, 119.40, 116.26, 98.15, 21.41. HRMS-ESI (m/z): calcd for $C_{23}H_{17}BrN_2O$, $[M+H]^+$: 417.0597; found, 417.0593.

(*E*)-2-(1-bromo-6-chloro-1*H*-indol-3-yl)-1-phenyl-2-(phenylimino)ethan-1-one (3r)：

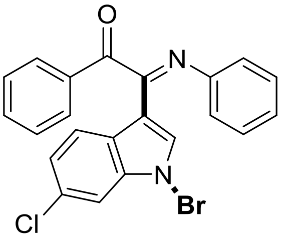

Yellow oil. Yield 86 mg (66%) at 0.3 mmol scale. ^{1}H NMR (400 MHz, DMSO-d_6) δ 8.65 (t, *J* = 2.0 Hz, 1H), 7.88 (d, *J* = 6.8 Hz, 2H), 7.78 (d, *J* = 1.9 Hz, 1H), 7.70 (t, *J* = 7.5 Hz, 1H), 7.62 (dd, *J* = 8.5, 1.9 Hz, 1H), 7.55 – 7.49 (m, 3H), 7.21 (t, *J* = 7.8 Hz, 2H), 7.00 (t, *J* = 7.5 Hz, 1H), 6.92 (d, *J* = 7.6 Hz, 2H). ^{13}C NMR (101 MHz, DMSO-d_6) δ 189.91, 150.52, 145.86, 136.31, 134.75, 133.53, 130.86, 130.20, 129.92, 129.38, 128.29, 126.76, 125.14, 125.06, 121.34, 121.15, 116.61, 97.94. HRMS-ESI (m/z): calcd for $C_{22}H_{14}BrClN_2O$, $[M+H]^+$: 437.0051; found, 437.0036.

6 GC-MS of intermediate C and *N*-formanilide.

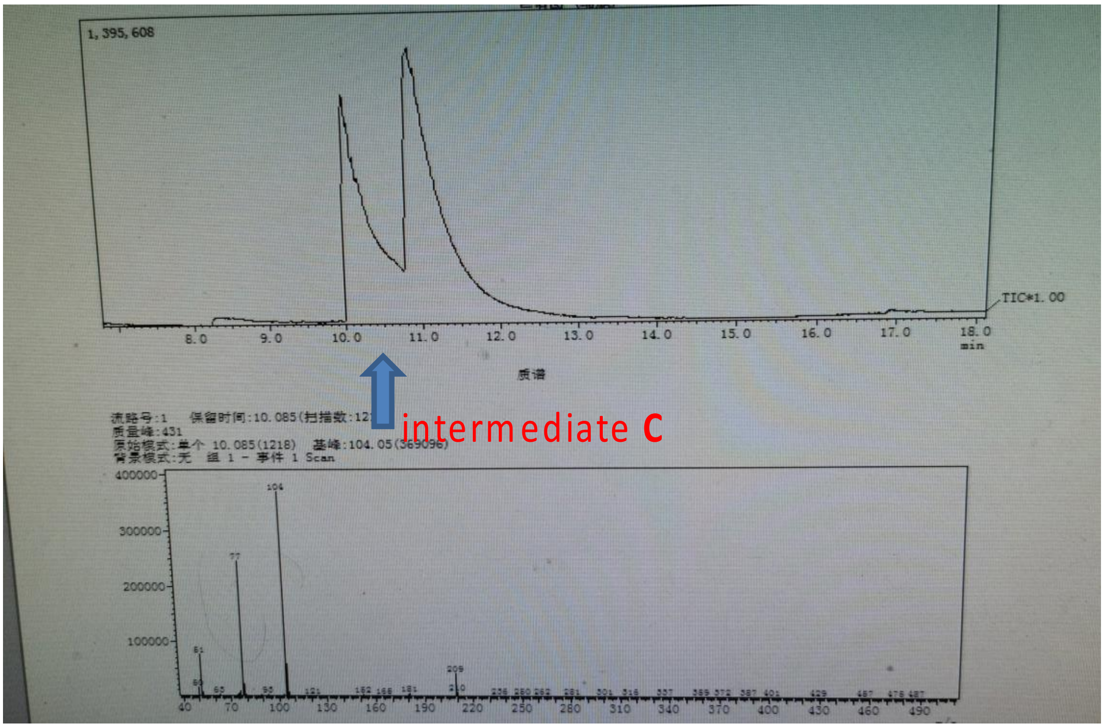

Fig. 1 GC-MS of intermediate **C**

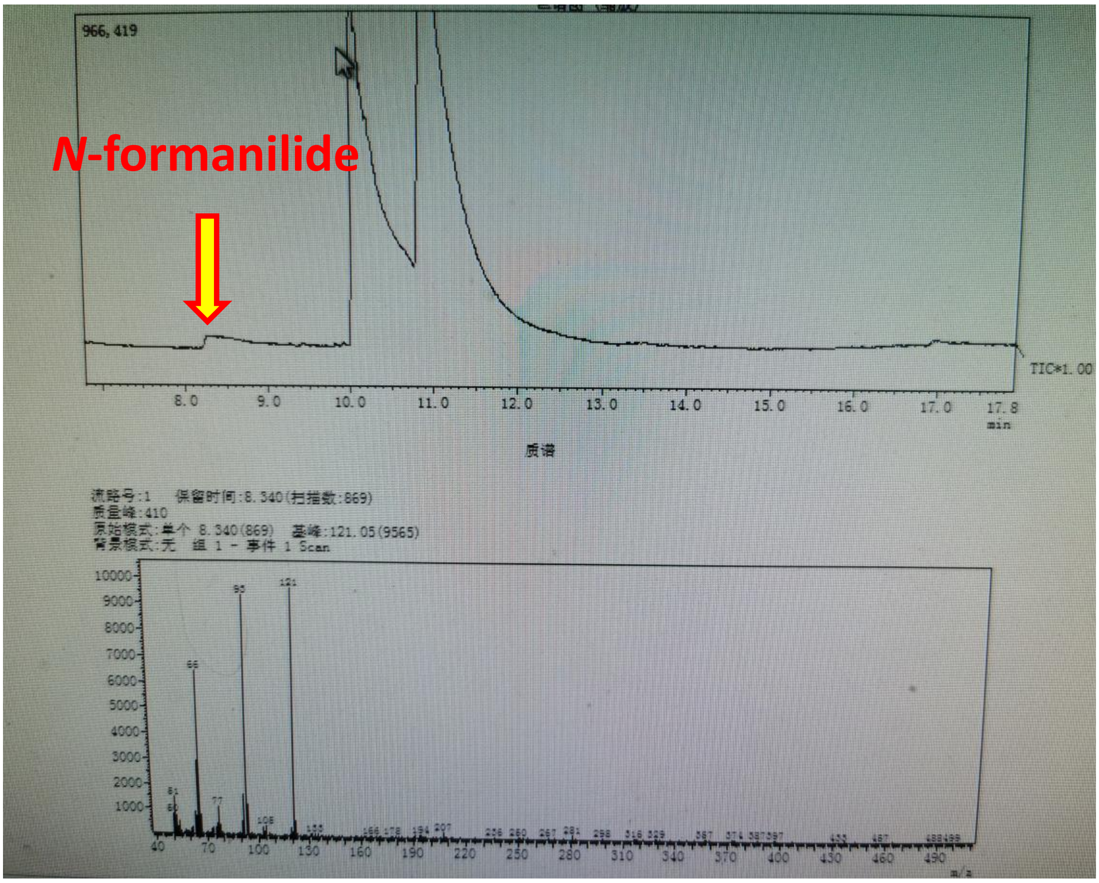

Fig. 2 GC-MS of *N*-formanilide

7. References

[1] M. H. Zhu, D. Chen, S. Zeng, C. H. Xing, W. Deng, J. N. Xiang, R.-J. Wang, Synthesis of (*Z*)-nitroalkene derivatives through oxidative dehydrogenation coupling of α-aminocarbonyl compounds with nitromethane by copper catalysis. *Tetrahedron Lett.*, **2018**, *59*, 3214-3219.

[2] C. Chen, M. H. Zhu, L. H. Jiang, Z. B. Zeng, N. N. Yi, J. N. Xiang, Copper-catalyzed oxidative cross-coupling of α-aminocarbonyl compounds with primary amines toward 2-oxo-acetamidines. *Org. Biomol. Chem.*, **2017**, *15*, 8134-8139.

8. The ^{1}H NMR, ^{13}C NMR and HRMS spectra for the compounds

(*E*)-2-(1-bromo-1*H*-indol-3-yl)-1-phenyl-2-(phenylimino)ethan-1-one (3a)

^{1}H NMR (400 MHz, DMSO-d_6)

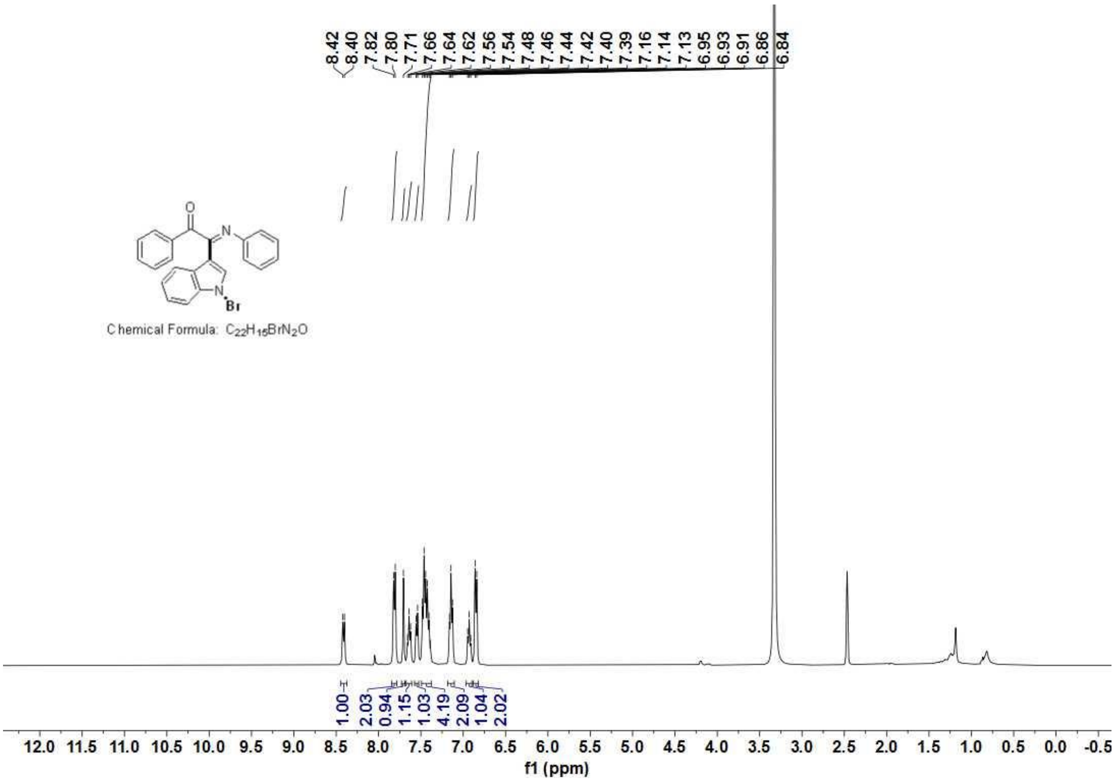

^{13}C NMR (101 MHz, DMSO-d_6)

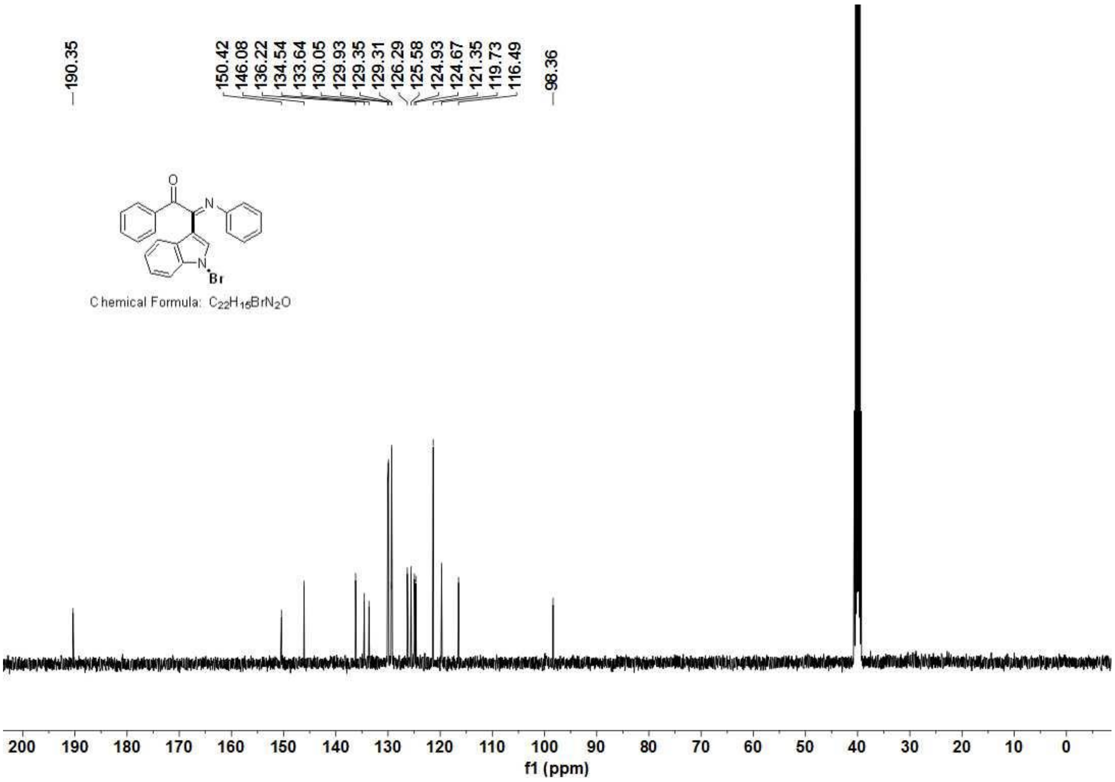

HRMS spectra

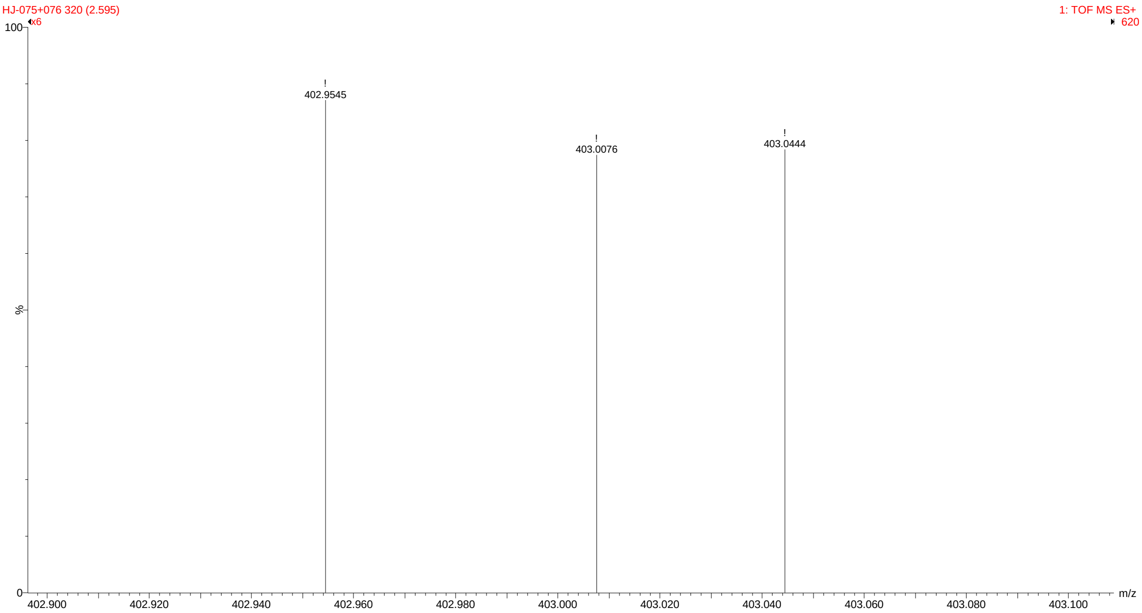

(*E*)-2-(1-bromo-1*H*-indol-3-yl)-2-(phenylimino)-1-(*o*-tolyl)ethan-1-one (3b)

^{1}H NMR (400 MHz, DMSO-d_6)

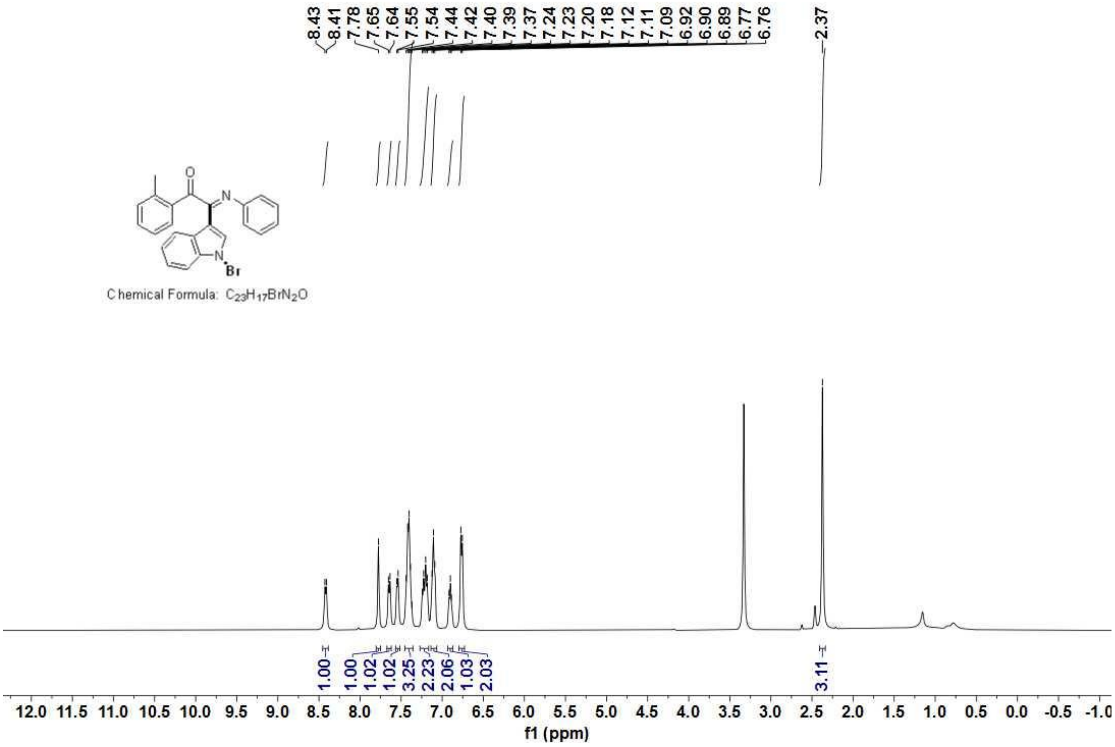

^{13}C NMR (101 MHz, DMSO-d_6)

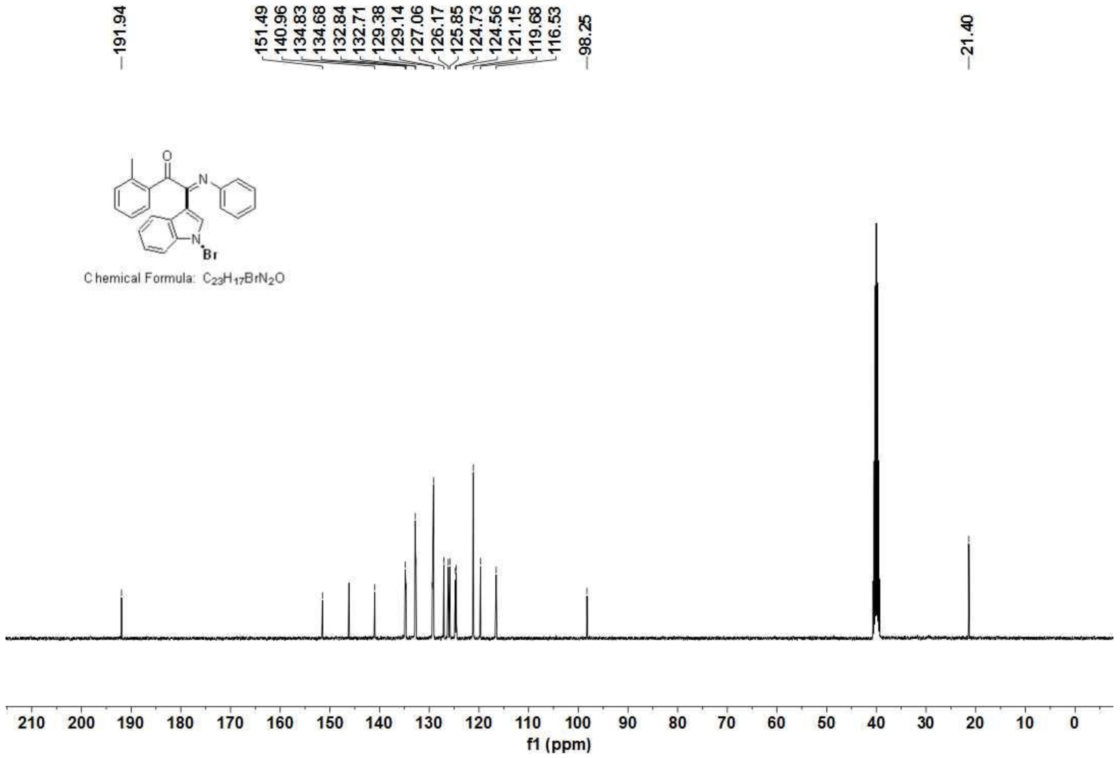

HRMS spectra

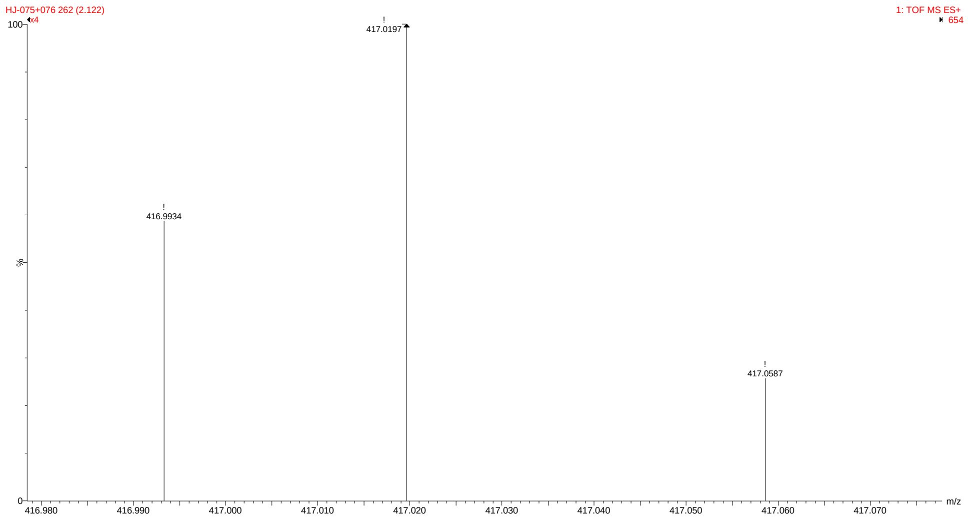

(*E*)-2-(1-bromo-1*H*-indol-3-yl)-2-(phenylimino)-1-(*p*-tolyl)ethan-1-one (3c)

^{1}H NMR (400 MHz, DMSO-d_6)

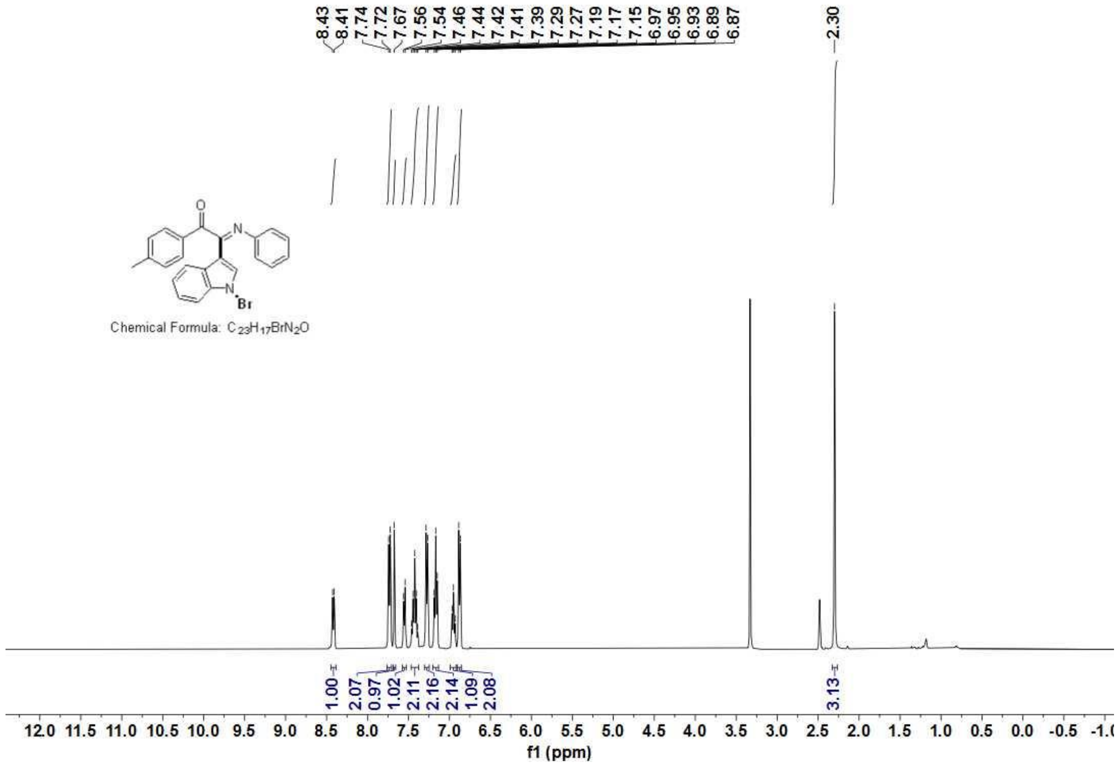

^{13}C NMR (101 MHz, DMSO-d_6)

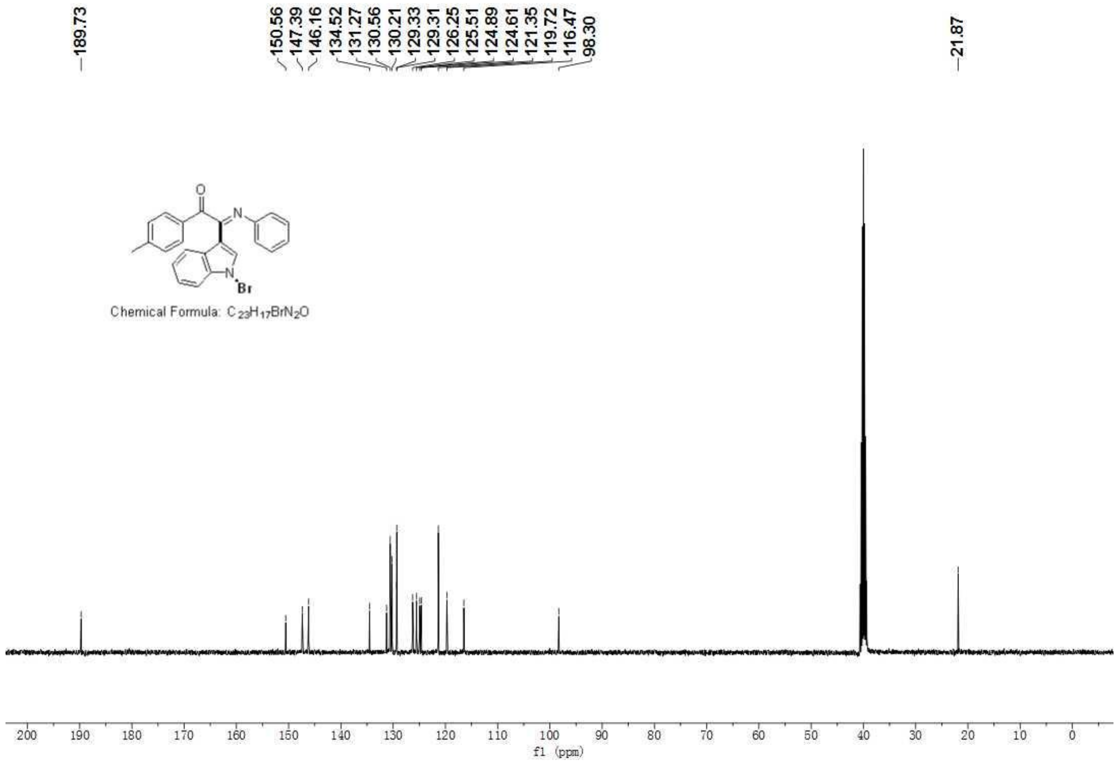

HRMS spectra

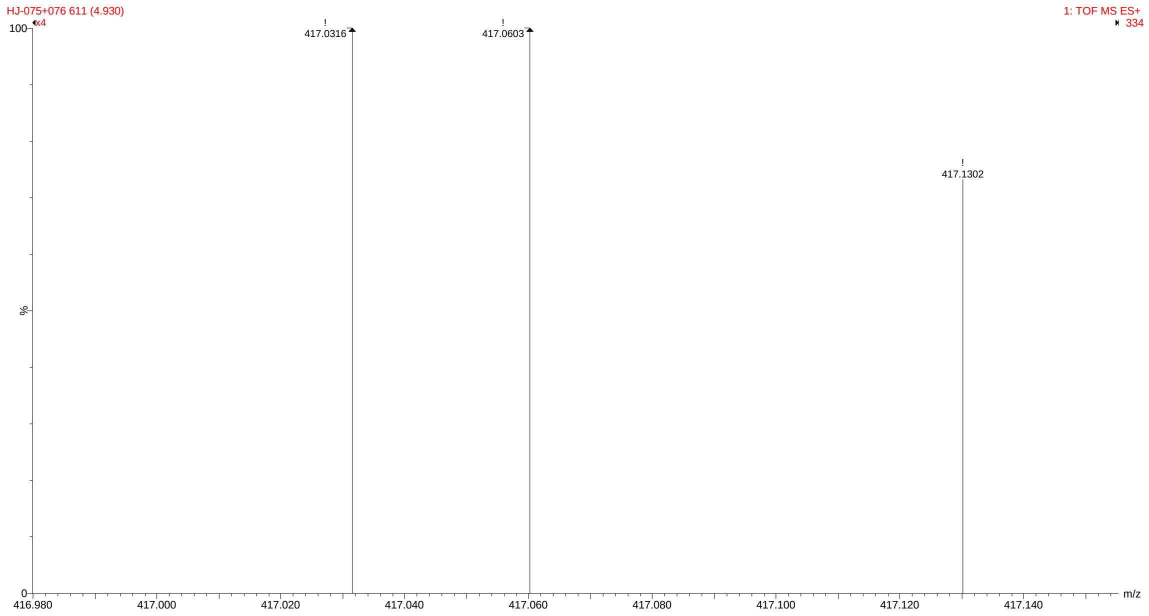

(*E*)-2-(1-bromo-1*H*-indol-3-yl)-1-(4-methoxyphenyl)-2-(phenylimino)ethan-1-one (3d)

^{1}H NMR (400 MHz, DMSO-d_6)

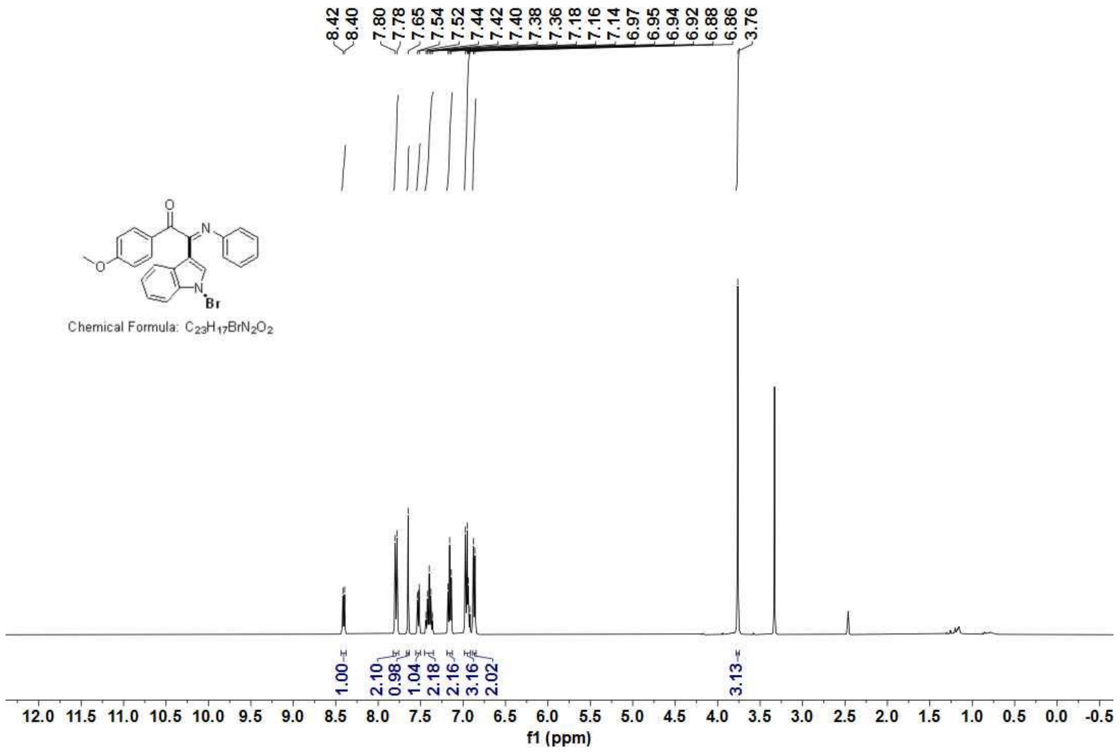

^{13}C NMR (101 MHz, DMSO-d_6)

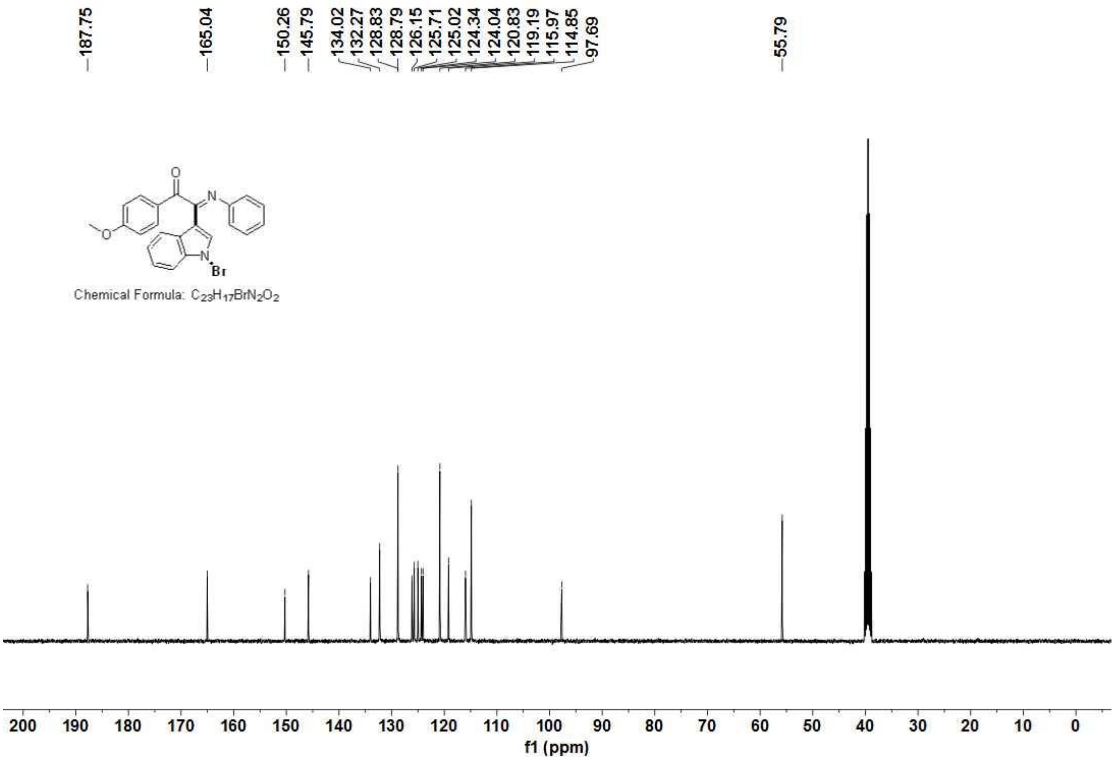

HRMS spectra

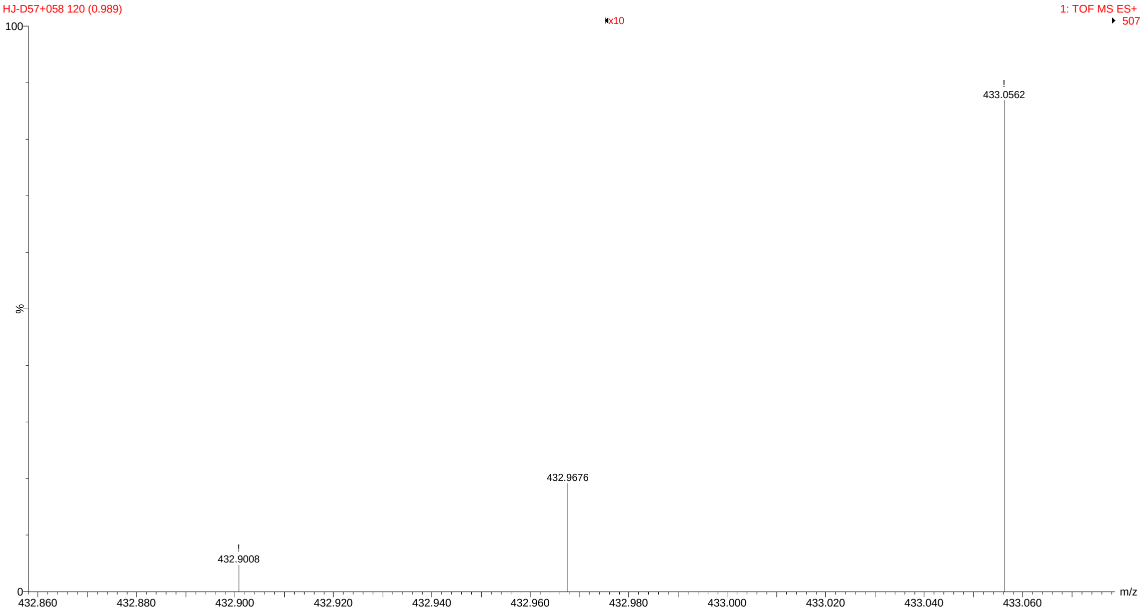

(*E*)-2-(1-bromo-1*H*-indol-3-yl)-1-(4-fluorophenyl)-2-(phenylimino)ethan-1-one (3e):

^{1}H NMR (400 MHz, DMSO-d_6)

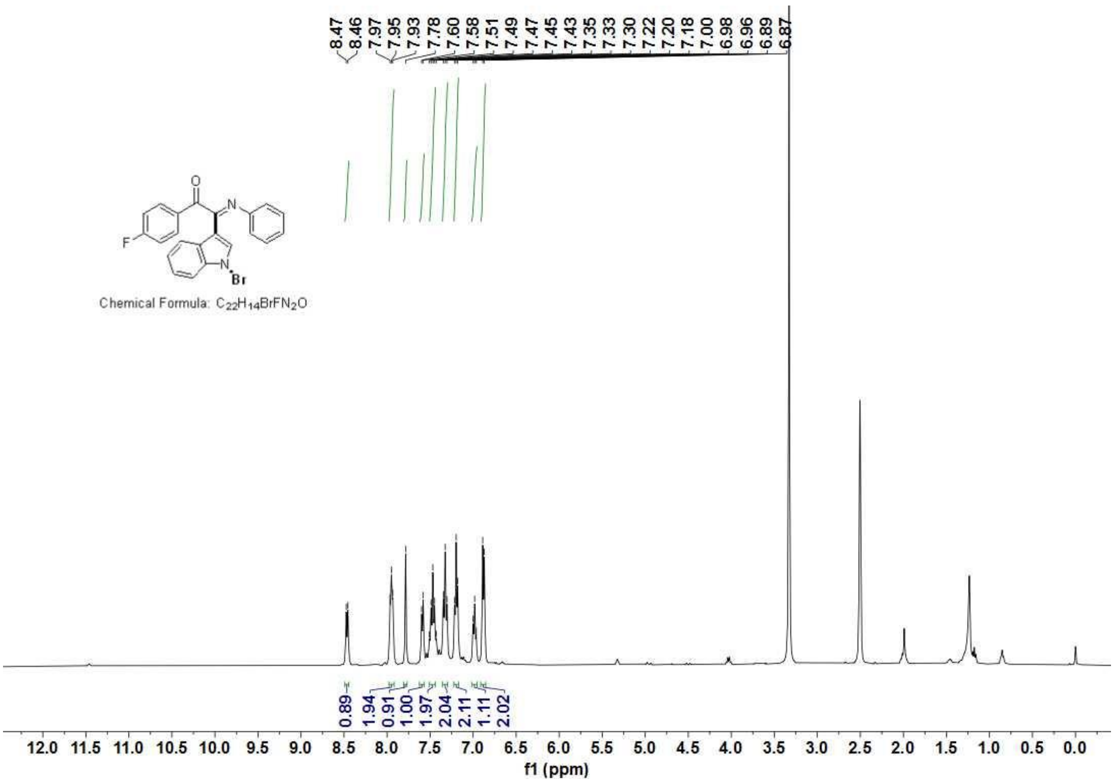

^{13}C NMR (101 MHz, DMSO-d_6)

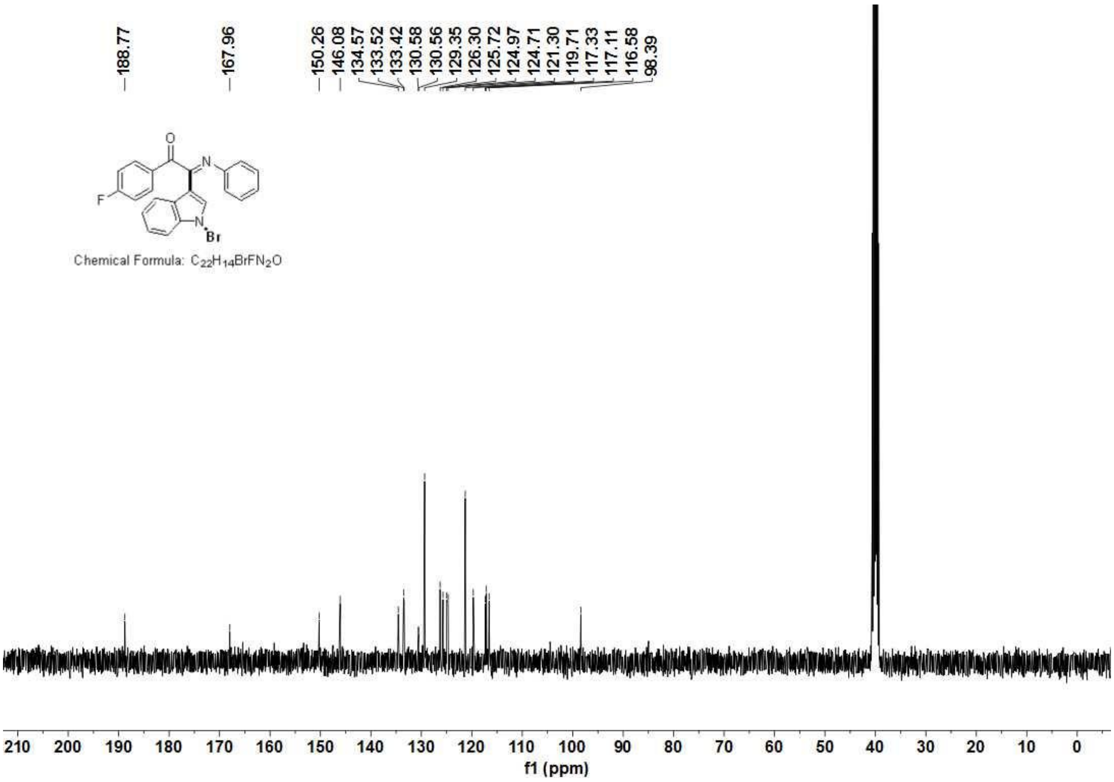

HRMS spectra

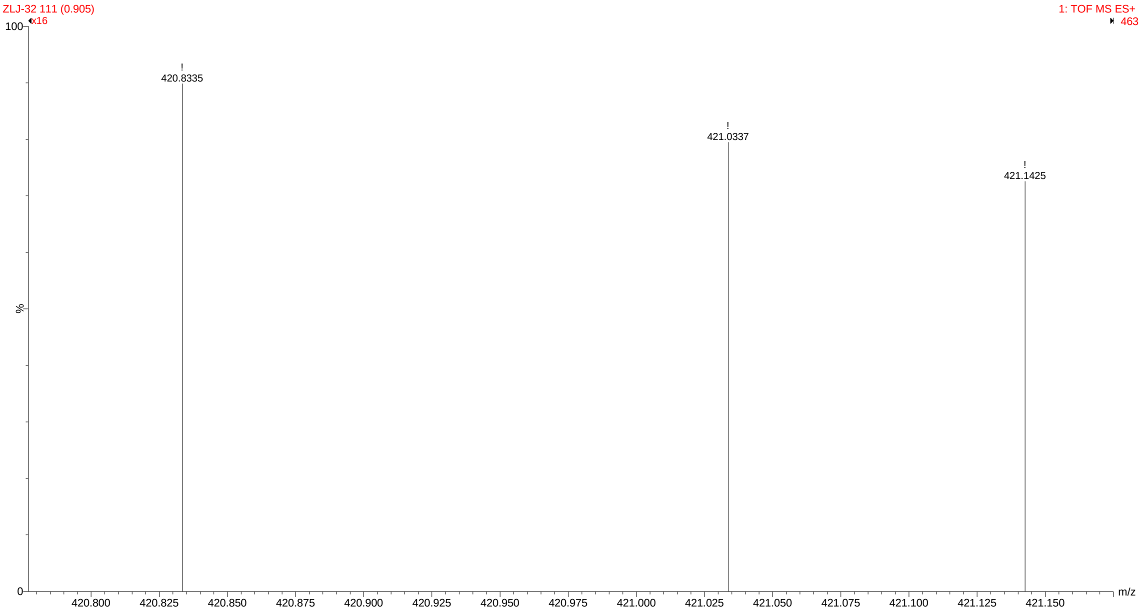

(*E*)-2-(1-bromo-1*H*-indol-3-yl)-1-(3-chlorophenyl)-2-(phenylimino)ethan-1-one (3f)：

^{1}H NMR (400 MHz, DMSO-d_6)

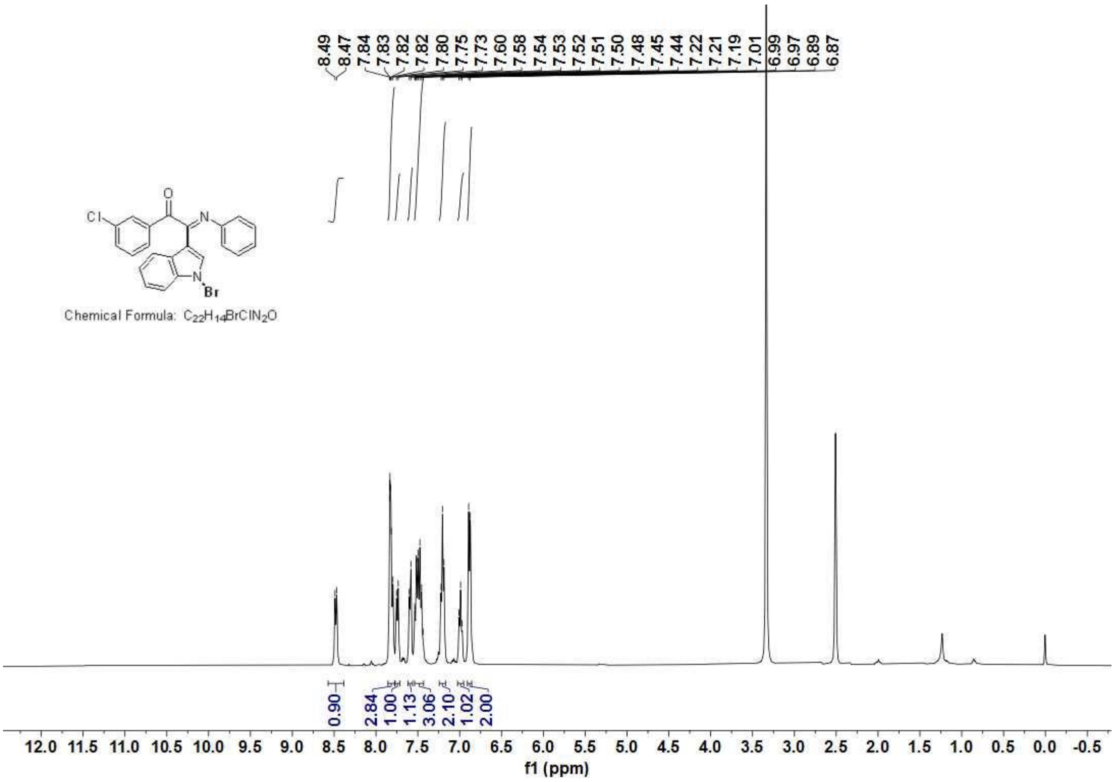

^{13}C NMR (101 MHz, DMSO-d_6)

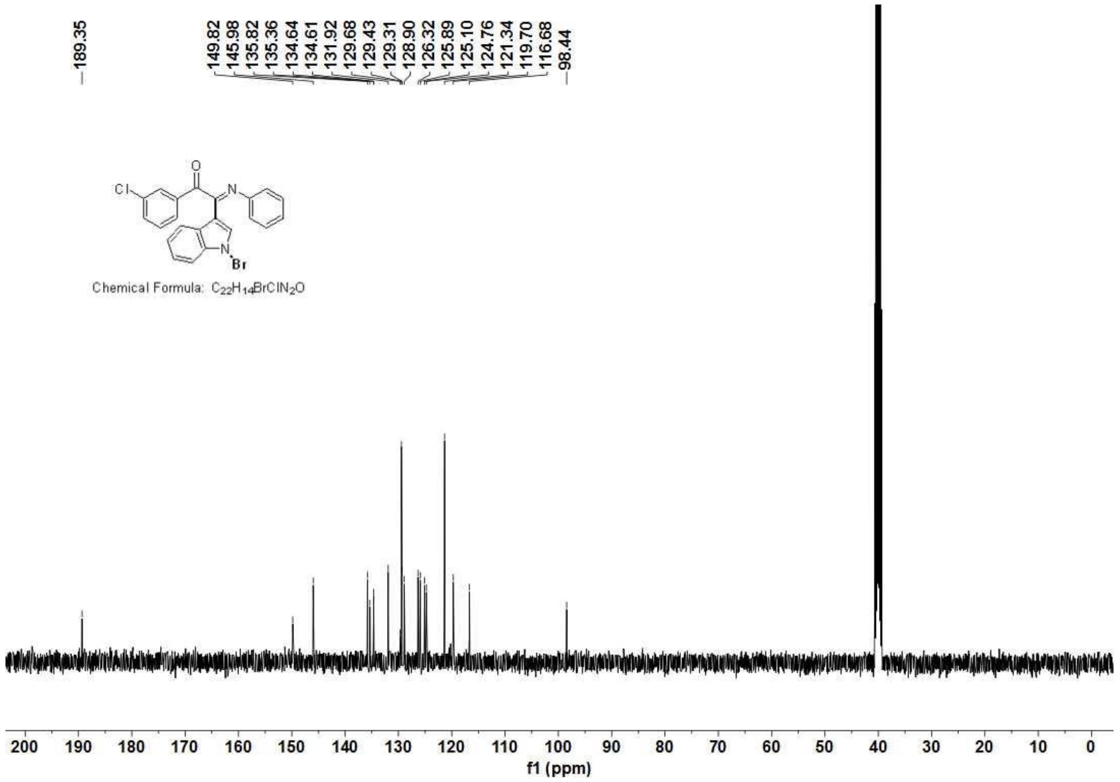

HRMS spectra

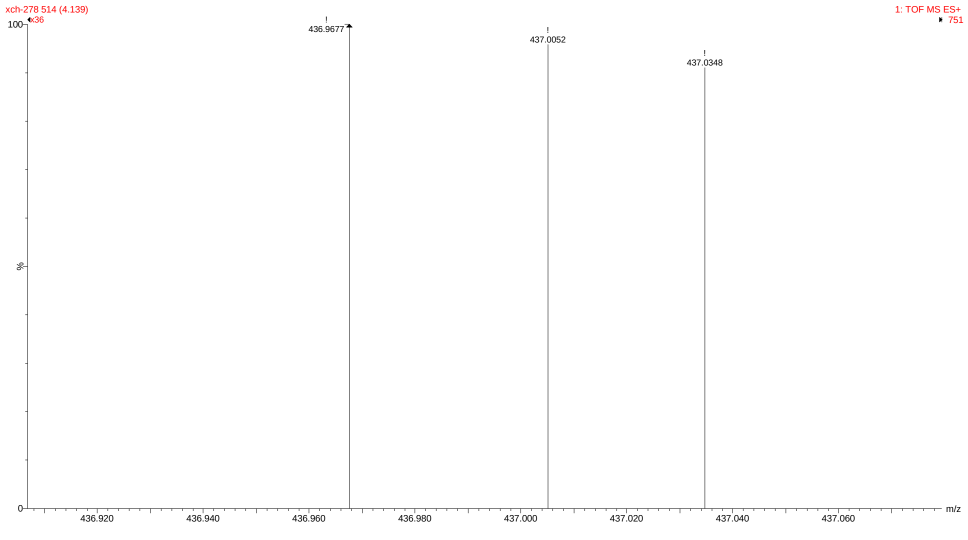

(*E*)-2-(1-bromo-1*H*-indol-3-yl)-1-(4-chlorophenyl)-2-(phenylimino)ethan-1-one (3g)

^{1}H NMR (400 MHz, DMSO-d_6)

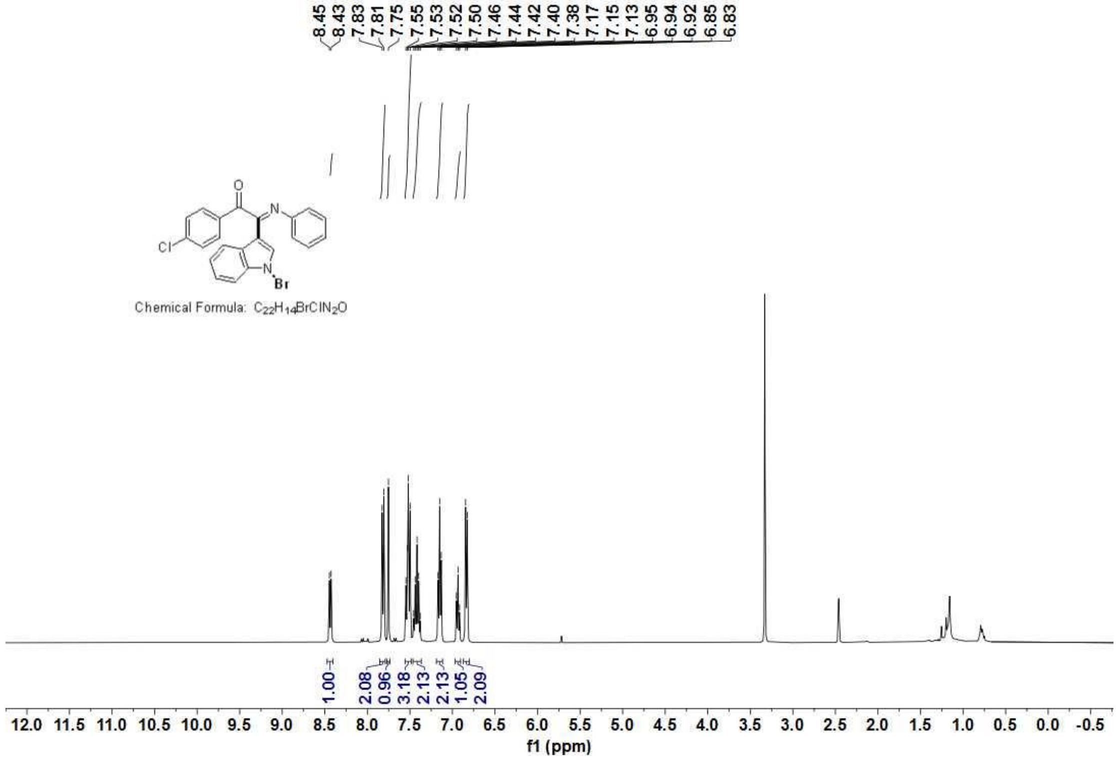

^{13}C NMR (101 MHz, DMSO-d_6)

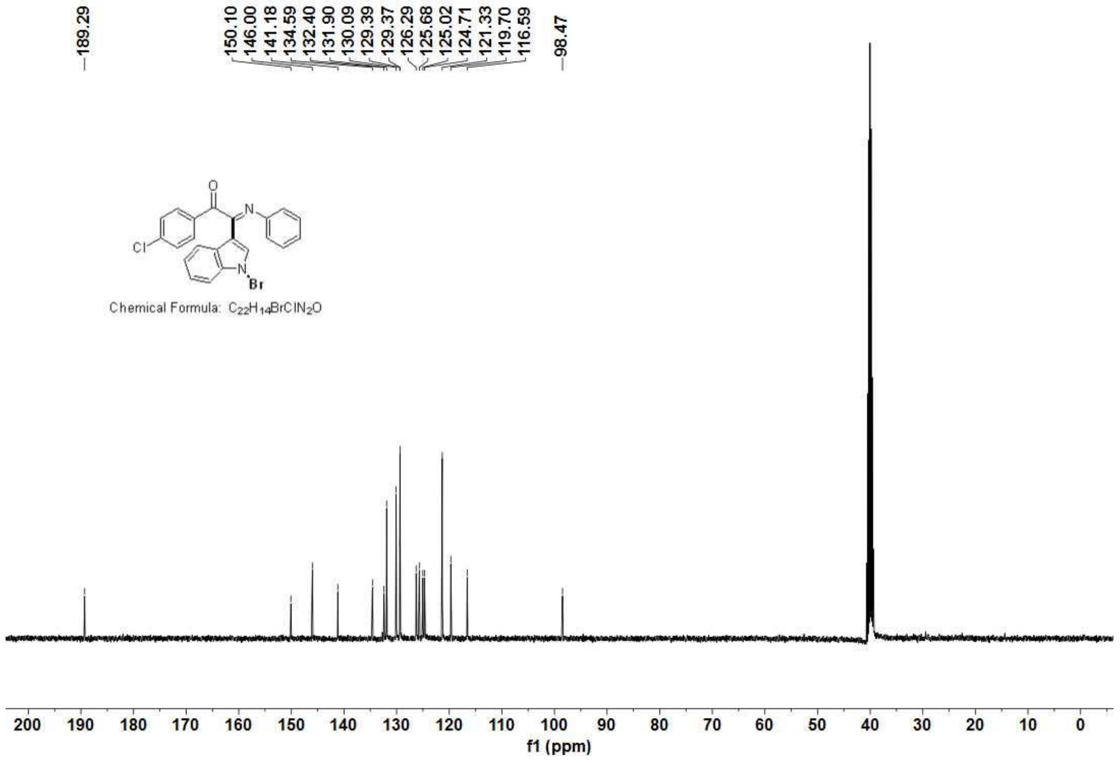

HRMS spectra

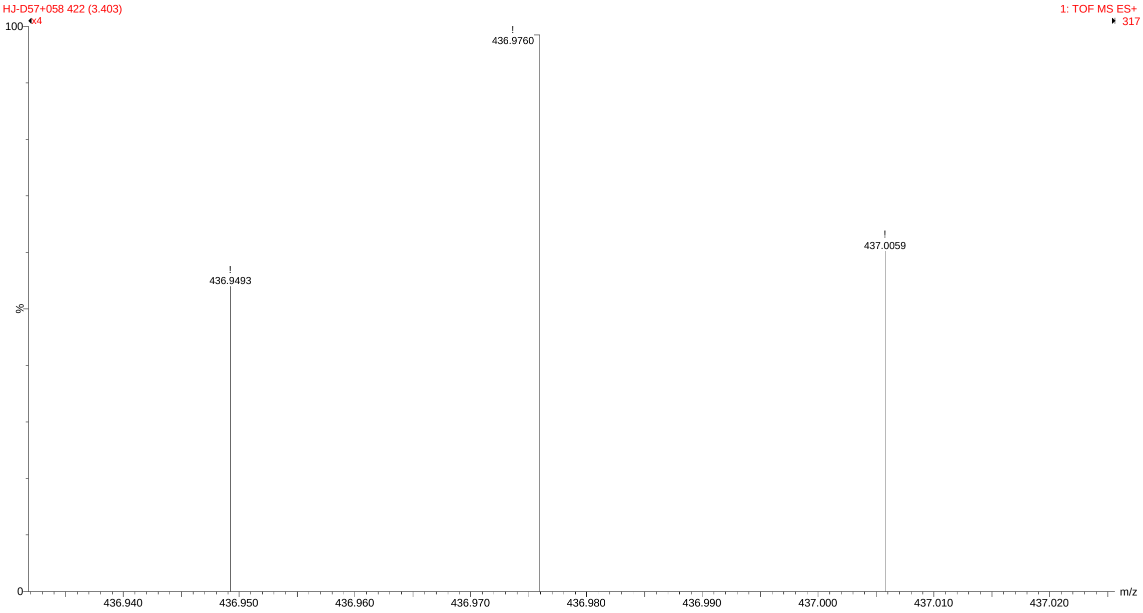

(*E*)-2-(1-bromo-1*H*-indol-3-yl)-1-(2-bromophenyl)-2-(phenylimino)ethan-1-one (3h):

^{1}H NMR (400 MHz, DMSO-d_6)

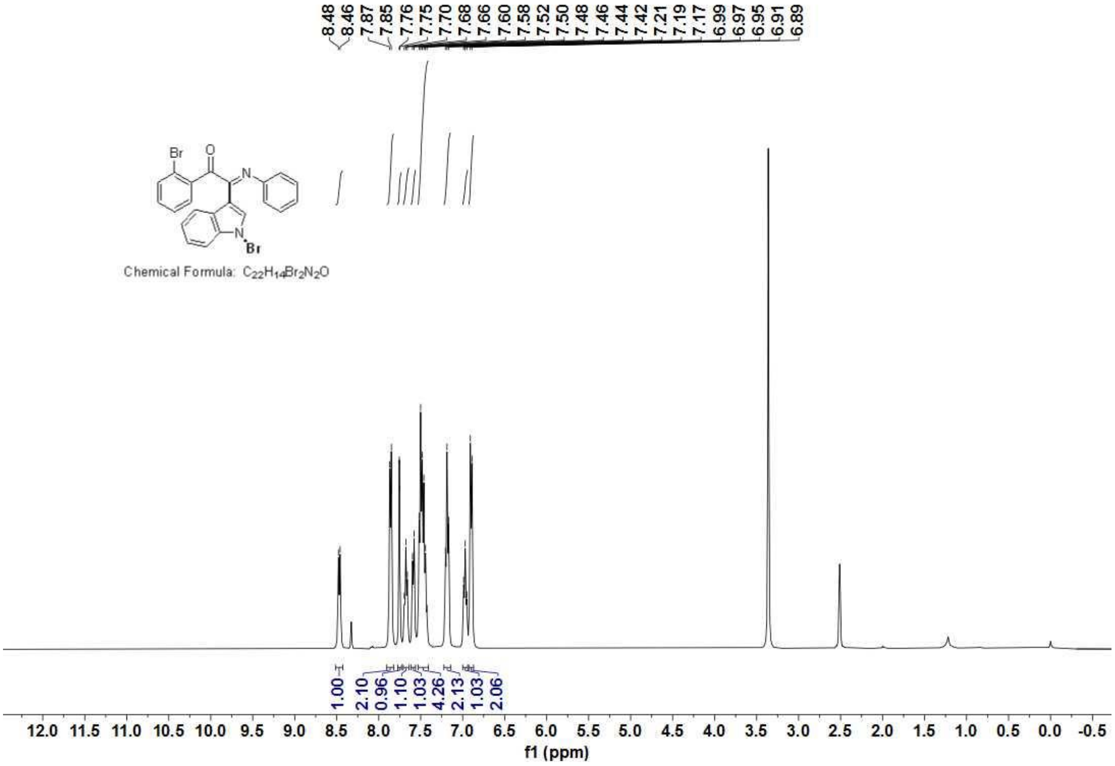

^{13}C NMR (101 MHz, DMSO-d_6)

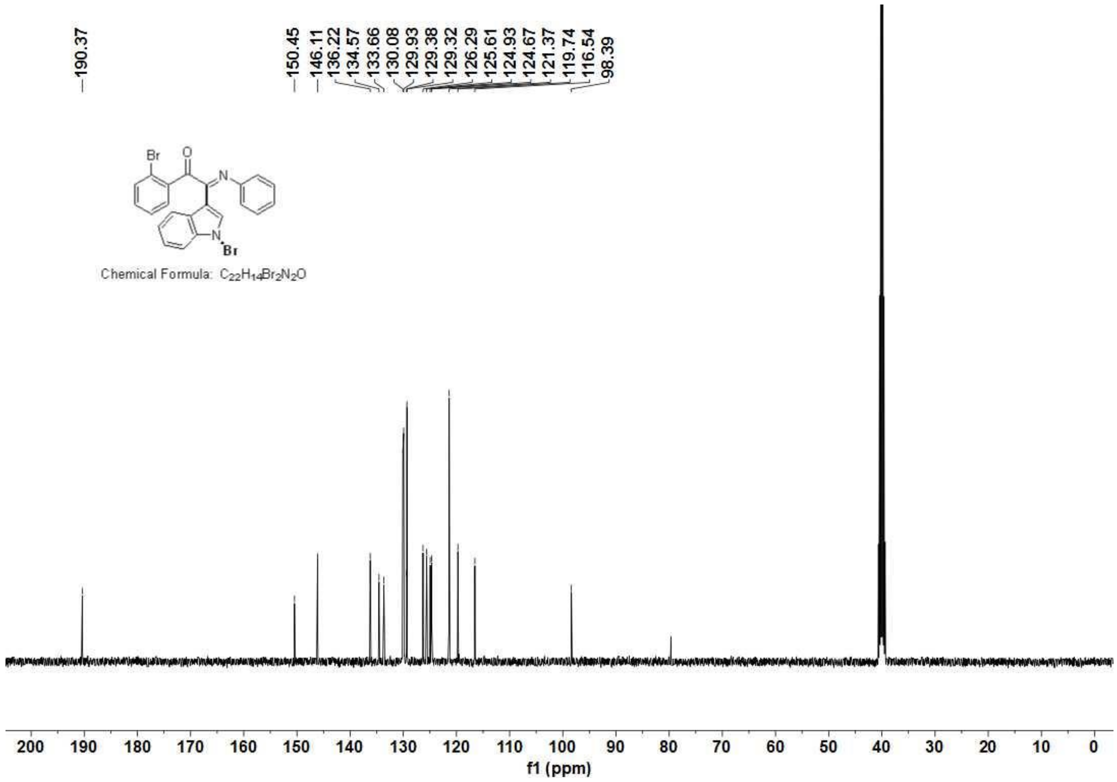

HRMS spectra

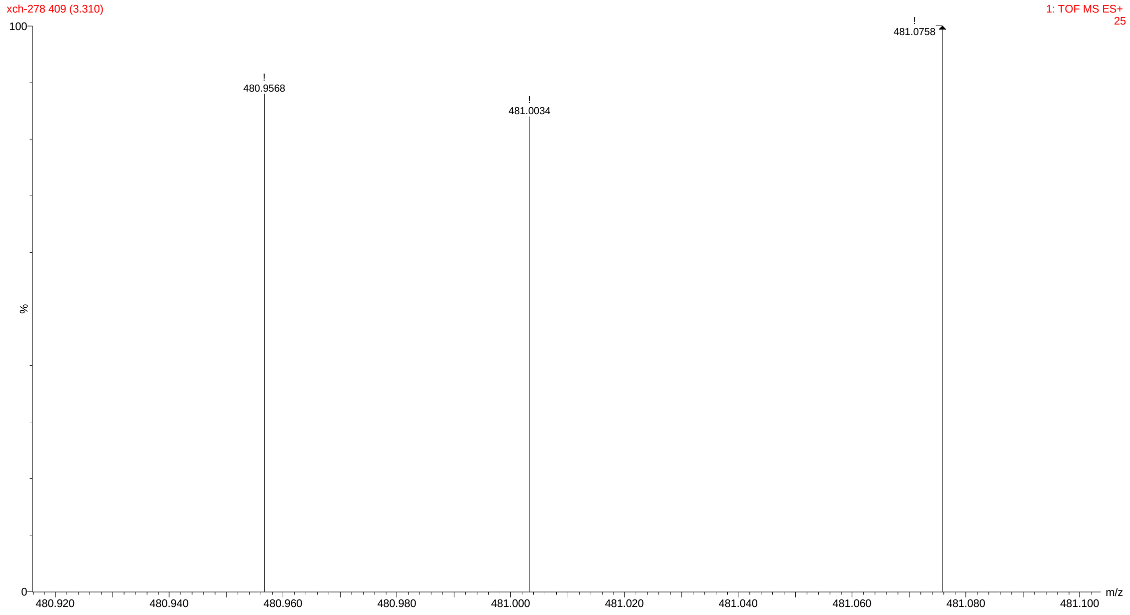

(*E*)-2-(1-bromo-1*H*-indol-3-yl)-1-(3-bromophenyl)-2-(phenylimino)ethan-1-one (3i):

^{1}H NMR (400 MHz, DMSO-d_6)

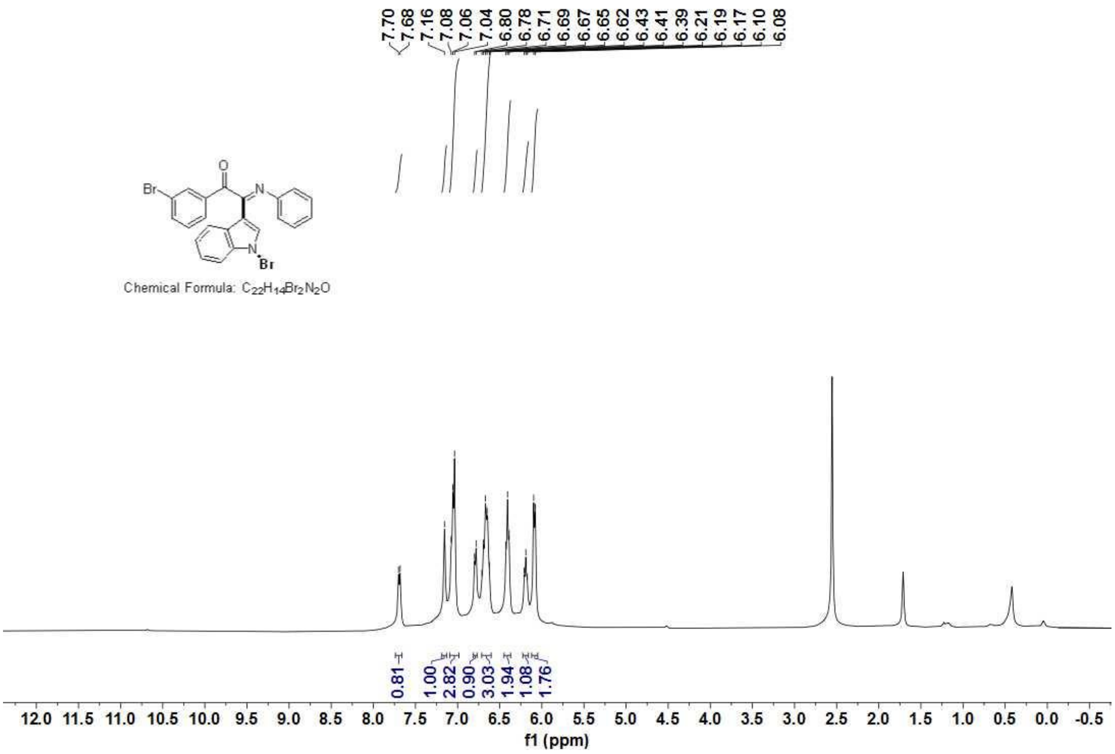

^{13}C NMR (101 MHz, DMSO-d_6)

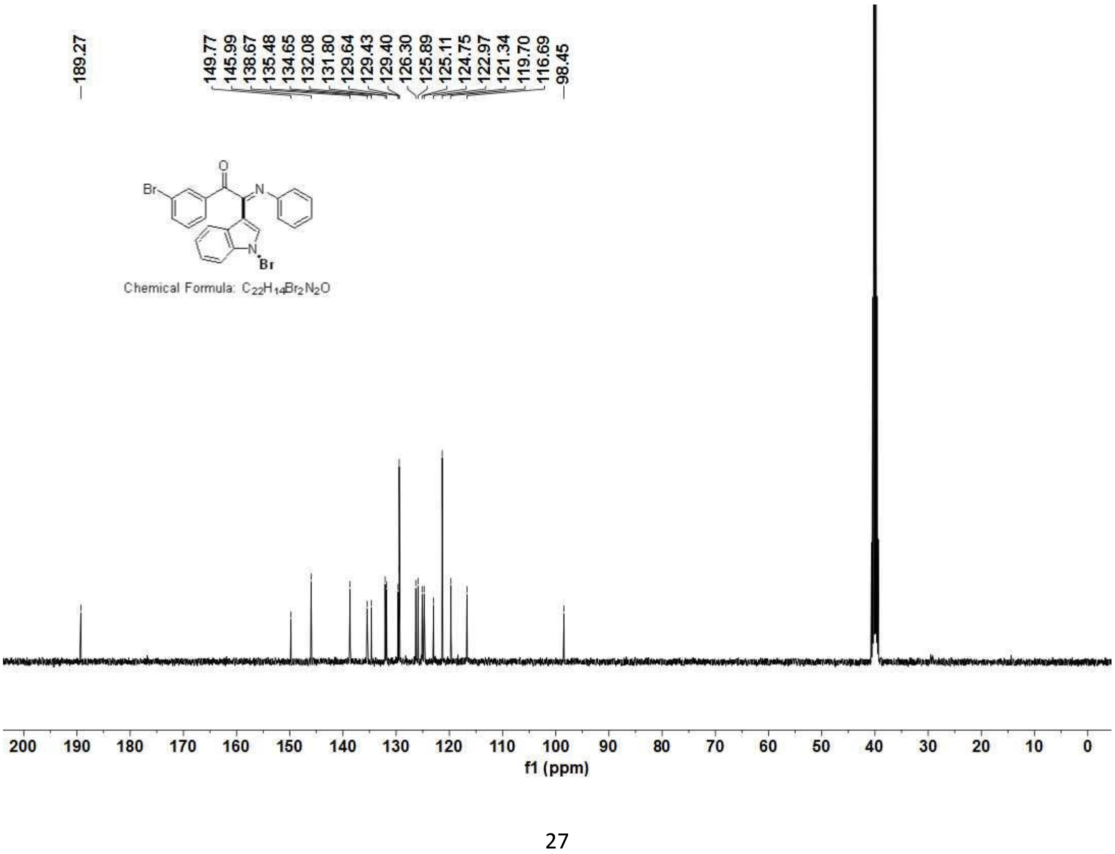

HRMS spectra

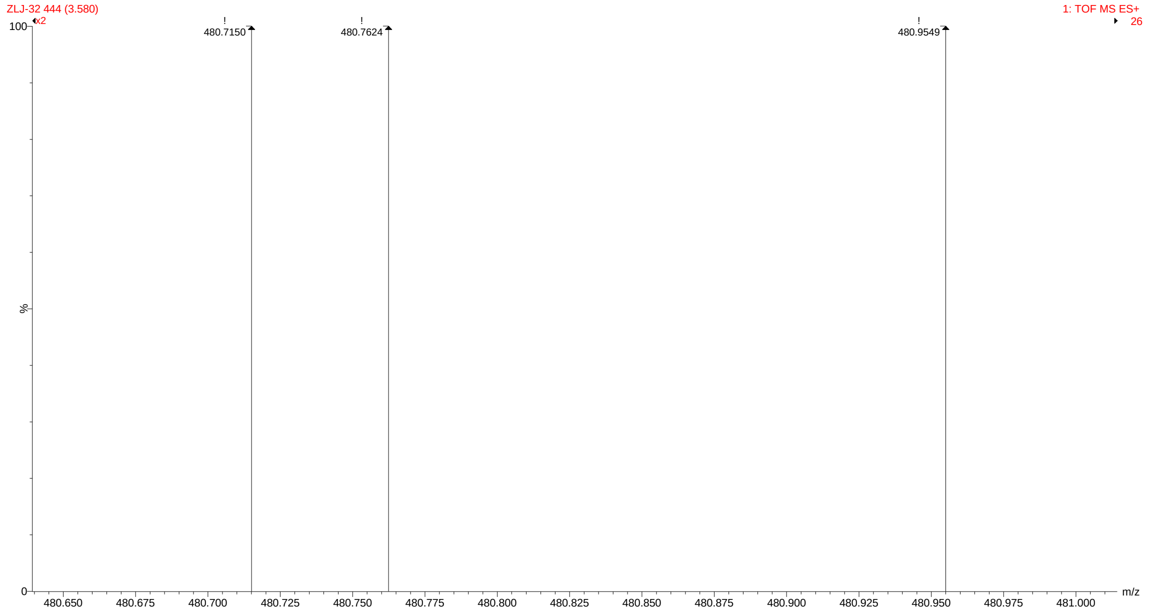

(*E*)-2-(1-bromo-1*H*-indol-3-yl)-1-(4-bromophenyl)-2-(phenylimino)ethan-1-one (3j)

^{1}H NMR (400 MHz, DMSO-d_6)

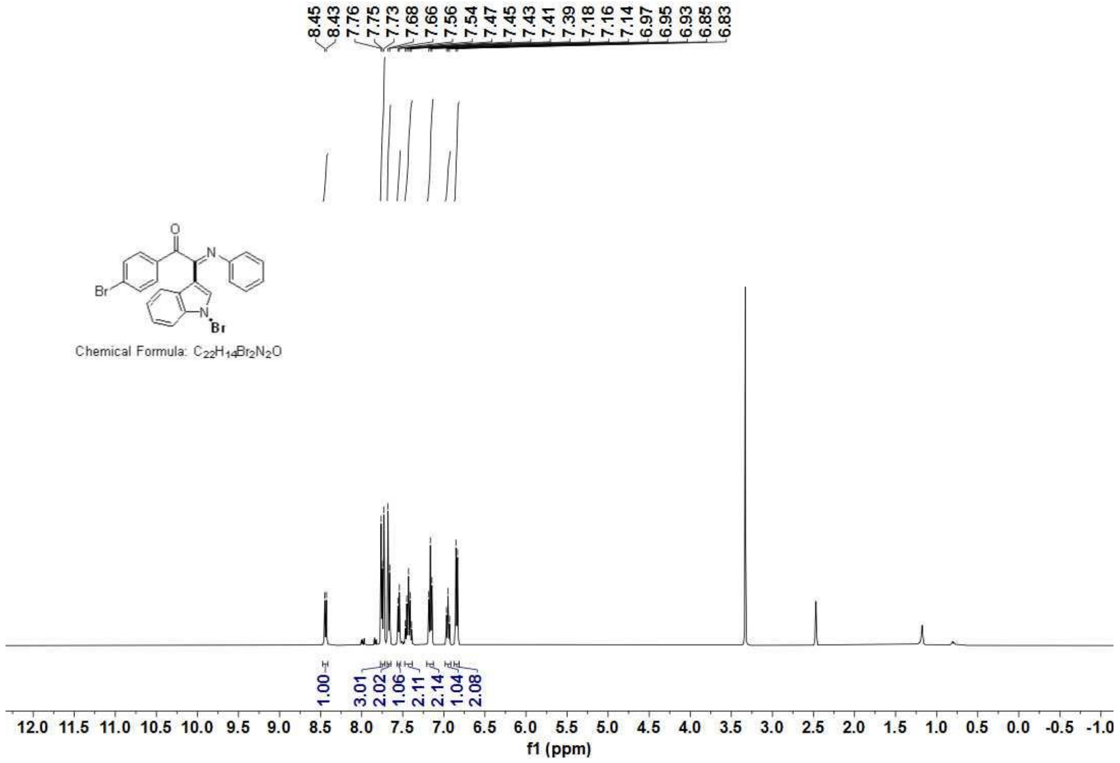

^{13}C NMR (101 MHz, DMSO-d_6)

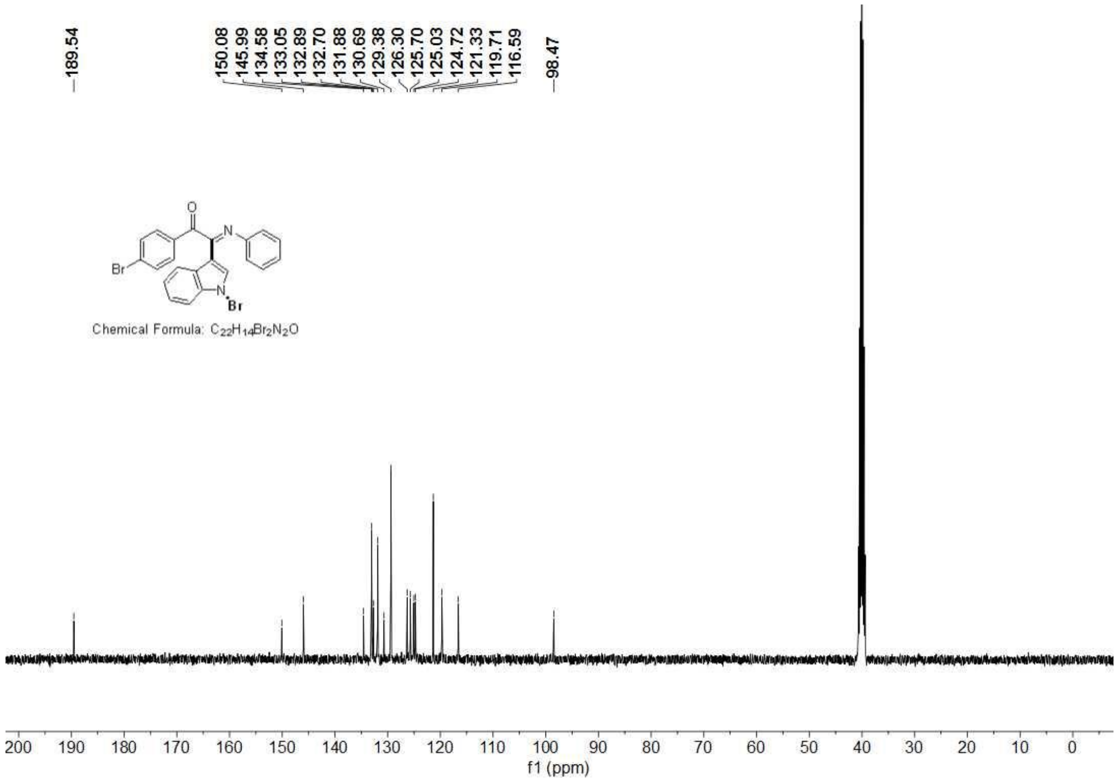

HRMS spectra

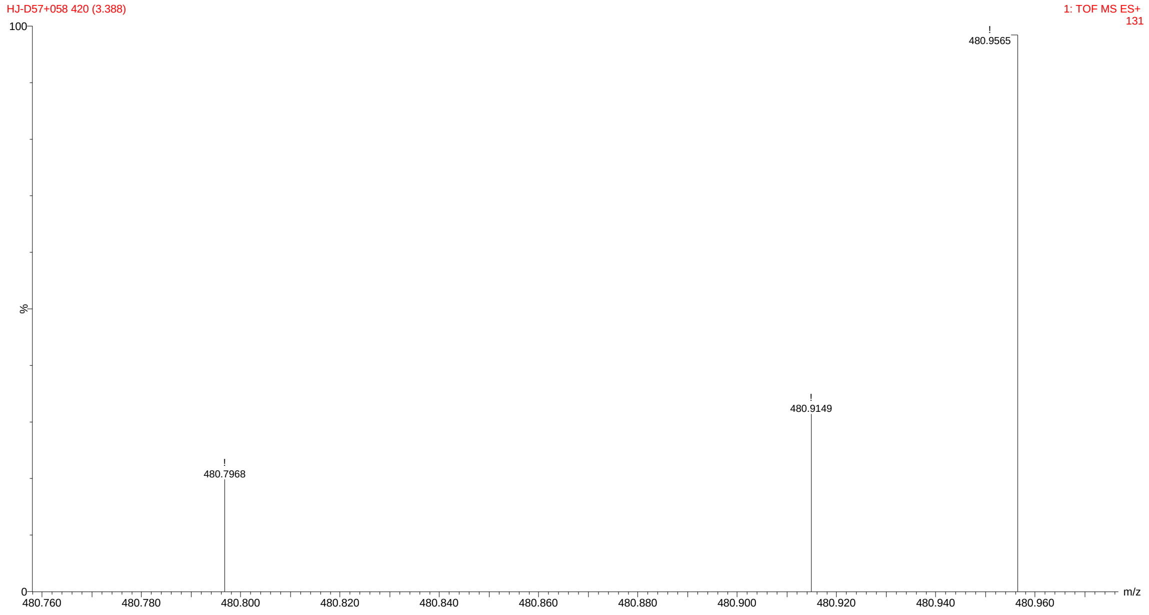

(*E*)-2-(1-bromo-1*H*-indol-3-yl)-1-(4-iodophenyl)-2-(phenylimino)ethan-1-one (3k)

^{1}H NMR (400 MHz, DMSO-d_6)

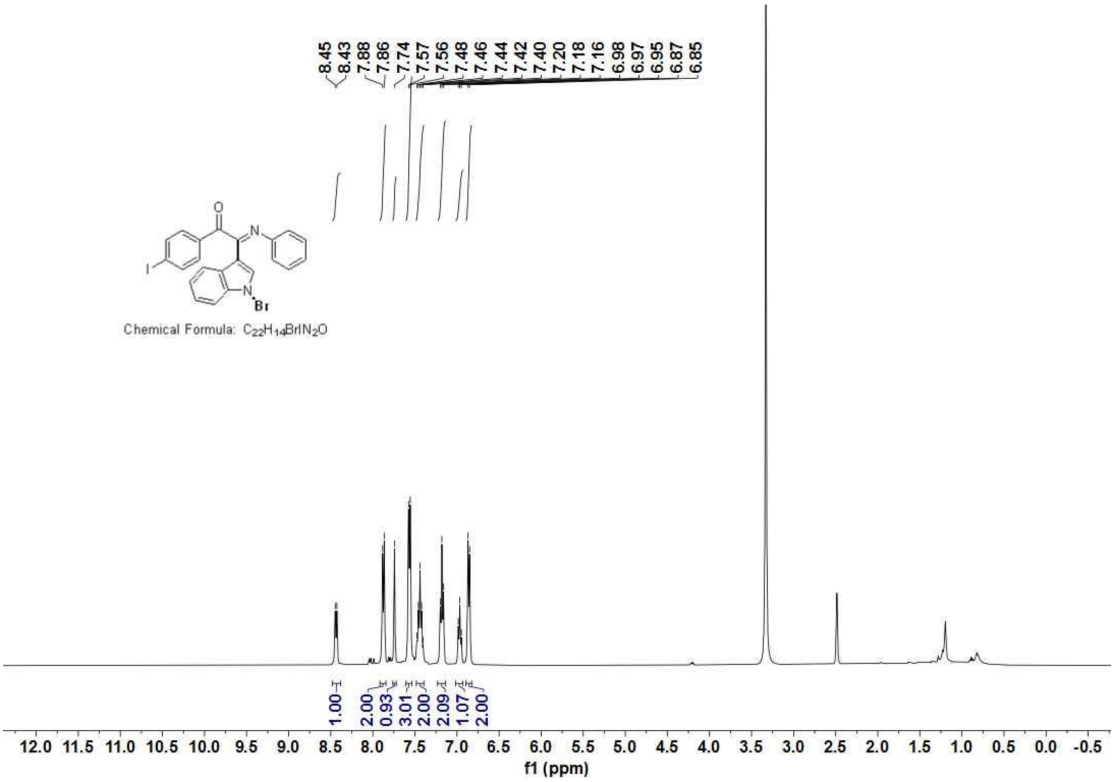

^{13}C NMR (101 MHz, DMSO-d_6)

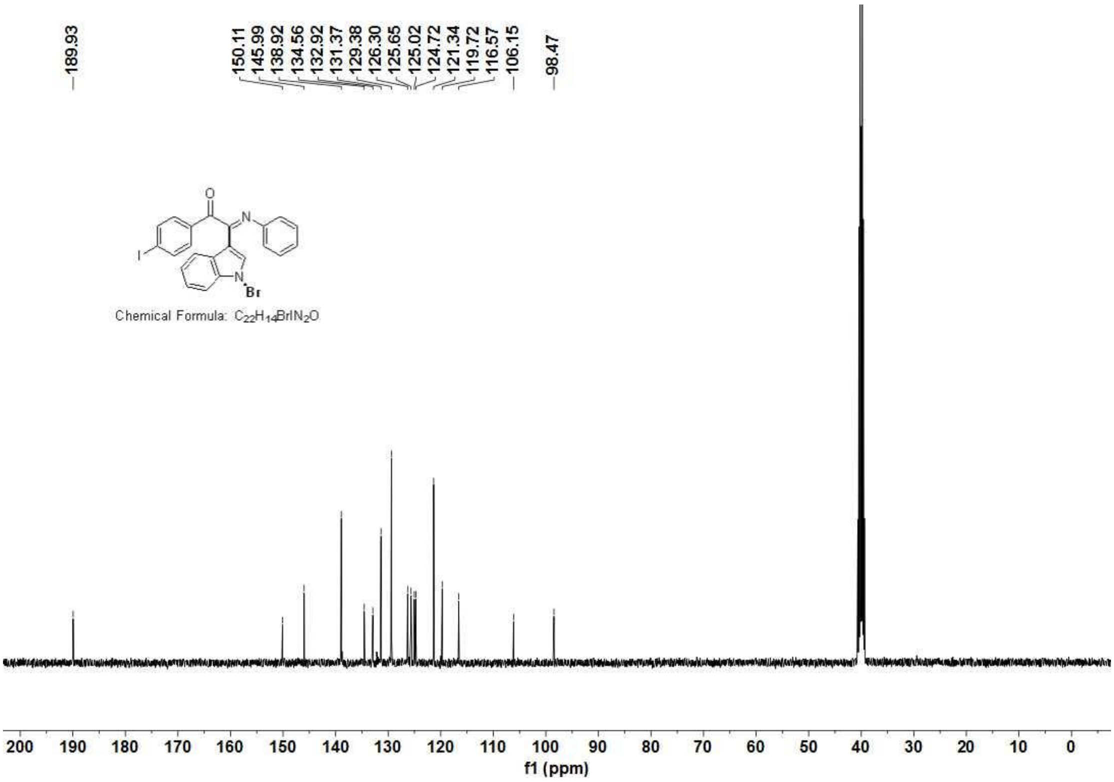

HRMS spectra

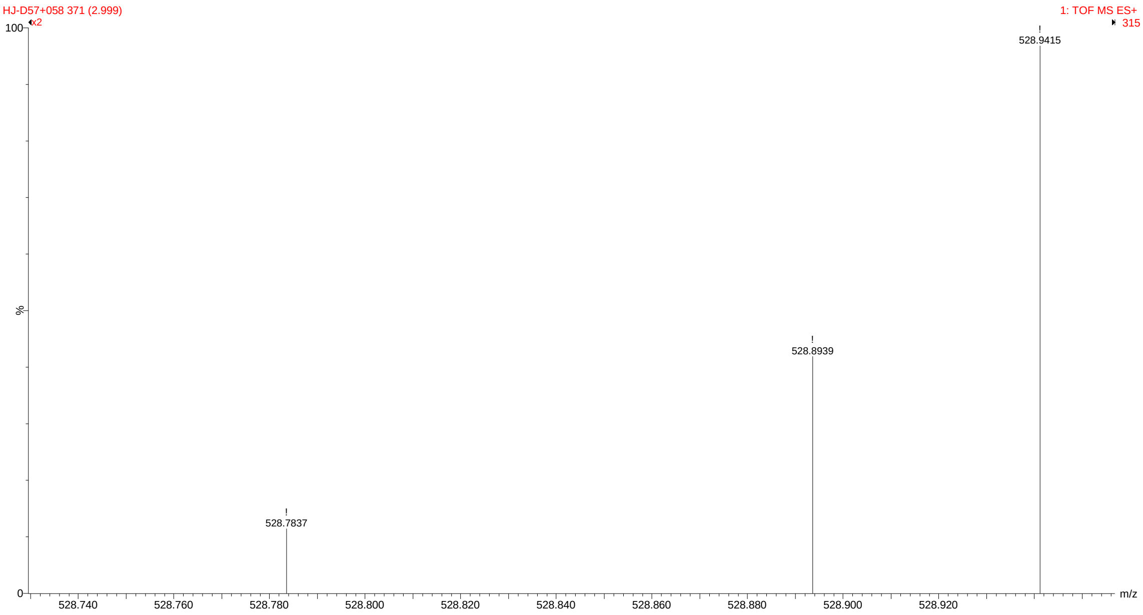

(*E*)-2-(1-bromo-1*H*-indol-3-yl)-2-(phenylimino)-1-(4(trifluoromethyl)pheny)ethan-1-one (3l):

^{1}H NMR (400 MHz, DMSO-d_6)

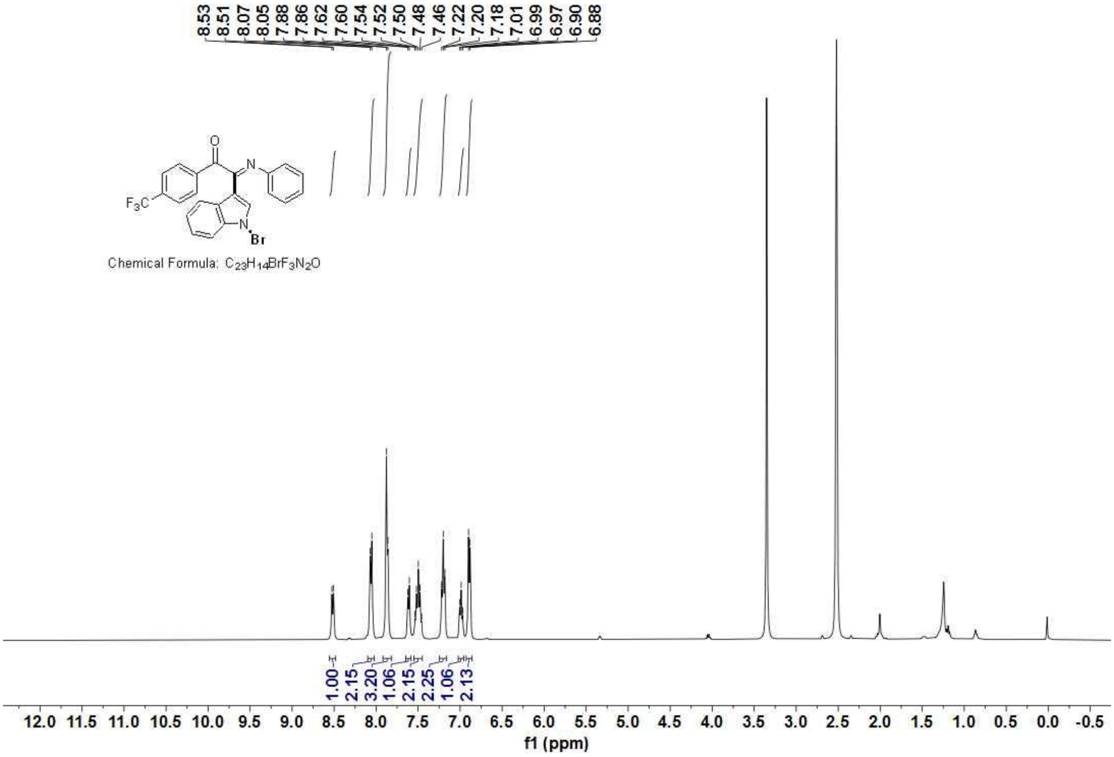

^{13}C NMR (101 MHz, DMSO-d_6)

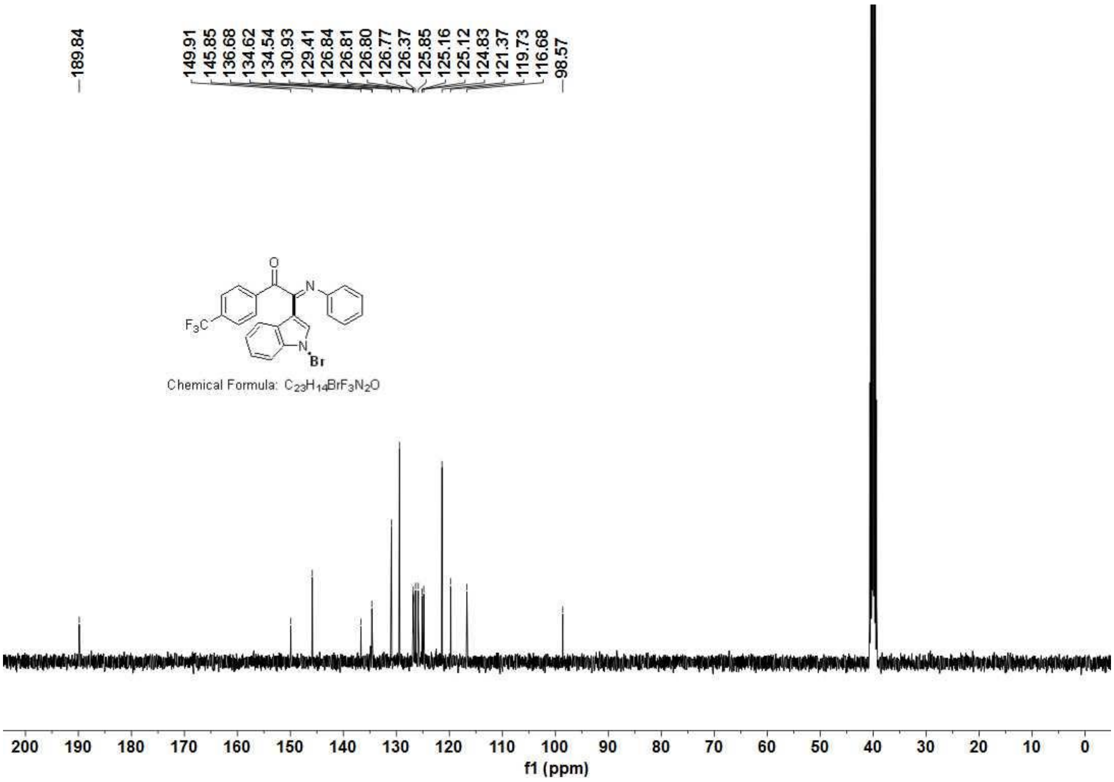

HRMS spectra

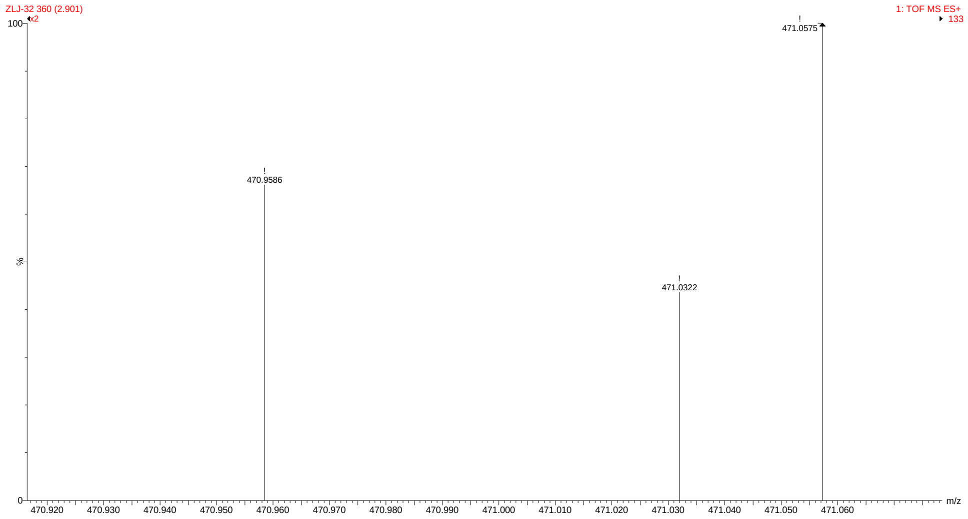

(*E*)-2-(1-bromo-1*H*-indol-3-yl)-1-(naphthalen-2-yl)-2-(phenylimino)ethan-1-one (3m)

^{1}H NMR (400 MHz, DMSO-d_6)

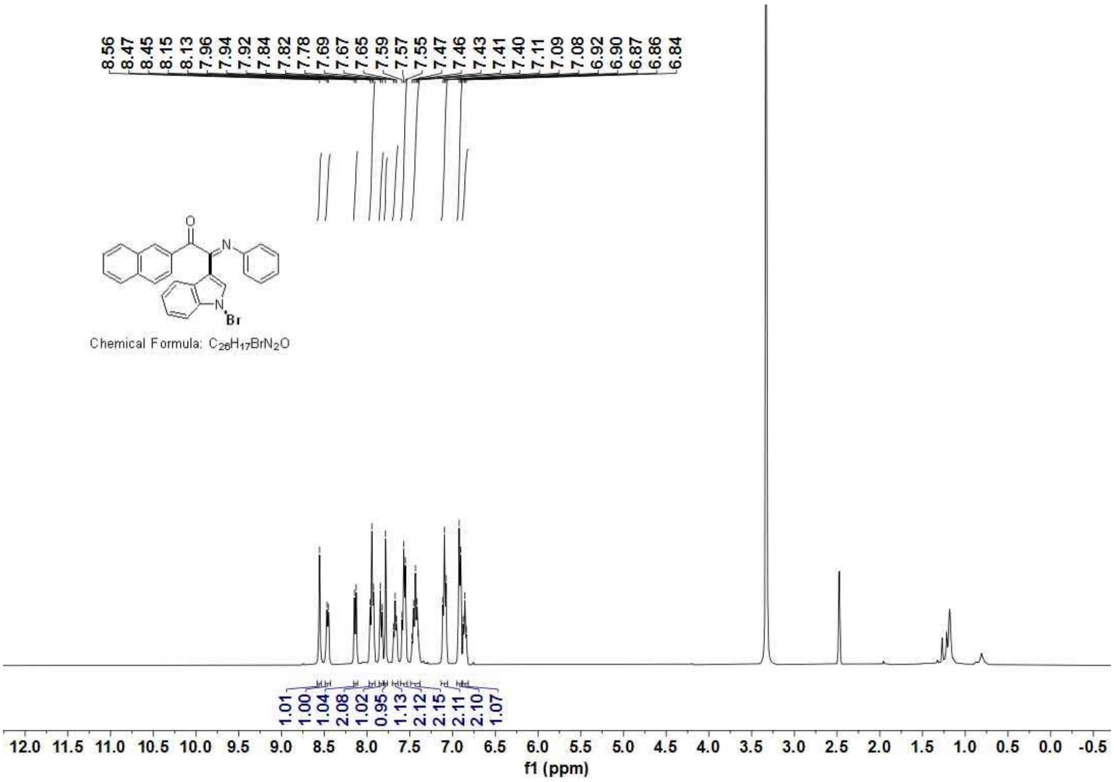

^{13}C NMR (101 MHz, DMSO-d_6)

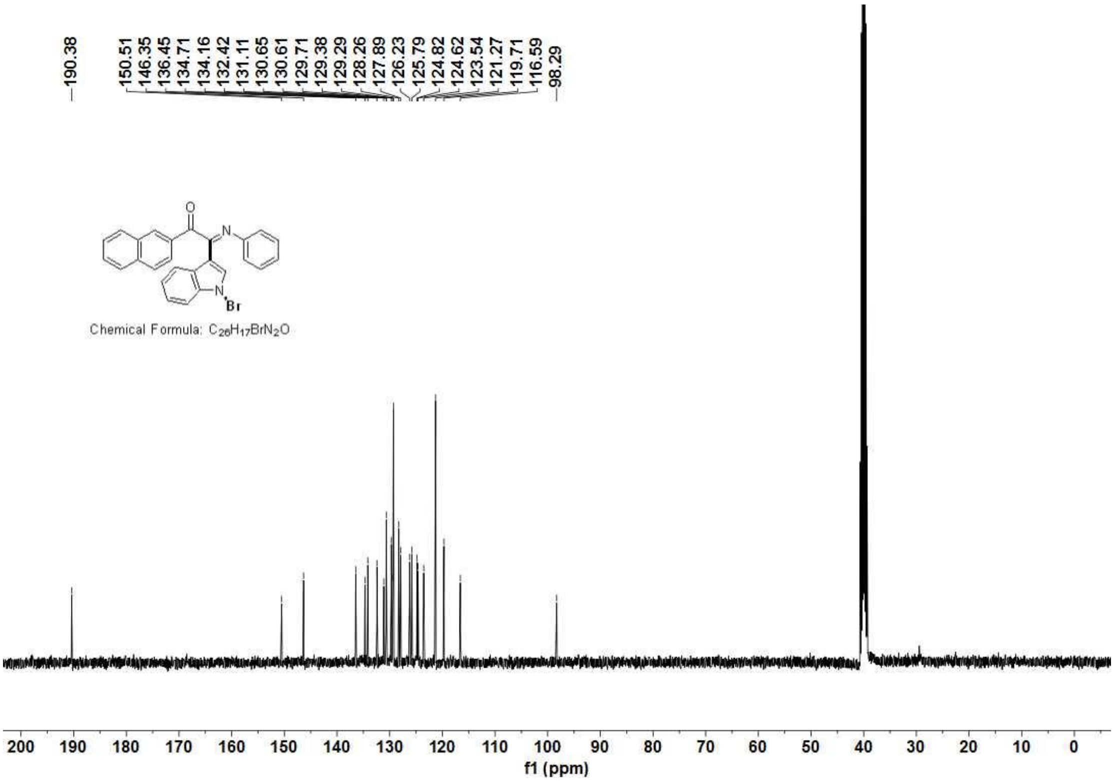

HRMS spectra

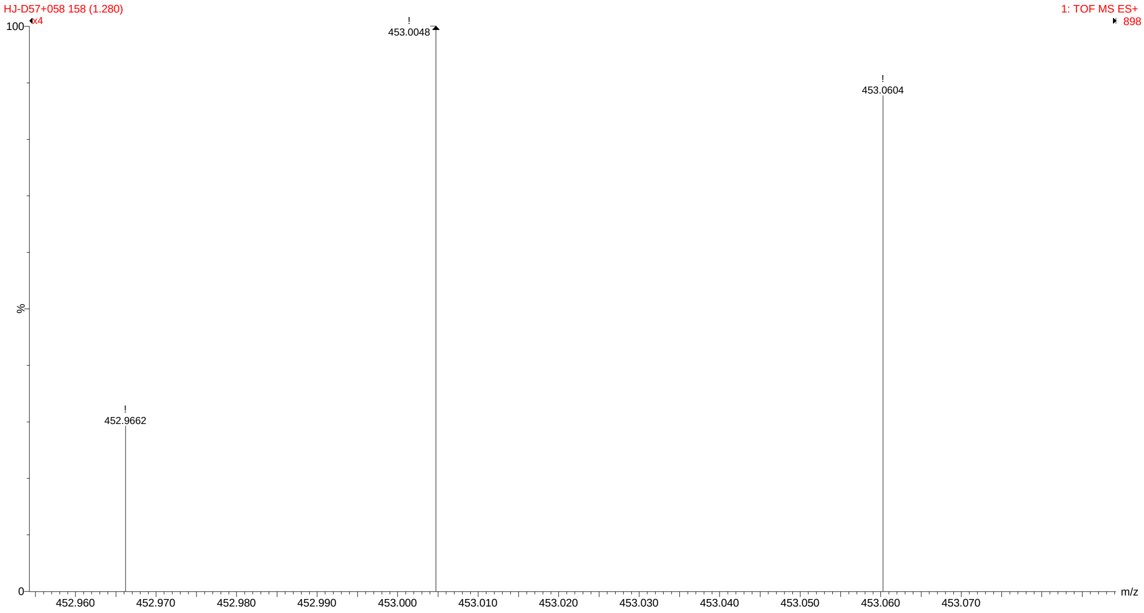

(*E*)-2-(1-bromo-1*H*-indol-3-yl)-1-phenyl-2-(m-tolylimino)ethan-1-one (3n)：

^{1}H NMR (400 MHz, DMSO-d_6)

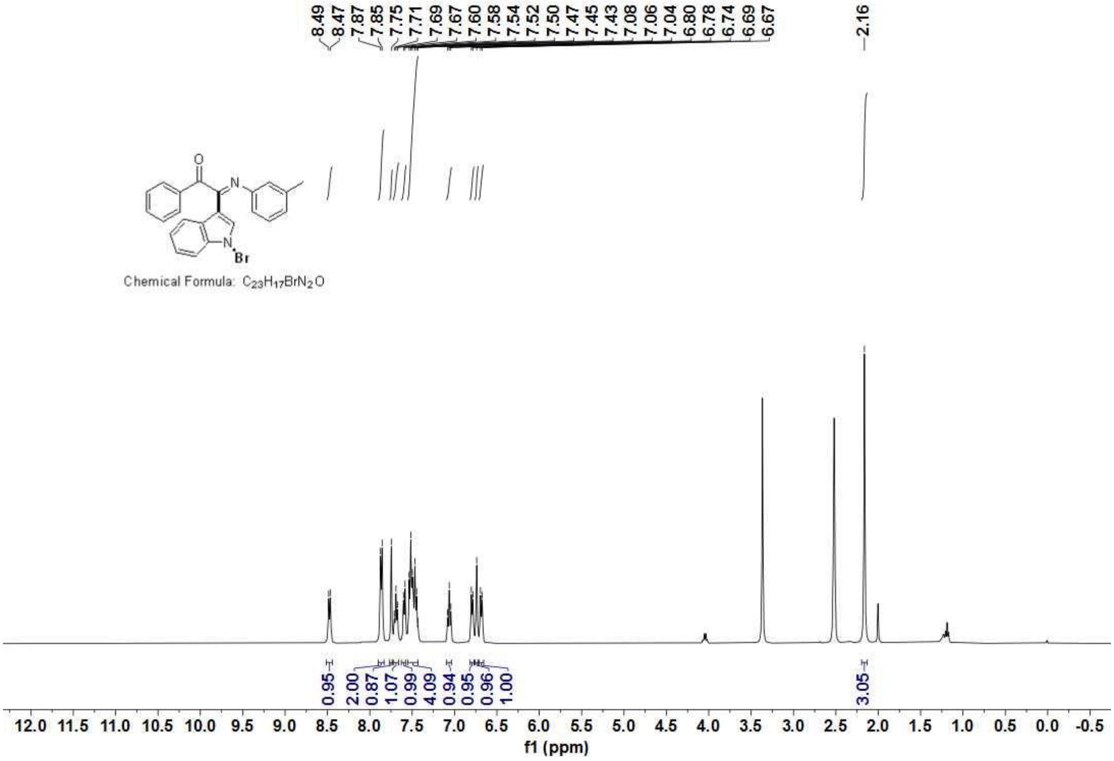

^{13}C NMR (101 MHz, DMSO-d_6)

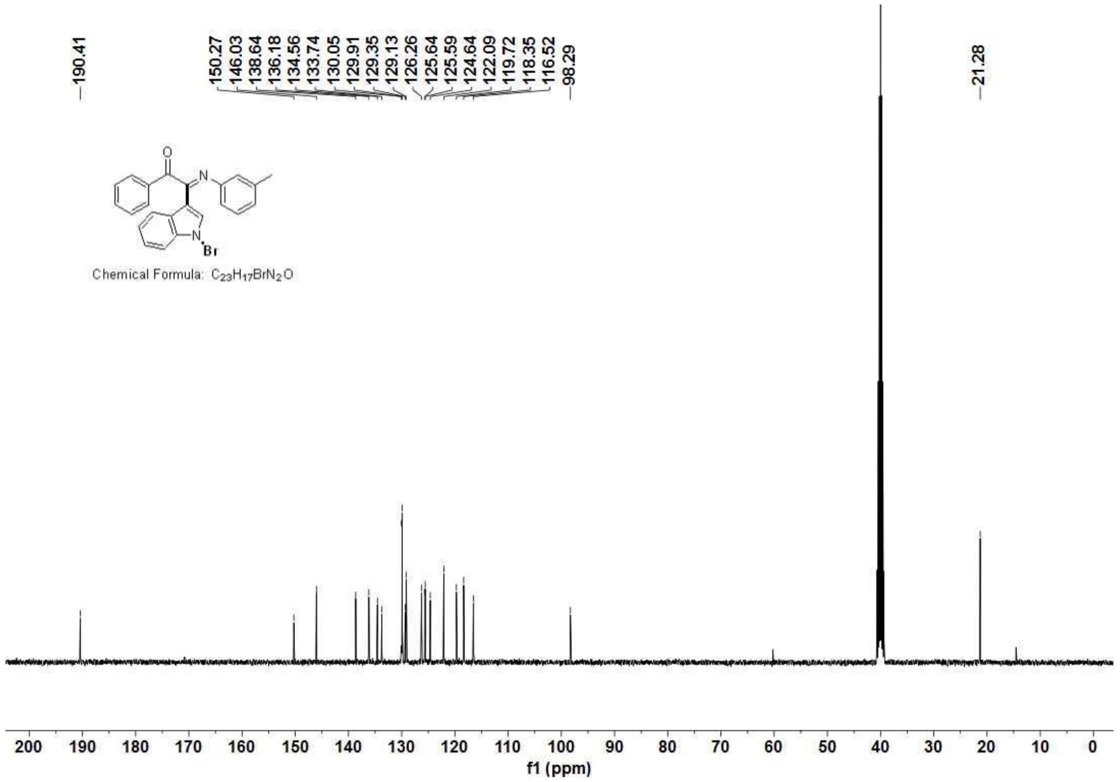

HRMS spectra

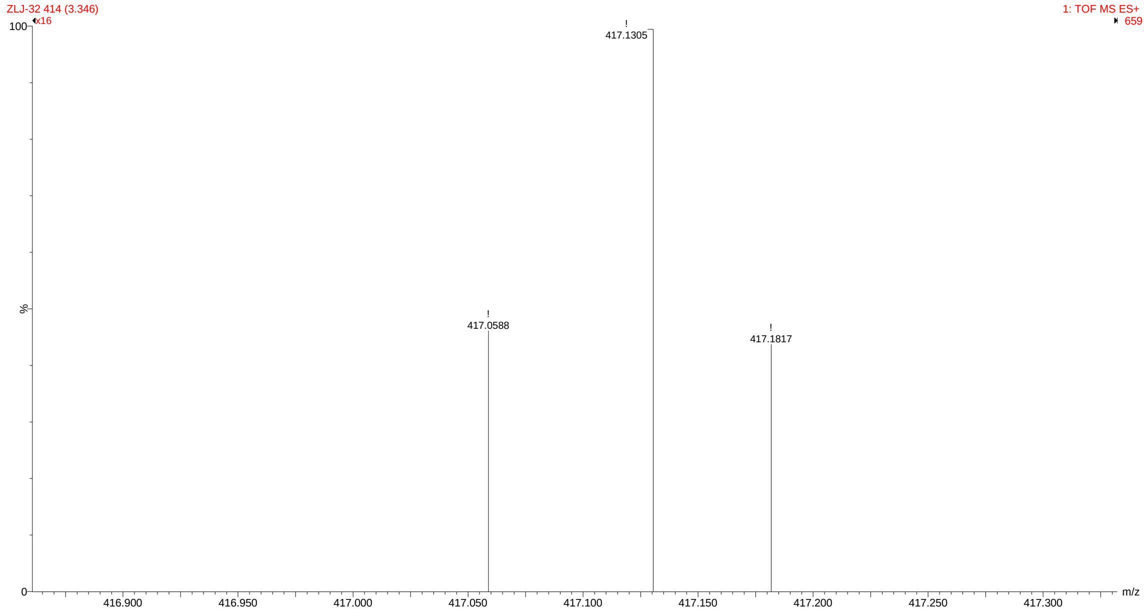

(*E*)-2-(1-bromo-1*H*-indol-3-yl)-1-phenyl-2-(p-tolylimino)ethan-1-one (3o):

^{1}H NMR (400 MHz, DMSO-d_6)

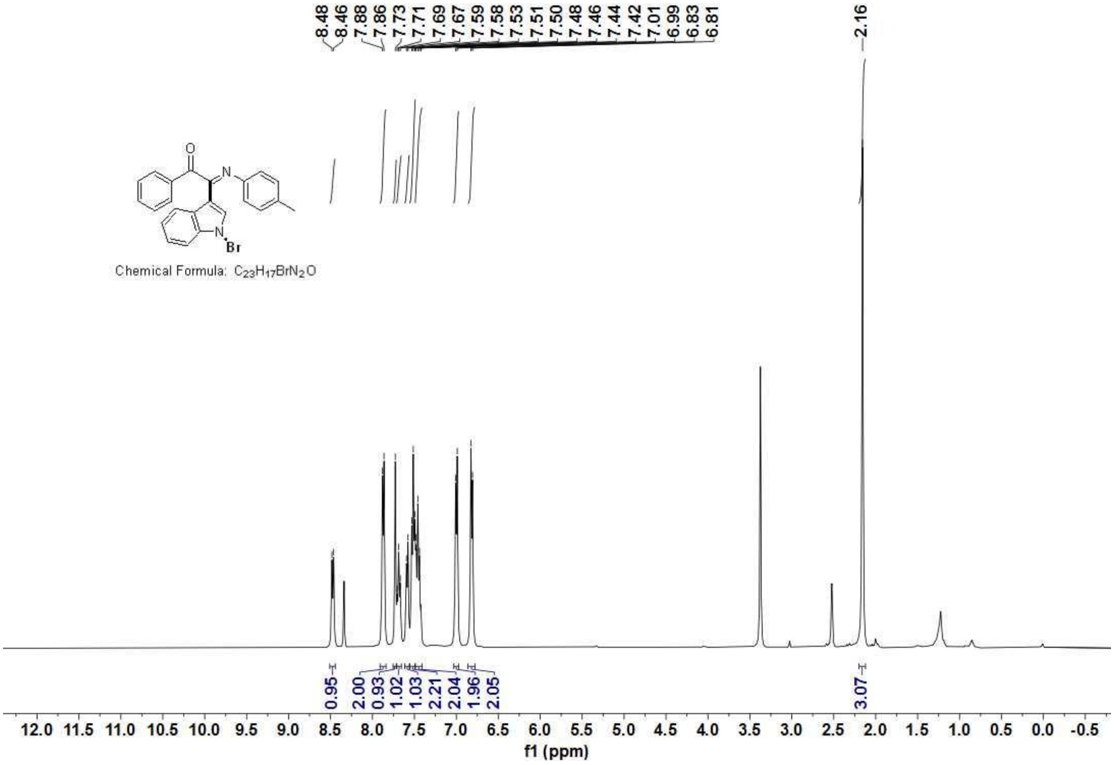

^{13}C NMR (101 MHz, DMSO-d_6)

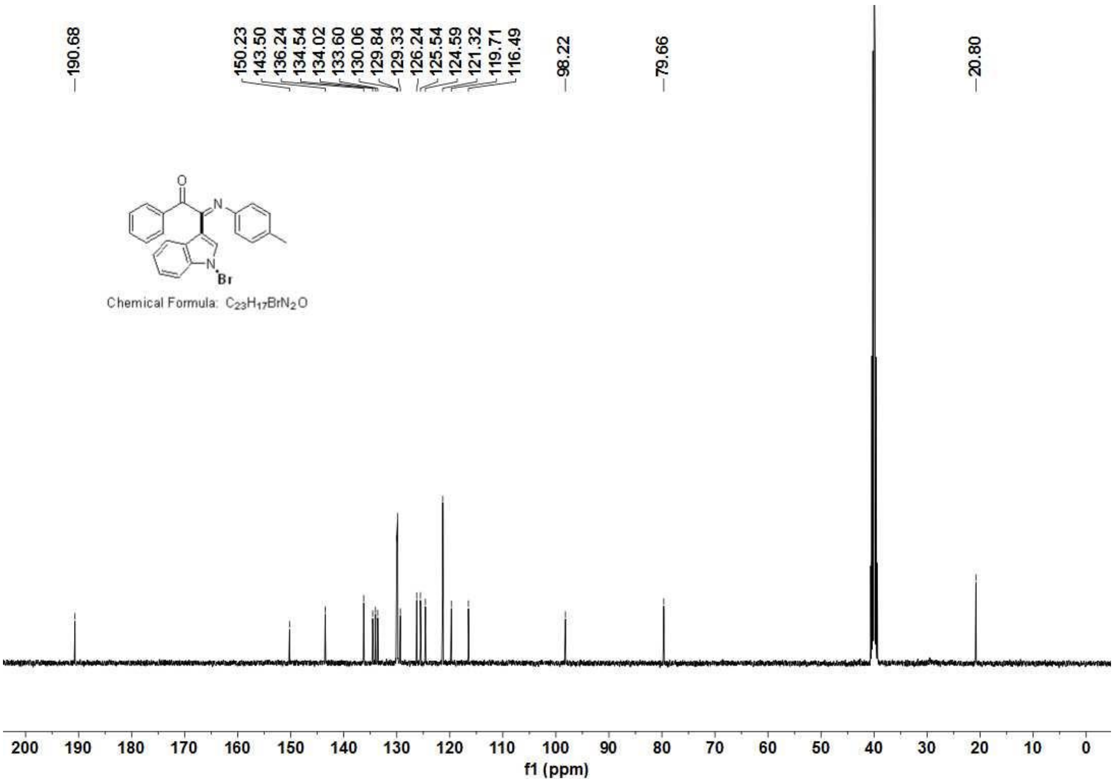

HRMS spectra

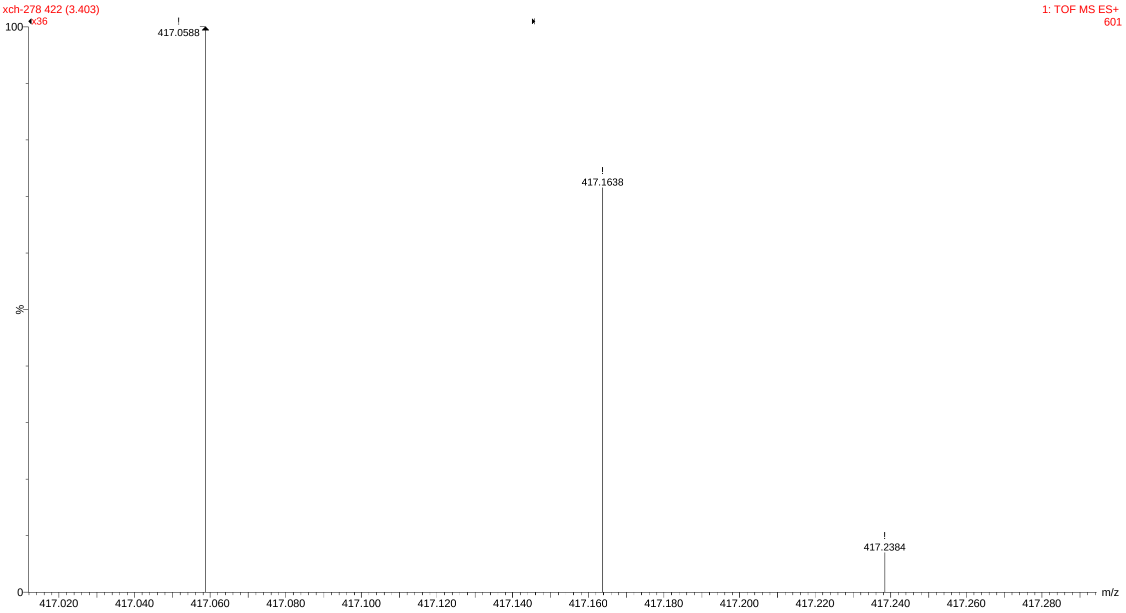

(*E*)-2-(1-bromo-1*H*-indol-3-yl)-2-((4-chlorophenyl)imino)-1-phenylethan-1-one (3p):

^{1}H NMR (400 MHz, DMSO-d_6)

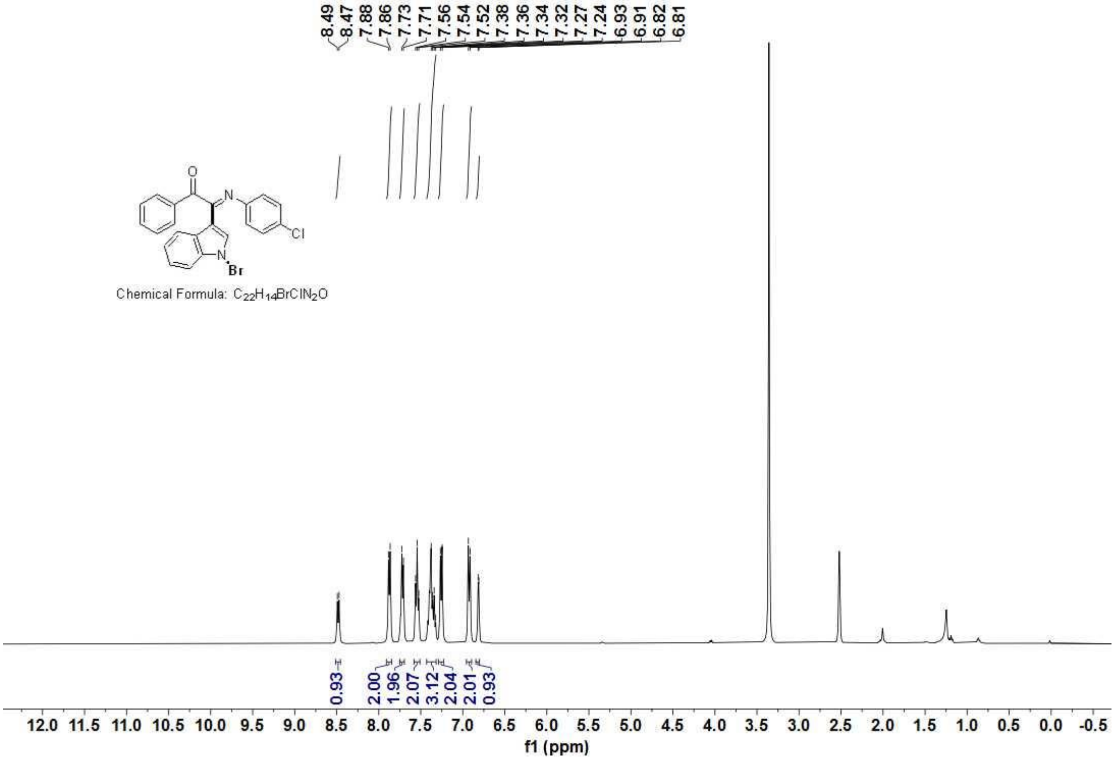

^{13}C NMR (101 MHz, DMSO-d_6)

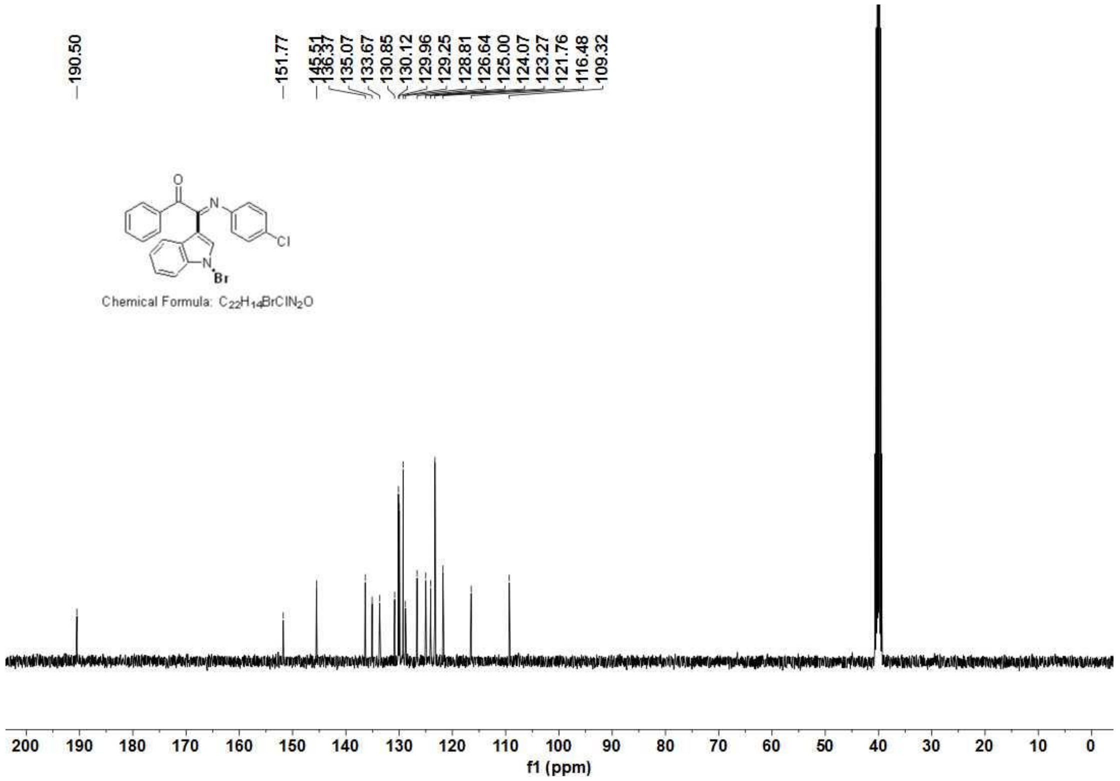

HRMS spectra

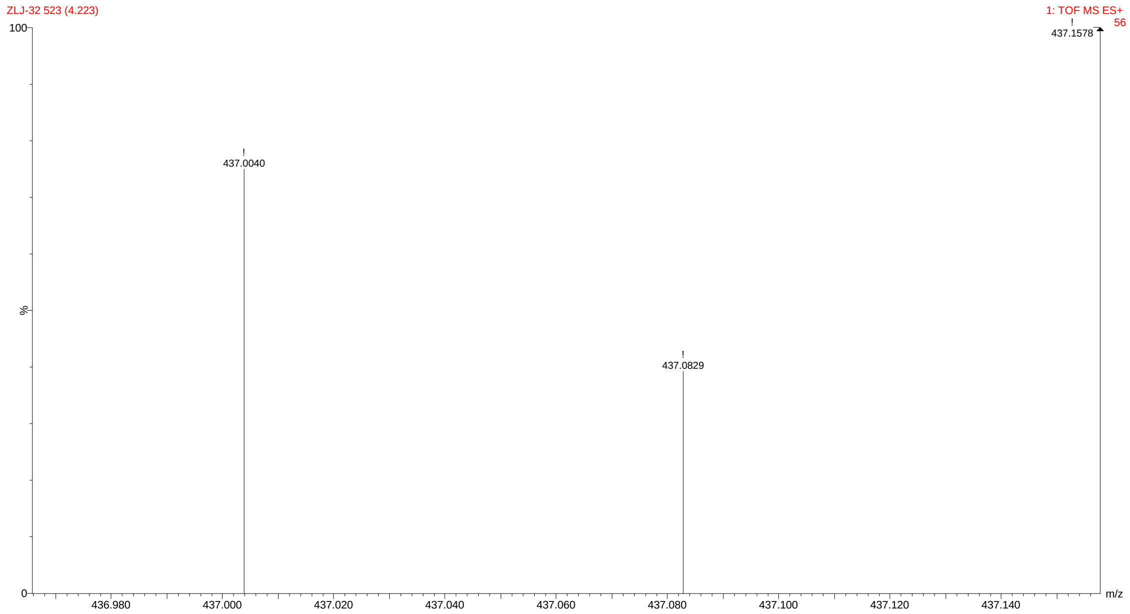

(*E*)-2-(1-bromo-5-methyl-1*H*-indol-3-yl)-1-phenyl-2-(phenylimino)ethan-1-one (3q):

^{1}H NMR (400 MHz, DMSO-d_6)

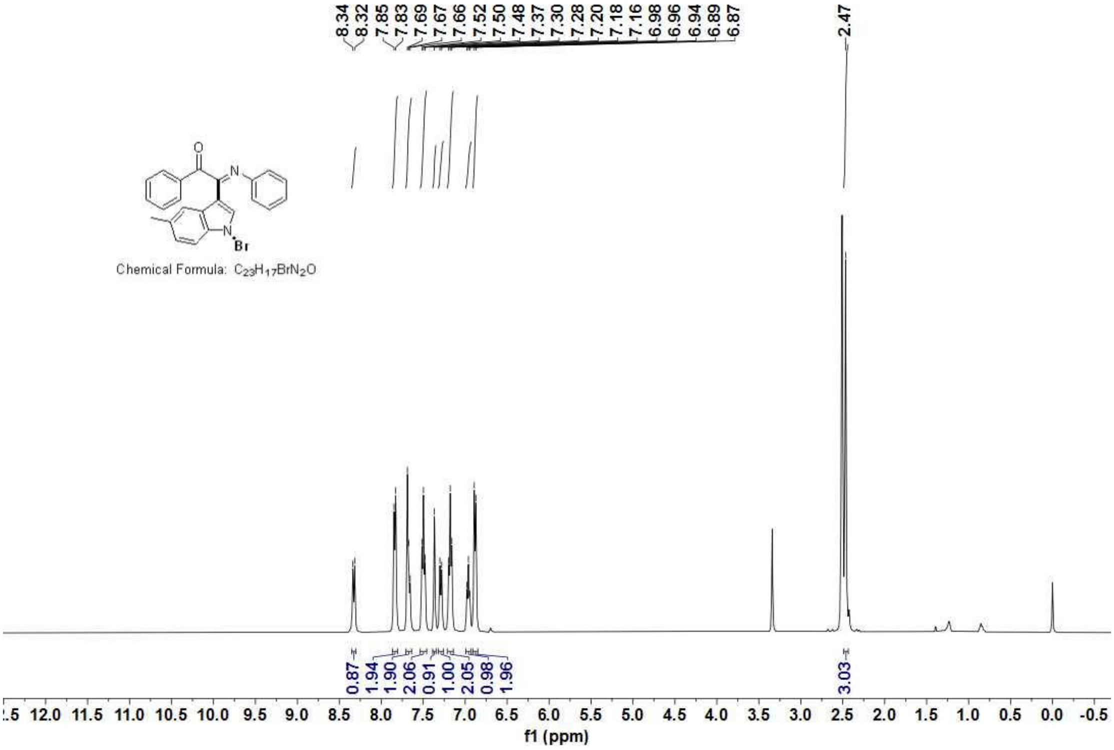

^{13}C NMR (101 MHz, DMSO-d_6)

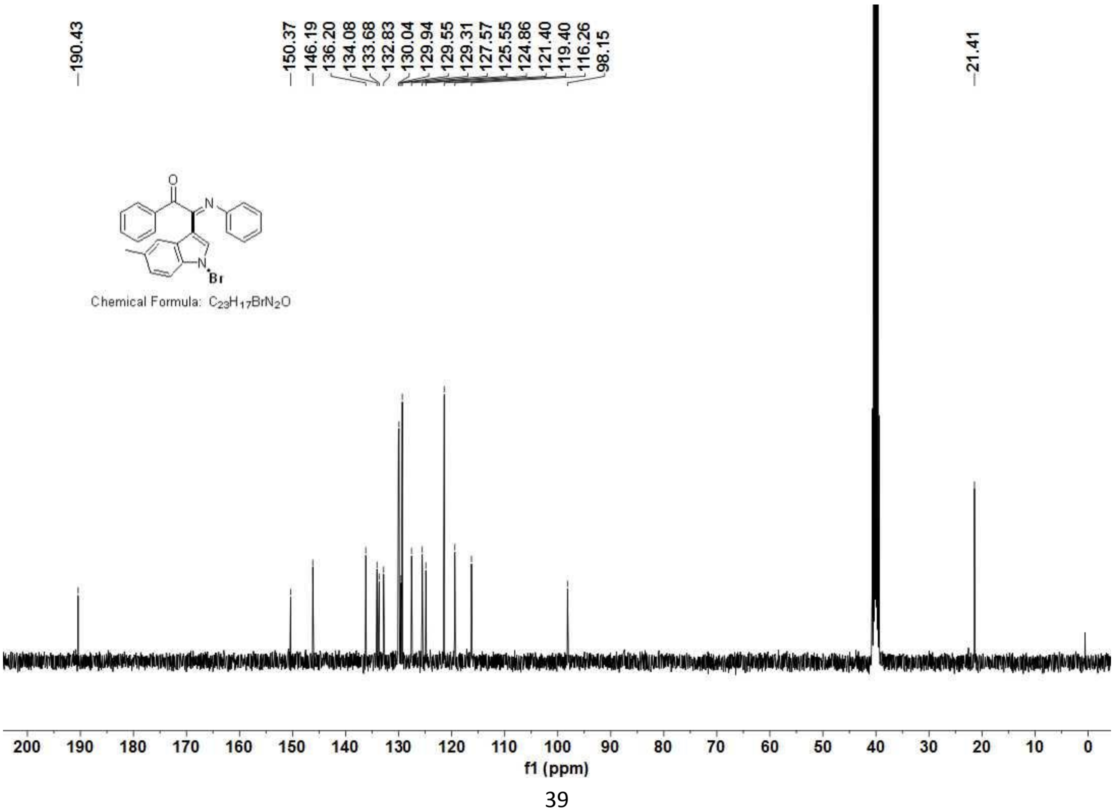

HRMS spectra

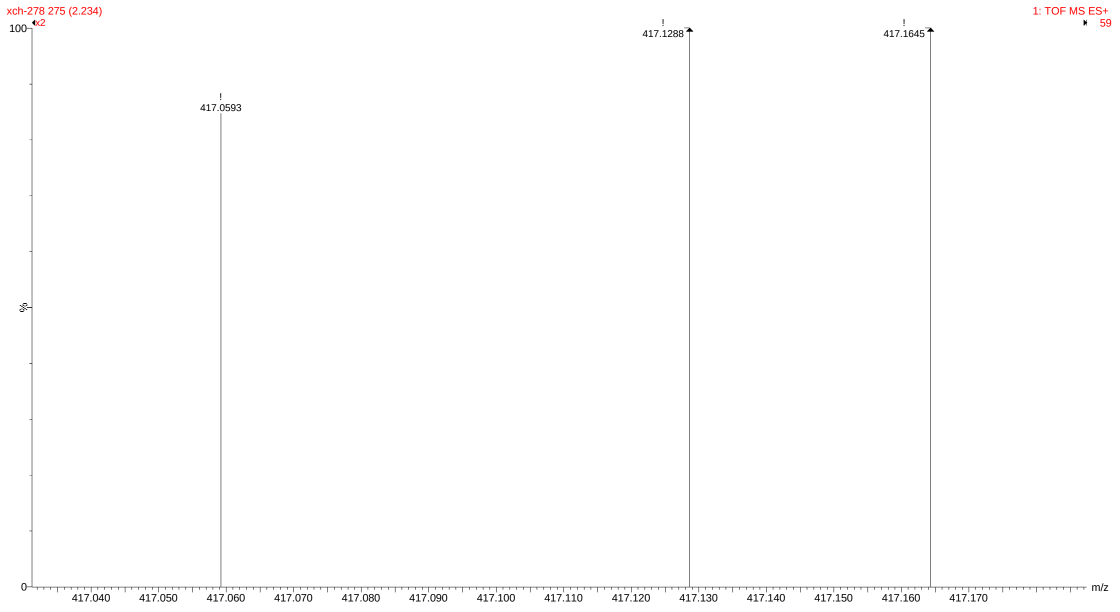

(*E*)-2-(1-bromo-6-chloro-1*H*-indol-3-yl)-1-phenyl-2-(phenylimino)ethan-1-one (3r):

^{1}H NMR (400 MHz, DMSO-d_6)

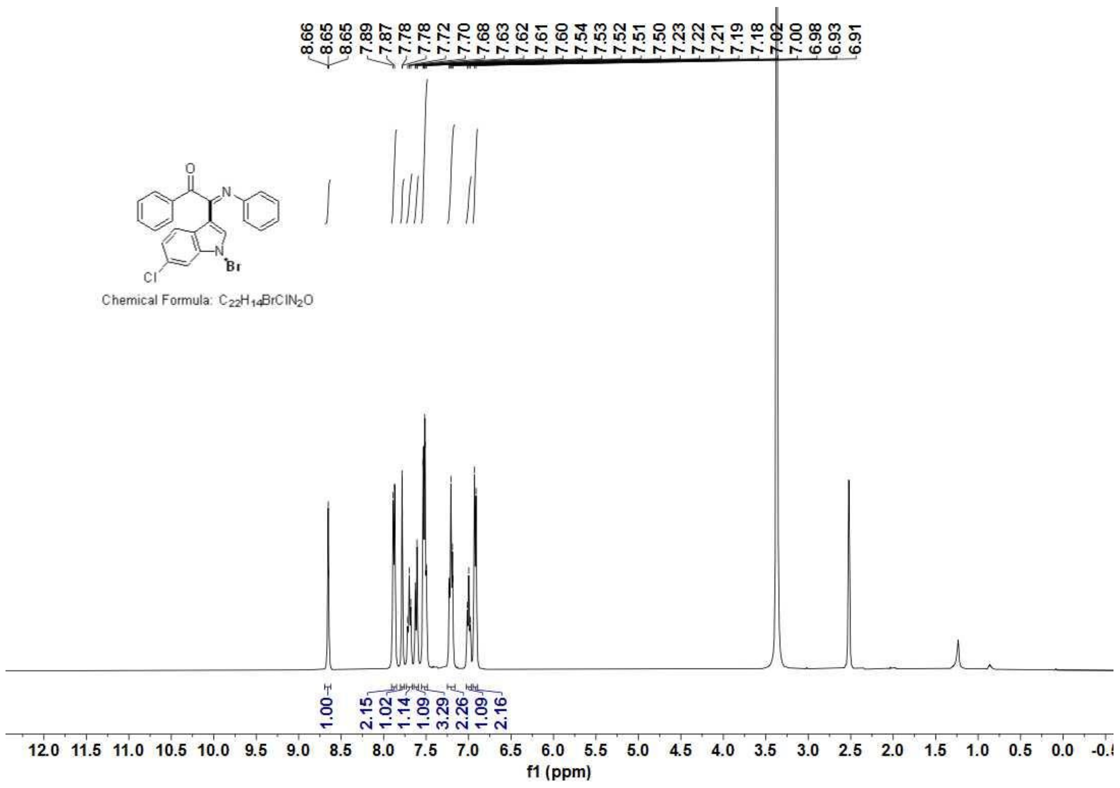

^{13}C NMR (101 MHz, DMSO-d_6)

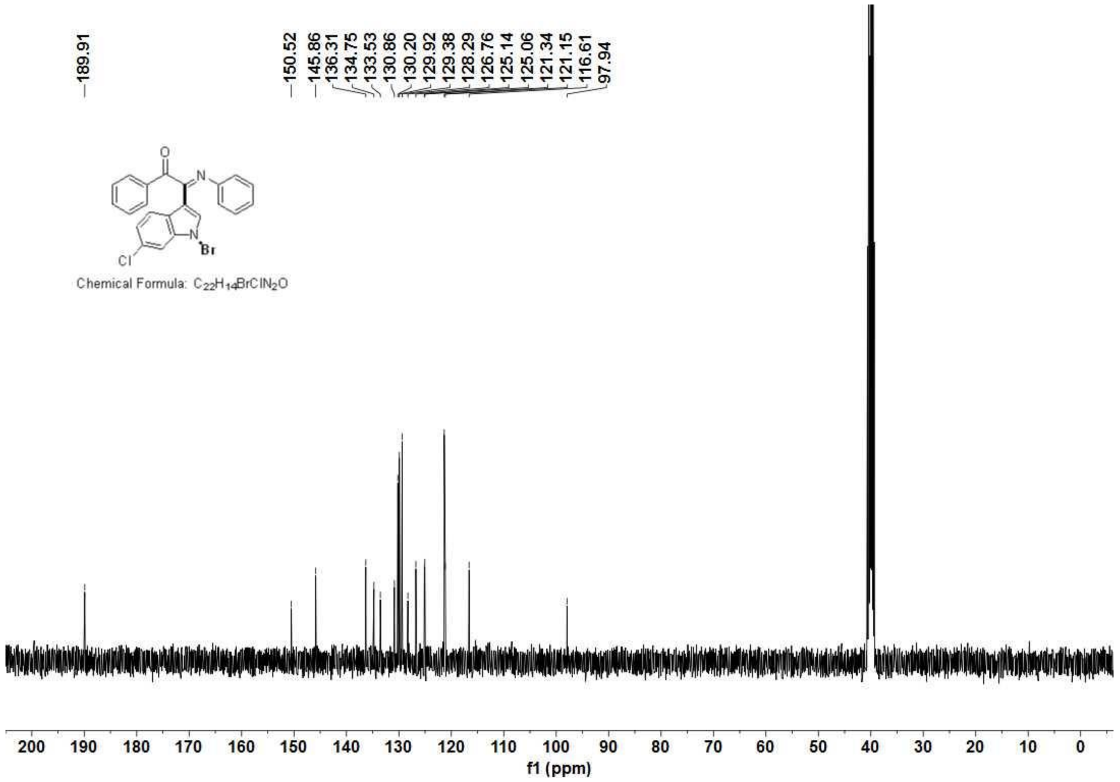

HRMS spectra

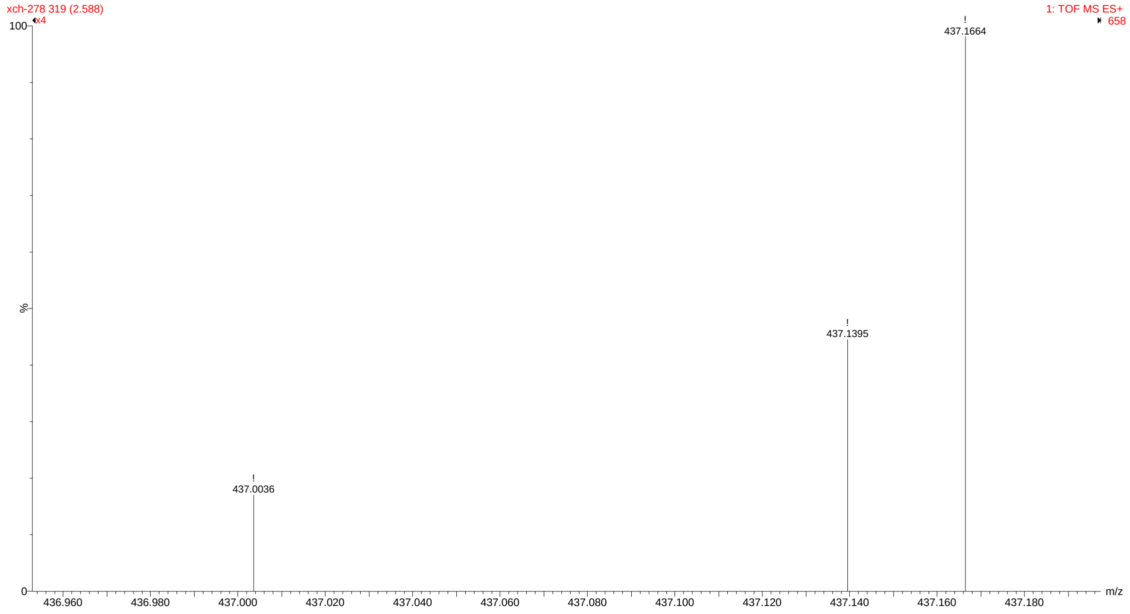

Supplement: Supplementary file 1 [file DataSheet1.pdf]
